# Intestinal epithelial cells related lncRNA and mRNA expression profiles in dextran sulphate sodium‐induced colitis

**DOI:** 10.1111/jcmm.16174

**Published:** 2020-12-09

**Authors:** Huan Liu, Teming Li, Shizhen Zhong, Min Yu, Wenhua Huang

**Affiliations:** ^1^ The Precision Medicine Institute The Third Affiliated Hospital Southern Medical University Guangzhou China; ^2^ Affiliated Traditional Chinese Medicine Hospital Southwest Medical University Luzhou China; ^3^ Department of General Surgery Xinqiao Hospital Army Medical University Chongqing China; ^4^ Guangdong Engineering Research Center for Translation of Medical 3D Printing Application Guangdong Provincial Key Laboratory of Medical Biomechanics School of Basic Medical Sciences Southern Medical University Guangzhou China; ^5^ Pathological Diagnosis and Research Center Affiliated Hospital of Guangdong Medical University Zhanjiang China

**Keywords:** inflammatory bowel disease, intestinal epithelial barrier, intestinal epithelial cells, lncRNAs, mRNAs

## Abstract

Intestinal epithelial barrier damage caused by intestinal epithelial cells (IECs) dysfunction plays a crucial role in the pathogenesis and development of inflammatory bowel disease (IBD). Recently, some studies have suggested the emerging role of long non‐coding RNAs (lncRNAs) in IBD. The aim of this study was to reveal lncRNAs and mRNA expression profiles in IECs from a mouse model of colitis and to expand our understanding in the intestinal epithelial barrier regulation. IECs from the colons of wild‐type mice and dextran sulphate sodium (DSS)‐induced mice were isolated for high‐throughput RNA‐sequencing. A total of 254 up‐regulated and 1013 down‐regulated mRNAs and 542 up‐regulated and 766 down‐regulated lncRNAs were detected in the DSS group compared with the Control group. Four mRNAs and six lncRNAs were validated by real‐time quantitative PCR. Function analysis showed that dysregulated mRNAs participated in TLR7 signalling pathway, IL‐1 receptor activity, BMP receptor binding and IL‐17 signalling pathway. Furthermore, the possibility of indirect interactions between differentially expressed mRNAs and lncRNAs was illustrated by the competing endogenous RNA (ceRNA) network. LncRNA ENSMUST00000128026 was predicted to bind to mmu‐miR‐6899‐3p, regulating Dnmbp expression. LncRNA NONMMUT143162.1 was predicted to competitively bind to mmu‐miR‐6899‐3p, regulating Tnip3 expression. Finally, the protein‐protein interaction (PPI) network analysis was constructed with 311 nodes and 563 edges. And the highest connectivity degrees were Mmp9, Fpr2 and Ccl3. These results provide novel insights into the functions of lncRNAs and mRNAs involved in the regulation of the intestinal epithelial barrier.

## 1 INTRODUCTION

Inflammatory bowel disease (IBD) is a group of chronic, non‐specific inflammatory conditions of the gastrointestinal tract that mainly include Crohn's disease (CD) and ulcerative colitis (UC).^1^, ^2^ During the last decades, the incidence and prevalence of IBD are increasing in the world, due to many factors such as environmental exposures, better detection techniques (eg colonoscopy), advances in healthcare infrastructure and so on.^3^, ^4^, ^5^ However, the precise pathogenesis of IBD is still unknown. Recent evidence implicates that the disruption of intestinal epithelial barrier contributes to many intestinal diseases, including IBD.^6^, ^7^, ^8^

The intestinal epithelial barrier consists of different types of intestinal epithelial cells (IECs) with intact tight junction (TJ). In the intestinal lumen, this barrier acts as the first physical and immunological protective wall against the toxins and pathogenic organisms.^9^ Excessive death of IEC, altered expression and distribution of TJ proteins can cause the intestinal epithelial damage, which is characteristic of IBD.^10^, ^11^, ^12^, ^13^ For example, increased apoptosis was reported in the intestinal epithelium of both patients with UC or CD.^10^, ^14^, ^15^, ^16^, ^17^, ^18^ In addition, clinical studies showed that changes in the expression and distribution of TJ proteins led to an altered TJ structure and barrier dysfunction in the active Crohn's disease.^13^

Long non‐coding RNAs (lncRNAs) are RNA transcripts more than 200 bp in length, but lack of protein‐coding capacity.^19^ Accumulating evidence has shown that lncRNAs act as important regulators in a variety of physiological and pathological processes, such as chromatin modification, transcriptional regulation, post‐transcriptional regulation and so on.^19^, ^20^ Abnormal expression of lncRNA is closely related to the development of various diseases.

Increasing evidence suggests that lncRNA plays an important role in the pathogenesis of IBD.^21^ For example, lncRNA interferon‐γ‐antisense 1 (IFNG‐AS1), which is up‐regulated in the intestinal mucosa of patients with actively inflamed IBD, has been reported to be a mediator of an inflammatory signalling cascade in IBD pathophysiology.^22^, ^23^ Interestingly, several studies have shown that some lncRNAs are involved in the modulation of intestinal epithelial barrier function. For instance, the apoptosis of IECs was reported to be regulated by lncRNA BC012900.^24^ Overexpression of lncRNA H19 decreased the expression of TJ protein, zonula occludin 1 (ZO‐1).^25^ However, studies investigating the role of lncRNAs in IBD are still limited.

To date, there have been several studies that used high‐throughput RNA‐seq analysis or microarray technology to determine the coding or non‐coding gene differences both in mouse colitis models and in human IBD samples.^26^, ^27^, ^28^, ^29^, ^30^, ^31^ However, in these previous studies, the whole colon tissue was utilized for high‐throughput RNA‐seq or microarray analysis. Given the complexity of the cell types of the whole colon, such as IECs, intestinal intraepithelial lymphocytes (IELs) and lamina propria lymphocytes (LPLs), it is necessary to purify and collect different cell populations for the characterization of lncRNAs.

In this study, we focused on IECs and sought to determine the dysregulated lncRNAs and mRNAs that may be involved in the regulation of intestinal epithelial barrier in IBD. Therefore, we established the dextran sulphate sodium (DSS)‐induced mouse model of colitis and isolated the IECs of murine colon for high‐throughput RNA‐sequencing analysis. A total of 254 up‐regulated and 1013 down‐regulated mRNAs and 542 up‐regulated and 766 down‐regulated lncRNAs were detected in the DSS group compared with the Control group. Subsequently, the gene ontology (GO) analysis and Kyoto Encyclopedia of Genes and Genomes (KEGG) enrichment analysis showed that differentially expressed (DE) mRNAs participated in TLR7 signalling pathway, IL‐1 receptor activity, BMP receptor binding, IL‐17 signalling pathway, ECM‐receptor interaction and so on. Then, the competing endogenous RNA (ceRNA) network was constructed to predict the interactions of lncRNA‐miRNA‐mRNA. LncRNA ENSMUST00000128026 was predicted to bind to mmu‐miR‐6899‐3p, regulating Dnmbp expression. LncRNA NONMMUT143162.1 was predicted to competitively bind to mmu‐miR‐6899‐3p, regulating Tnip3 expression. Moreover, the protein‐protein interaction (PPI) network was constructed with 311 nodes and 563 edges to identify the interactions between DE mRNAs. And the highest connectivity degrees were Mmp9, Fpr2 and Ccl3.

## 2 MATERIAL AND METHODS

### 2.1 Animal model

Mouse model of DSS‐induced colitis was established according to the protocol in our previous study.^32^, ^33^, ^34^ Male, 6‐ to 8‐week‐old C57BL/6 mice were obtained from the Experiment Animal Center (Army Medical University, Chongqing, China). A total of 10 mice per group were used to establish animal models. Control mice were provided with distilled water for 7 days. And mice in the DSS group were provided with drinking water containing 3% DSS for 7 days. Then, mice were sacrificed for haematoxylin‐eosin (H&E) staining and IEC isolation. All procedures were approved by the University Committee on the Use and Care of Animals of the Army Medical University.

### 2.2 Isolation and purification of IECs

Intestinal epithelial cells isolation was performed as previously described.^33^ Briefly, the colon was removed and placed in a tissue culture medium (RPMI 1640, with 10% foetal calf serum). Then, the colon was cut into 5‐mm pieces followed by extensively rinsed with ice‐cold PBS containing 2% foetal calf serum. The rinsed pieces were then incubated in Ca^2+^‐ and Mg^2+^‐free PBS containing 5 mM EDTA, 2 mM DTT and 10% foetal calf serum for 0.5 hour at 37°C with continuous brisk stirring. Then, the supernatant was collected and filtered through both 70 and 30 μM MACS SmartStrainers (Miltenyi Biotec) to remove debris and pellets. After centrifugation, the IECs were purified by 40% Percoll (GE Healthcare Bio‐sciences), and then, the CD3e MicroBead Kit (Miltenyi Biotec) was used to eliminate CD3^+^ intraepithelial lymphocytes according to the manufacturer's instructions. Finally, IECs in the suspension were collected for flow cytometric analysis and high‐throughput sequencing.

Then, the purity of the IECs was detected by flow cytometric analysis. The IECs were stained with Bv421 anti mouse E‐Cadherin (BD Biosciences) and FITC anti mouse CD3 (Biolegend) according to the manufacturer's protocol. The acquisition and analysis were performed using Beckman Coulter Gallios Flow Cytometer (Beckman Coulter).

### 2.3 RNA isolation and high‐throughput sequencing

Total RNA was isolated using RNeasy mini kit (Qiagen). Then, the RNA concentration and quality were determined by the Qubit^®^2.0 Fluorometer (Life Technologies) and the Nanodrop One spectrophotometer (Thermo Fisher Scientific Inc). Integrity of the total RNA was assessed by the Agilent 2100 Bioanalyzer (Agilent Technologies Inc), and samples with RNA integrity number (RIN) values >7.0 were used for sequencing. RNA‐seq strand‐specific libraries were constructed using the VAHTS Total RNA‐seq (H/M/R) Library Prep Kit (Vazyme) according to the manufacturer's instructions. Briefly, RNA was purified by magnetic beads after removal of rRNA. And the RNA was then cleaved into small pieces by divalent cations for 8 minutes at 94°C. Using reverse transcriptase and random primers, the cleaved RNA fragments were copied into the first‐strand cDNA. Subsequently, the second‐strand cDNA synthesis was performed with DNA Polymerase I and RNase H. After that, these cDNA fragments went through the terminal repair process, the addition of a single ‘A’ base, and ligation of the adapters. The product was purified and enriched with PCR, and the final cDNA library was established. Purified libraries were quantified by Qubit^®^ 2.0 Fluorometer (Life Technologies). And the size distribution of the purified libraries was validated by Agilent 2100 bioanalyzer (Agilent Technologies).

Cluster was generated by cBot with the library diluted to 10 pM before sequencing on the NovaSeq 6000 (Illumina). Paired‐end sequence files were mapped to the reference genome (mmu GRCm38.91) using Hierarchical Indexing for Spliced Alignment of Transcripts (Hisat2, version 2.0.5). The output sequencing alignment/map (SAM) files were converted to binary alignment/map (BAM) files and sorted using SAMtools (version 1.3.1). Gene abundance was expressed as fragments per kilobase of exon per million reads mapped (FPKM). Stringtie software was used to count the fragment within each gene, and trimmed mean of *M* values (TMM) algorithm was used for normalization.

The high‐throughput sequencing and bioinformatics analysis were performed by Shanghai Sinomics Corporation. Each group had three biological replicates for RNA‐seq. The raw data were uploaded to Sequence Read Archive (SRA) of NCBI (SRA accession: PRJNA637224).

### 2.4 Analysis of differentially expressed genes (DEGs)

The analysis for DE mRNA and DE lncRNA was performed using R package edgeR. Differentially expressed genes with |log2(FC)| value >1 and *P* < .05 were considered as significantly modulated and retained for further analysis. This choice is motivated by the decision to maximize the sensitivity of this analysis, in order to perform a massive screening and identify candidate genes to be validated with a wider sample population with real‐time PCR analysis.

### 2.5 GO and KEGG pathway analysis

The GO analysis (http://www.geneontology.org) for biological processes, cellular components and molecular function and the KEGG pathway analysis (http://www.genome.ad.jp/kegg) were performed via enrich R package. *P* < .05 was considered to be a statistically significant enrichment.

### 2.6 Quantitative real‐time polymerase chain reaction (qRT‐PCR) confirmation

Six lncRNAs and four mRNAs were selected for validation. The qRT‐PCR was performed as previously described.^33^ Total RNA was extracted from the IECs using RNAiso Plus (Takara), according to the manufacturer's instructions. The total RNA was reverse transcribed into complementary DNA (cDNA) using a SuperScript FirstStrand Synthesis System RT‐PCR Kit (Invitrogen). The sequences of the primers used in the present study were listed in Table S1. The relative gene expression ratio was analysed using the $2^{‐\Delta \Delta C_{t}}$ method.^35^ Glyceraldehyde 3‐phosphate dehydrogenase (GAPDH) was used as a reference for normalization. The qRT‐PCR results were statistically tested with a Student's *t* test. A *P* < .05 was considered statistically significant.

### 2.7 Construction of the ceRNA network

With a threshold of *P*‐value <.05 and |Log2 FC| > 8, 20 mRNAs that would be further studied were selected. To identify the interactions between the chosen 20 mRNAs and other lncRNA or miRNA, we constructed ceRNA networks. The miRNA‐mRNA and miRNA‐lncRNA interactions were identified through the bioinformatics algorithm miRanda. According to lncRNA‐miRNA pairs and miRNA‐mRNA pairs above, ceRNA network maps for top 500 lncRNA‐miRNA‐mRNA (sum Max Energy ≤−54.37) were illustrated using Cytoscape software (version 3.7.1).

### 2.8 Construction of the PPI network

DE mRNAs with a threshold of *P*‐value <.05 and fold change >4 were selected. The Search Tool for the Retrieval of Interacting Genes (STRING, version 11.0) database was used to construct PPI network. The top 20 high‐degree hub nodes were chosen, and the PPI networks were visualized using Cytoscape software. Subsequently, the plug‐in Molecular Complex Detection (MCODE, version 1.5.1) was used to screen the most significant functional modules of PPI networks in Cytoscape. In addition, GO analysis and KEGG pathway analysis were performed for DE mRNAs in the modules.

## 3 RESULTS

### 3.1 Profiles of DE mRNAs and lncRNAs

C57BL/6J wild‐type (WT) mice were treated with 3% DSS for 7 days; changes in bodyweight, colon length, mortality and histology between the wild‐type mice (Control group) and DSS‐induced mice (DSS group) confirmed the successful induction of colitis. The DSS group exhibited significant weight loss after 3‐4 days of DSS treatment (Figure S1A). In addition, colon length was decreased in the DSS group (Figure S1B). And there was a 23% rate of mortality in DSS group (Figure S1C). As shown in Figure S1D, remarkable mucosal inflammatory infiltration also occurred in the colon mucosa from DSS group. Then, intestinal epithelial cells (IECs) were isolated from the colons of the two groups. The epithelial cell marker E‐Cadherin was used to identify the purity of the IECs. As shown in Figure S1E, the purity of the IECs is about 96%. Then, a high‐throughput sequencing was used to detect the levels of mRNAs and lncRNAs in IECs from the Control group and DSS group.

**FIGURE 1:**
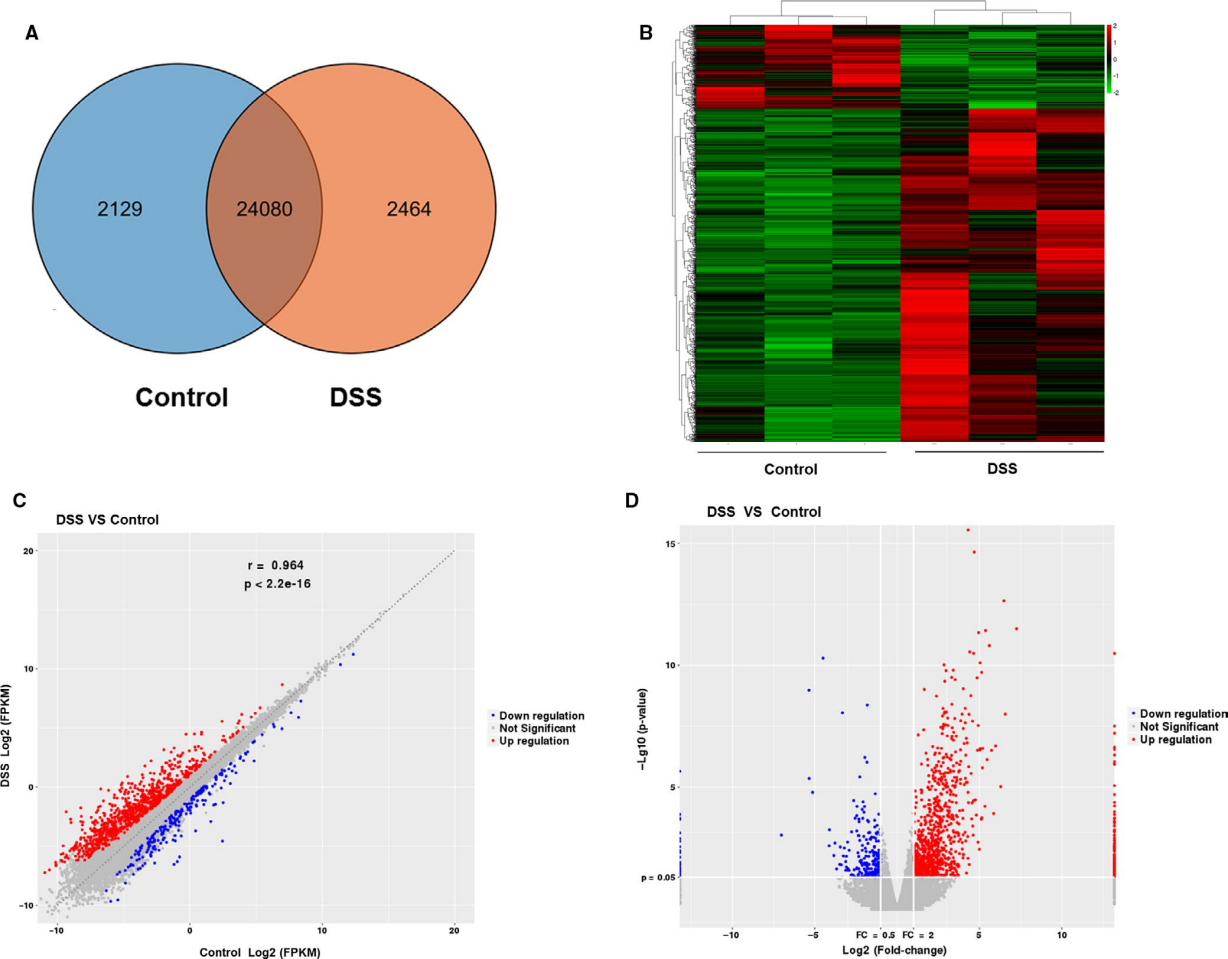
DE mRNAs in IECs from the colon of DSS‐treated mice and Control mice. A, The number of overlapping mRNAs in Control group and DSS group was displayed by Venn diagram. B, Hierarchical clustering analysis of differentially expressed mRNAs between DSS group and Control group was showed by the heat map. Red colour represents up‐regulated mRNAs, and the green colour represents down‐regulated mRNAs. Up‐regulated and down‐regulated mRNAs were showed by the scatter plot (C) and volcano plot (D). Red points indicate up‐regulated mRNAs, and blue points indicate down‐regulated mRNAs

A total of 28 673 mRNAs were detected in the Control group and DSS group. As shown in Figure 1A, 2129 were detected only in the Control group, 2464 were detected only in the DSS group, and 24 080 were detected in both groups. Then, using the following criteria: *P*‐value <.05 and fold change >2, we identified 254 up‐regulated mRNAs and 1013 down‐regulated mRNAs in the DSS group compared with the Control group. A heat map was used to show the hierarchical clustering features of altered mRNAs (Figure 1B). The variation of mRNA expression between the DSS group and the Control group was assessed by the scatter plot (Figure 1C). The volcano plot was used to visualize the differential mRNA expression between two groups (Figure 1D).

**FIGURE 2:**
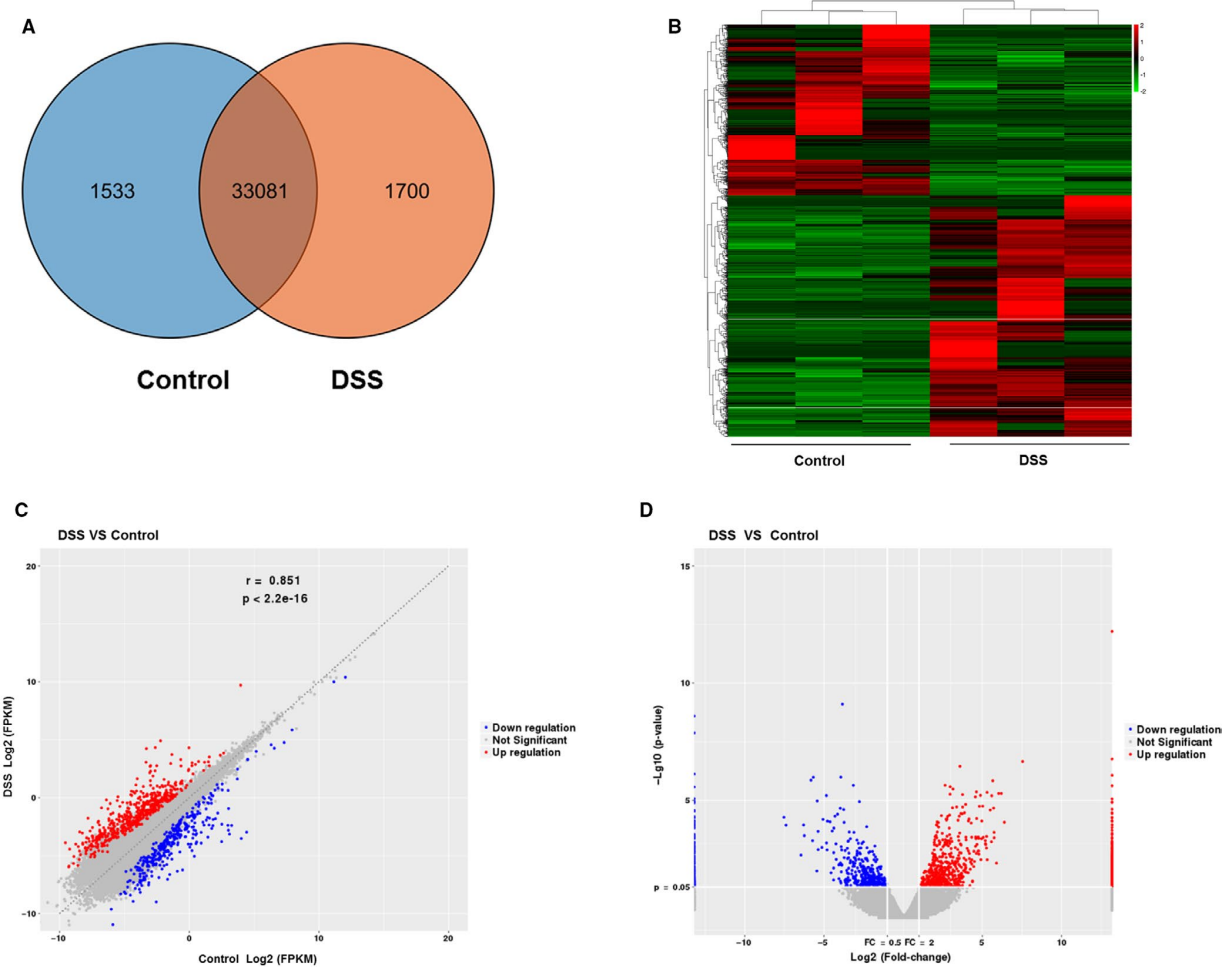
DE lncRNAs in IECs from the colon of DSS‐treated mice and Control mice. A, The number of overlapping lncRNAs in Control group and DSS group was displayed by Venn diagram. B, Hierarchical clustering analysis of differentially expressed lncRNAs between DSS group and Control group was showed by the heat map. Red colour represents up‐regulated lncRNAs, and the green colour represents down‐regulated lncRNAs. Up‐regulated and down‐regulated lncRNAs were showed by the scatter plot (C) and volcano plot (D). Red points indicate up‐regulated lncRNAs, and blue points indicate down‐regulated lncRNAs

For lncRNAs, a total of 36 314 lncRNAs were detected in the Control group and the DSS group. A total of 1533 were detected only in the Control group, 1700 were detected only in the DSS group, and 33 081 were detected in both groups (Figure 2A). A total of 542 up‐regulated lncRNAs and 766 down‐regulated lncRNAs were identified in the DSS group compared with the Control group (*P*‐value <.05 and fold change >2). As shown in Figure 2B, the hierarchical clustering features of altered lncRNAs were presented in a heat map. The variation of lncRNA expression between the DSS group and the Control group was assessed by the scatter plot (Figure 2C). The volcano plot was used to visualize the differential lncRNA expression between two groups (Figure 2D). All dysregulated lncRNAs between the DSS group and the Control group were displayed in Table S2.

### 3.2 Features of DE lncRNAs

**FIGURE 3:**
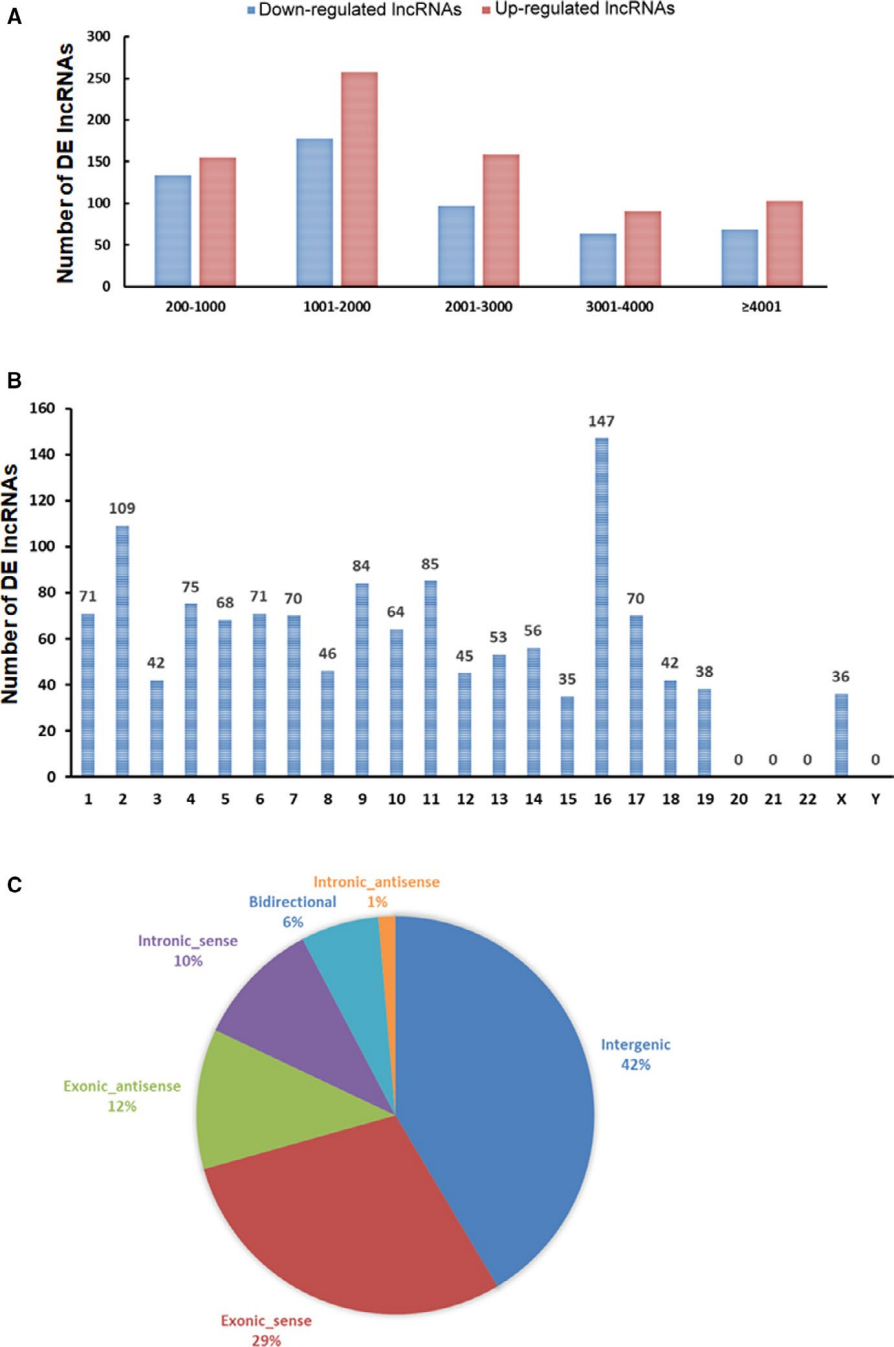
The expression characteristics of DE lncRNAs. A, Length distribution of the differentially expressed lncRNAs. B, Chromosome distribution of the differentially expressed lncRNAs. C, DE lncRNAs were classified on the basis of their genomic architecture

In order to detect the expression characteristics of DE lncRNAs, the length distribution and chromosome distribution of the up‐regulated and down‐regulated lncRNAs were analysed. DE lncRNAs were mainly concentrated between 1000 and 2000 bp in length (Figure 3A). Based on the association with neighbouring protein‐coding genes by genomic architecture, lncRNAs can be placed into one or more of five broad categories: sense, antisense, bidirectional, intronic or intergenic.^36^, ^37^ We then analysed the frequency distribution of DE lncRNAs in each category. As shown in Figure 3B, the fractions of exonic_sense, intronic_sense, exonic_antisense, intronic_antisense, bidirectional and intergenic lncRNAs were 29%, 12%, 12%, 1%, 6% and 42%, respectively. In addition, the chromosome distribution of DE lncRNAs was shown in Figure 3C. A total of 147 DE lncRNAs were located at 16 chromosome, whereas no DE lncRNAs were located at 20 chromosome, 21 chromosome, 22 chromosome and Y chromosome.

### 3.3 Validation of deregulated mRNAs and lncRNAs

**FIGURE 4:**
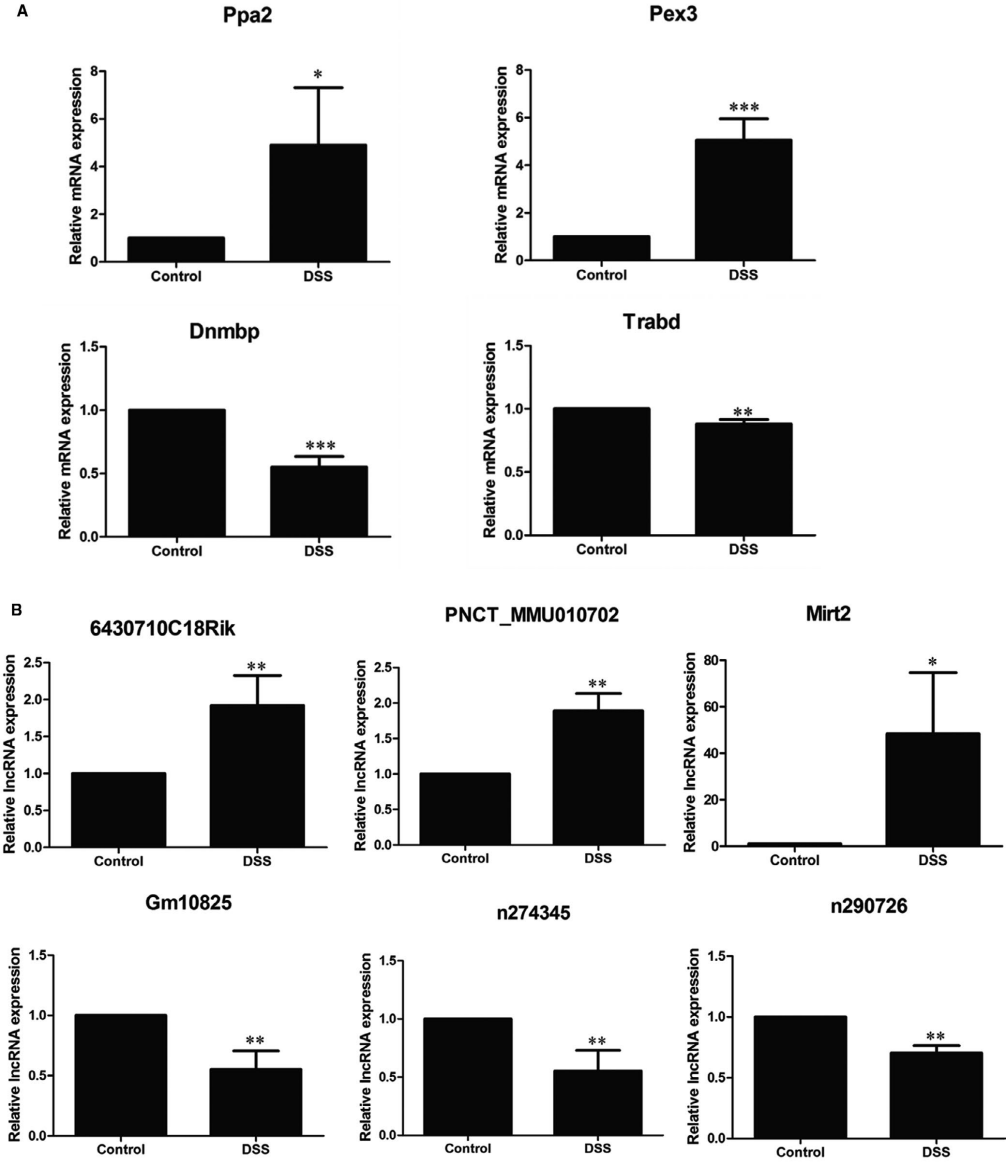
The qRT‐PCR validations of four mRNAs and six lncRNAs. A, The qRT‐PCR results of four mRNAs, Ppa2, Pex3, Dnmbp and Trabd. B, The qRT‐PCR results of six lncRNAs, 6430710C18Rik, PNCT_MMU010702, Mirt2, Gm10825, n274345 and n290726. The data are presented as the mean ± SD (n = 5). *P < .05, **P < .01 and ***P < .001

We randomly selected 4 mRNAs and 6 lncRNAs from the DE mRNAs and DE lncRNAs. The qRT‐PCR analysis was performed to determine the expression level of these lncRNAs and mRNAs obtained from the IECs of colon tissues of Control group (n = 5) and DSS group (n = 5) (Figure 4). Ppa2 and Pex3 mRNAs were up‐regulated, whereas Dnmbp and Trabd were down‐regulated. LncRNAs 6430710C18Rik (ENSMUST00000125382), PNCT_MMU010702 (NONMMUT020382.2) and Mirt2 (ENSMUST00000175179) were up‐regulated, whereas Gm10825 (ENSMUST00000180537), n274345 (NONMMUT033090.2) and n290726 (NONMMUT026732.2) were down‐regulated. The results of qRT‐PCR analysis were consistent with our high‐throughput sequencing findings.

### 3.4 GO annotation and KEGG pathway analysis of DE mRNAs

**FIGURE 5:**
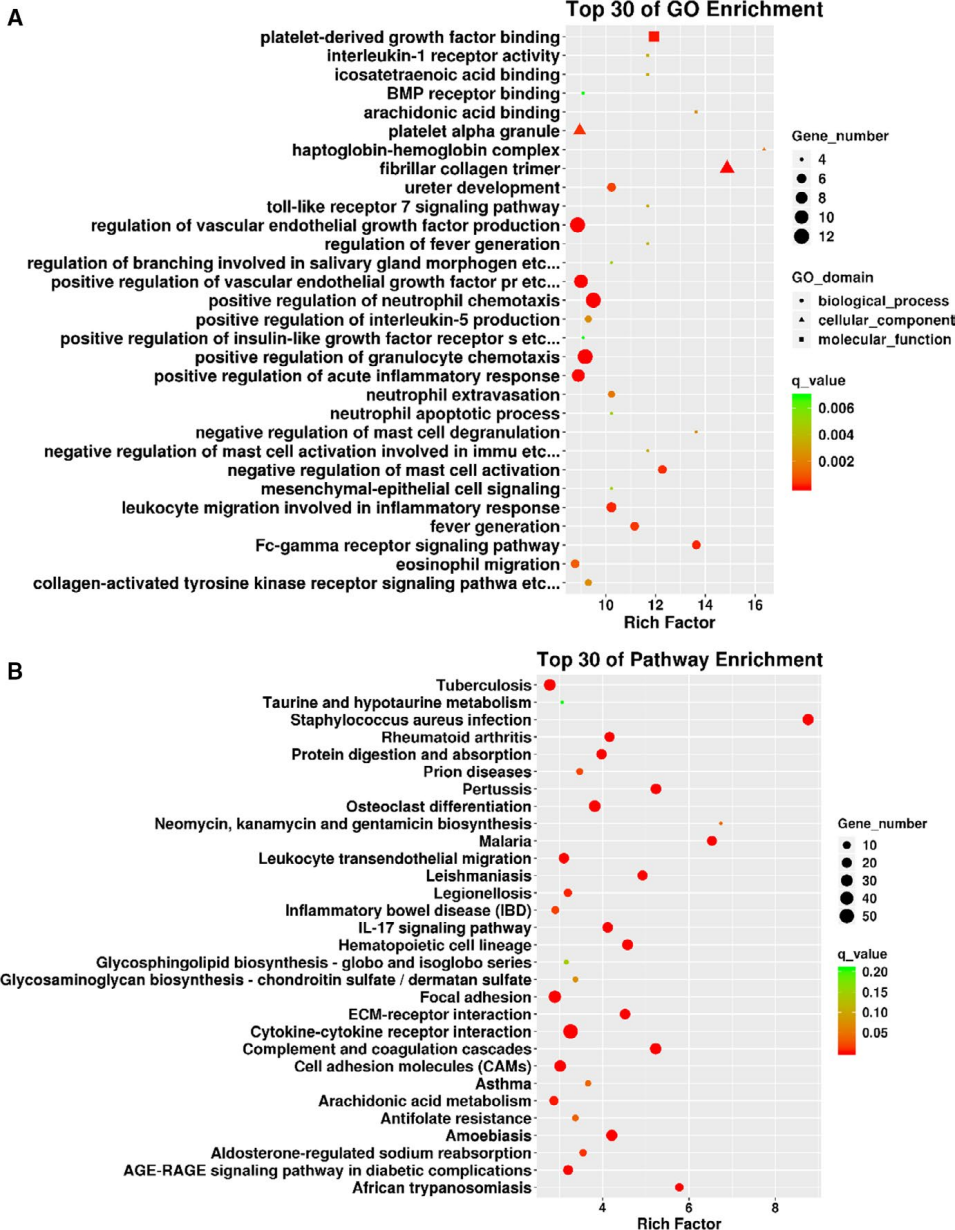
GO and KEGG signalling pathway analysis of DE mRNAs. A, TOP 30 of GO enrichment. The circles represent biological process, the triangles represent cellular component, and the squares represent molecular function. B, TOP 30 of KEGG signalling pathway enrichment

GO analysis and KEGG pathway analysis were performed on significantly dysregulated mRNAs in the DSS group and the Control group. As shown in Figure 5A, the top 30 enriched GO terms of biological process, cellular component and molecular function were identified, including Fc‐gamma receptor signalling pathway, haptoglobin‐haemoglobin complex, toll‐like receptor 7 (TLR7) signalling pathway, fibrillar collagen trimer, arachidonic acid binding, platelet‐derived growth factor binding, eicosatetraenoic acid binding, interleukin (IL)‐1 receptor activity, bone morphogenic protein (BMP) receptor binding and so on.

Furthermore, the KEGG pathway analysis was performed to show that the DE mRNAs were enriched in protein digestion and absorption, IL‐17 signalling pathway, ECM‐receptor interaction, cytokine‐cytokine receptor interaction, cell adhesion molecules (CAMs), inflammatory bowel disease (IBD), leucocyte transendothelial migration, focal adhesion and so on (Figure 5B).

### 3.5 Construction of the ceRNA network

**FIGURE 6:**
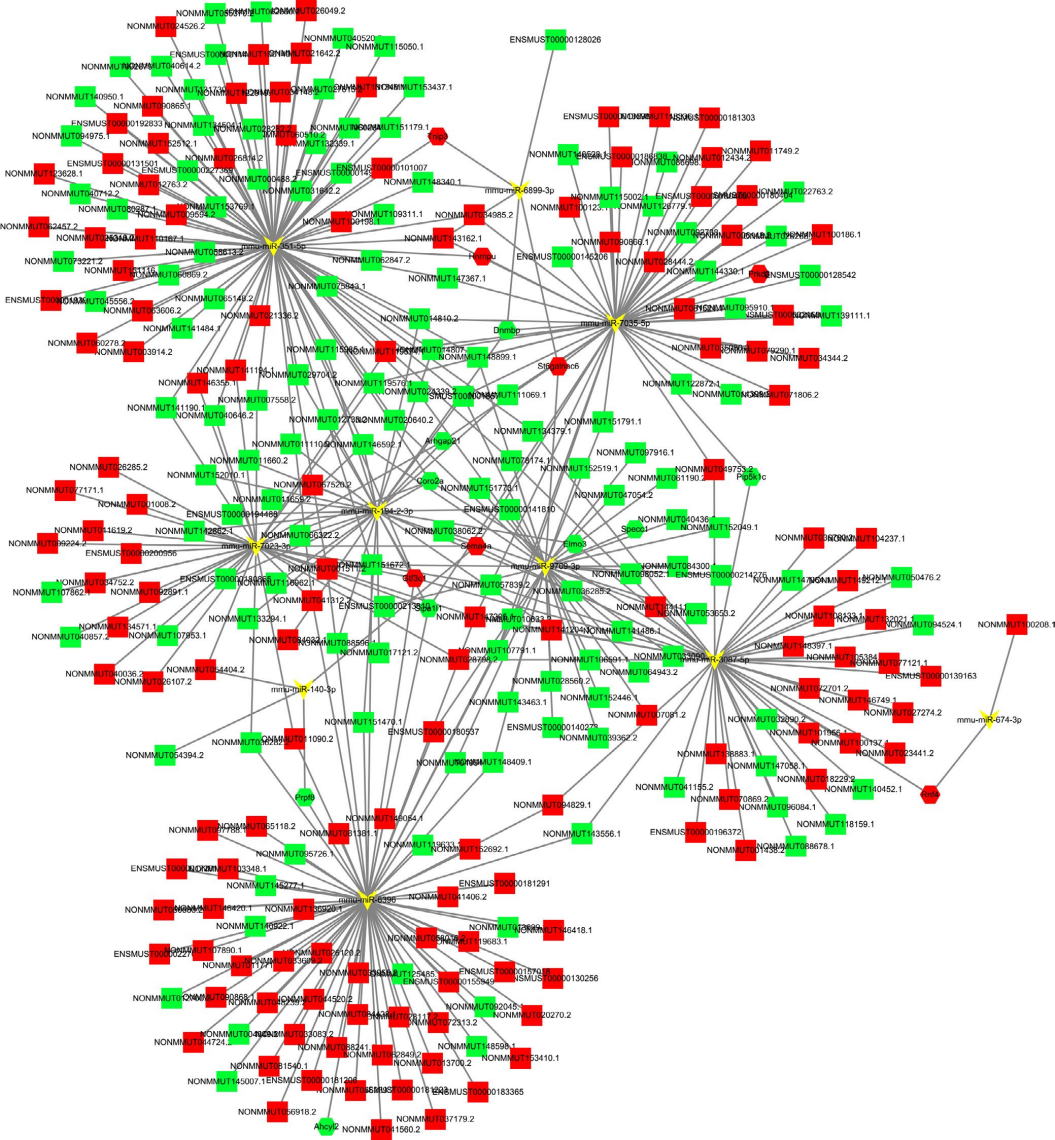
The ceRNA network construction. In the network, red represents up‐regulation and green represents down‐regulation. LncRNAs, miRNAs and mRNAs are presented as quadrilateral, arrowheads and hexagons, respectively. And grey edges indicate lncRNA‐miRNA‐mRNA interactions

With a threshold of *P*‐value <.05 and |Log2 FC| > 8, 20 mRNAs that would be further studied were selected to construct the ceRNA network. The interactions of lncRNA‐miRNA‐mRNA were calculated by miRanda. Cytoscape software was used for network import and visualization. The selected mRNAs were listed in Table S3. And the ceRNA network map for top 500 lncRNA‐miRNA‐mRNA was showed in Figure 6. Among the ceRNA network, there were 16 mRNAs, 280 lncRNAs and 10 miRNAs. The possibility of the indirect interactions between DE lncRNAs and DE mRNAs was illustrated by the ceRNA network.

For instance, Dnmbp was predicted to interact with 3 miRNAs, including mmu‐miR‐7035‐5p, mmu‐miR‐194‐2‐3p and mmu‐miR‐6899‐3p. LncRNA ENSMUST00000128026 was predicted to act as a ceRNA and compete for binding to mmu‐miR‐6899‐3p, thereby regulating the expression of Dnmbp. Tnip3 was predicted to interact with mmu‐miR‐351‐5p and mmu‐miR‐6899‐3p. LncRNA NONMMUT143162.1 was predicted to competitively bind to mmu‐miR‐6899‐3p, regulating Tnip3 expression.

### 3.6 The protein‐protein interaction (PPI) network construction

Protein‐protein interaction networks were constructed according to the information of the STRING database. The PPI networks analysis included 311 nodes and 563 edges. The highest connectivity degrees were Mmp9 (matrix metallopeptidase 9, degree = 27), Fpr2 (formyl peptide receptor 2, degree = 17) and Ccl3 (C‐C motif chemokine ligand 3, degree = 17). The PPI networks of the top 20 nodes were showed in Figure S2. In addition, the top 20 core genes and their corresponding degree were shown in Table S4.

**FIGURE 7:**
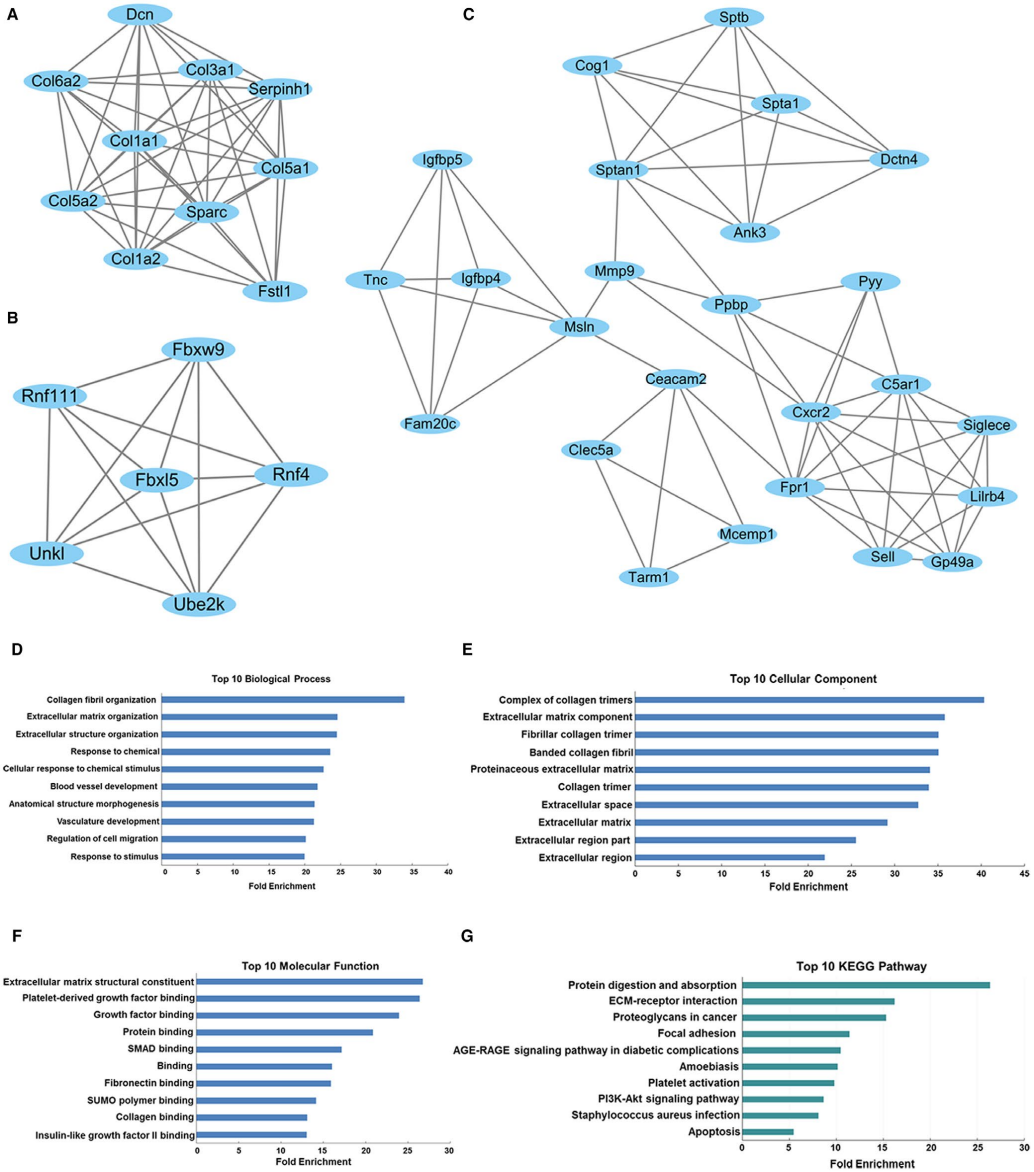
Functional annotation of top three modules in the PPI network. A, Module 1, B, Module 2, C, Module 3. GO enrichment analysis (D‐F) and KEGG pathway analysis (G) were performed for DE mRNAs in the modules

Moreover, MCODE was used to screen the modules of PPI networks (Figure 7A‐C). Then, GO analysis and KEGG pathway analysis were performed to analyse the functional annotation of the genes involved in the top three significant modules (Figure 7D‐G). As shown in Figure 7, the GO enrichment analysis and KEGG pathway analysis of the modules were involved in collagen fibril organization, extracellular structure organization, extracellular matrix organization, complex of collagen trimers, extracellular matrix component, fibrillar collagen trimer, extracellular matrix structural constituent, platelet‐derived growth factor binding, growth factor binding, protein digestion and absorption, ECM‐receptor interaction, proteoglycans in cancer and so on.

## 4 DISCUSSION

The intestinal epithelial barrier, which is primarily formed by a single‐cell layer of IECs, is essential in the maintenance of intestinal homoeostasis. Integrated IECs and TJs between IECs are the main components of the barrier.^38^ Both the increased level of IEC death and the disruption of TJ contributed to the pathogenesis and development of IBD. Recently, the importance of lncRNAs in IBD has become increasingly obvious. RNA‐seq analysis or microarray technology was performed to identify the dysregulated mRNAs and lncRNAs using the whole colon RNA isolated from the mouse models of colitis or IBD patients.^28^, ^30^ However, due to the diversity of cell types in the whole colon, a gene may be significantly changed in one cell type, but in other cell types, the differential effects can be attenuated or eliminated completely. Thus, it is necessary to isolate and purify different cell types and conduct genetic analysis on them. In the present study, focusing on the role of IECs in the pathogenesis and development of IBD, we investigated the mRNAs and lncRNAs expression profile for IECs which were isolated from the colons of wild‐type mice and DSS‐induced mice.

The RNA‐seq analysis exhibited that 1267 mRNAs and 1308 lncRNAs were significantly differentially expressed in the DSS group compared with the Control group (Figures 1 and 2). We selected 6 lncRNAs and 4 mRNAs for validation purposes (Figure 4). The results of qRT‐PCR analysis suggested that up‐regulated and down‐regulated lncRNAs and mRNAs were consistent with the data of RNA‐seq analysis. A previous study, using whole colon sections for RNA‐seq, reported 12 mRNAs (11 up‐regulated and 1 down‐regulated) that are differentially expressed in dextran sodium sulphate (DSS) and interleukin‐10‐deficient mice as well as in IBD patients.^30^ Compared with these 12 mRNAs, 7 up‐regulated mRNAs match with our data: Mmp3, Hcls1, Il1b, Lcn2, Plek, Steap4 and Ubd.

Subsequently, the GO analysis was performed to identify the biological function of DE mRNAs (Figure 5A). In the present study, the enriched GO terms were significantly associated with TLR7 signalling pathway, IL‐1 receptor activity, BMP receptor binding and so on. In addition to forming a physical barrier against foreign antigens, IECs also participate in the innate immune response since IECs express pattern recognition receptors, such as TLRs. TLR7, a member of TLRs family, is known as an important regulator of innate immunity. A previous study has reported that TLR7 agonists Imiquimod could ameliorate DSS‐induced acute colitis.^39^ IL‐1 cytokines are key mediators in immune regulation and inflammatory processes. For example, excess IL‐1β expression was found in CD and other inflammatory conditions of the gut.^40^, ^41^ In addition, IL‐1β has been shown to cause a functional opening of intestinal TJ barrier.^42^ Al‐Sadi et al^43^ demonstrated that the IL‐1β‐induced increase in TJ permeability was mediated by an increase in MLCK expression and activity. It is known that not only the ratio between the proliferation and cell death, but also the balance between the proliferating progenitor cells and differentiating IECs, contributed to the maintenance of intestinal epithelial homoeostasis.^6^ BMPs, which belong to the transforming growth factor‐β superfamily, were known to regulate the epithelial proliferation and differentiation.^44^ Conditional inactivation of the type I BMP receptor Bmpr1a in mice resulted in disturbed homoeostasis of intestinal epithelial regeneration.^45^

The KEGG signalling pathway analysis showed that the most significant pathways were involved in IL‐17 signalling pathway, inflammatory bowel disease (IBD), focal adhesion, cytokine‐cytokine receptor interaction and so on (Figure 5B). IL­‐17 cytokines were thought to induce the mucosal inflammation but also be involved in the restitution and repair of the intestinal mucosa after resolution of inflammation.^46^ Increased expression of IL‐­17 family members was found in both human IBD and animal models of colitis.^47^, ^48^, ^49^, ^50^ Some results suggested the disease‐protective role for IL‐17A in intestinal pathology. For example, Ogawa et al^51^ reported that the neutralization of IL‐17A aggravates DSS‐induced colitis in mice. Yang et al^52^ found that IL‐17A^−/−^ mice displayed more severe intestinal inflammation following DSS treatment. Furthermore, IL‐17A could stimulate the expression of claudins in IECs, thereby mediating the formation of TJ and enhancing the barrier function of IECs.^53^ In contrast, IL‐17F deficiency resulted in reduced colitis caused by DSS, suggesting that IL‐17F may exacerbate the intestinal inflammation.^52^ Overall, the results of GO enrichment analysis and KEGG signalling pathway analysis can help to further explore the mechanism of intestinal barrier regulation in IBD.

Recent research has demonstrated that lncRNAs can act as miRNA sponges to protect mRNAs from miRNA inhibition.^54^, ^55^ Then, we constructed a ceRNA network to better understand the function of lncRNAs (Figure 6). For example, Dnmbp, also known as Tuba, is a Cdc42‐specific guanine‐nucleotide‐exchange factor. In IECs, Dnmbp can control the shaping of cell junctions through the local activation of Cdc42 and its effectors, suggesting that Dnmbp may contribute to the function of intestinal epithelial barrier.^56^, ^57^ Interestingly, our qRT‐PCR analysis results showed the decrease of Dnmbp in the DSS group. According to the ceRNAs network, lncRNA ENSMUST00000128026 was predicted to bind to mmu‐miR‐6899‐3p and further regulate Dnmbp expression. Therefore, lncRNA ENSMUST00000128026 may regulate the intestinal epithelial barrier function through affecting the Dnmbp expression. However, further research is needed to elucidate the function of ENSMUST00000128026 in IBD.

Tnip3 (TNFAIP3 interacting protein 3), also known as ABIN‐3, can negatively regulate the nuclear factor‐κB (NF‐κB) activation in response to tumour necrosis factor (TNF) and lipopolysaccharide (LPS).^58^ In addition to the pro‐inflammatory and anti‐inflammatory effects, NF‐kB can also mediate wound healing in IECs during the inflammatory processes. As a negative regulator of NF‐κB activation, Tnip3 may contribute to the control of wound healing in IECs. It has been reported that Tnip3 expression is significantly up‐regulated in human masticatory mucosa during wound healing.^59^ To date, the role of Tnip3 in IBD is still unknown. Recently, a study showed that Tnip3 was one of the top 10 down‐regulated genes when comparing between the long‐duration and short‐duration UC patients.^60^ Interestingly, in the present study, we found that Tnip3 was predicted to be up‐regulated when comparing between the DSS and control mice. These findings suggest that Tnip3 may play an important role in IBD. However, the association between Tnip3 and IBD remains to be elucidated. Furthermore, the ceRNAs network showed that lncRNA NONMMUT143162.1 was predicted to regulate Tnip3 expression by competitively binding to mmu‐miR‐6899‐3p. Therefore, we will further illuminate the concrete mechanisms in future studies to understand the relationship between this novel RNA crosstalk and IBD.

The PPI network showed that Mmp9, Fpr2, Ccl3, Col1a2, Col1a1, Cxcr2, Sptan1, Ptgs2, C5ar1, Col3a1, Sparc, Fpr1, Mmp8, Clec4d, Lyz2, Lilrb4, Csf1r, Col5a2, Ppbp and Plek were the top 20 high‐degree hub nodes (Table S4). Matrix metalloproteinases (MMPs) are a group of zinc‐dependent endopeptidases, which are involved in the tissue remodelling and degradation of extracellular matrix (ECM).^61^ Increasing evidence suggests that MMPs may play a central role in the pathogenesis of intestinal tissue injury and inflammation in IBD. Koelink et al had reported that MMP8 and MMP9 levels were elevated both in the intestine of patients with IBD and the intestine from DSS‐treated mice.^62^ Liu et al^63^ found that constitutive expression of MMP9 in intestinal epithelium worsens murine acute colitis. Moreover, recent evidence demonstrates that MMP9 induced increase in intestinal epithelial TJ permeability contributes to the severity of experimental DSS colitis.^64^

Fpr2 and Fpr1 is the member of the formyl peptide receptors (FPRs), which mediate the chemotaxis activity. It is known that Fpr signalling plays a key role in gastrointestinal homoeostasis and inflammation. A previous study showed that the endogenous FPR ligand, annexin A1, could regulate the intestinal mucosal injury, inflammation and repair through stimulation of FPRL‐1 in DSS‐induced colitis.^65^ Furthermore, a new endogenous chemotaxis agonist family with sequence similarity 3 member D (FAM3D) has also been reported to play a role in gastrointestinal homoeostasis and inflammation through its receptors FPR1 and FPR2.^66^

In conclusion, this study illuminated the expression profiles of mRNAs and lncRNAs involved in the regulation of the intestinal epithelial barrier in the DSS‐induced colitis. We identified mRNAs and lncRNAs with differential expression between the DSS group and the Control group, and elucidated the characteristics of DE lncRNAs and functions of DE mRNAs. Besides, we predicted several lncRNAs that may contribute to the maintenance of intestinal barrier function. Our findings may provide new insights into the molecular mechanisms underlying the development of IBD. Further research is required to investigate the functions of lncRNA and mRNA identified in the present study.

## CONFLICT OF INTEREST

The authors declare that they have no conflict of interest.

## AUTHOR CONTRIBUTIONS

**Huan Liu:** Data curation (equal); Investigation (lead); Methodology (equal); Validation (equal); Writing‐original draft (equal); Writing‐review & editing (equal). **Teming Li:** Methodology (supporting); Writing‐review & editing (supporting). **Shizhen Zhong:** Project administration (supporting); Writing‐review & editing (supporting). **Min Yu:** Conceptualization (equal); Methodology (equal); Project administration (equal); Resources (equal); Writing‐original draft (equal); Writing‐review & editing (equal). **Wenhua Huang:** Conceptualization (equal); Data curation (equal); Project administration (equal); Resources (equal); Supervision (equal).

## Supporting information

Fig S1‐S2

Table S1‐S4

## Data Availability

The data that support the findings in the current study are available from the corresponding author upon reasonable request.
