# Supplementary material for: Intestinal epithelial cells related lncRNA and mRNA expression profiles in dextran sulphate sodium‐induced colitis
Source: J Cell Mol Med. 2020 Dec 9;25(2):1060–73. doi: 10.1111/jcmm.16174 (PMC7812259; doi:10.1111/jcmm.16174)
Supplement: Supplementary file 1 — Fig S1‐S2 [file JCMM-25-1060-s001.docx]

**Supplementary Figures**


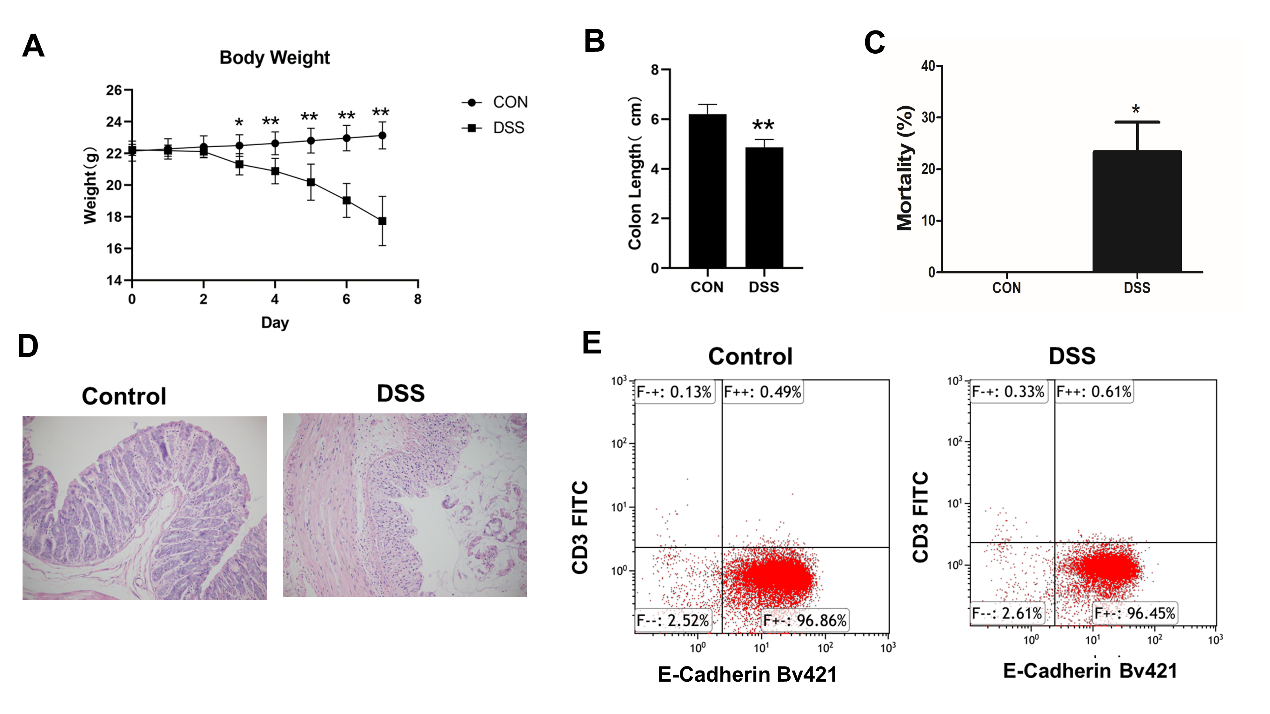


**Figure S1 Dextran sulfate sodium (DSS) induced colitis model. A.** Percent weight change for DSS compared with control. **B.** The colon lengths of Control group and DSS group at day 7. **C.** The percentages of mortality at day 7. **D.** H&E-stained colonic sections of Control group and DSS group. Magnification: ×200. **E.** The purity of the intestinal epithelial cells (IECs). Cells were stained with anti-CD3 FITC and anti-E-cadherin Bv421.


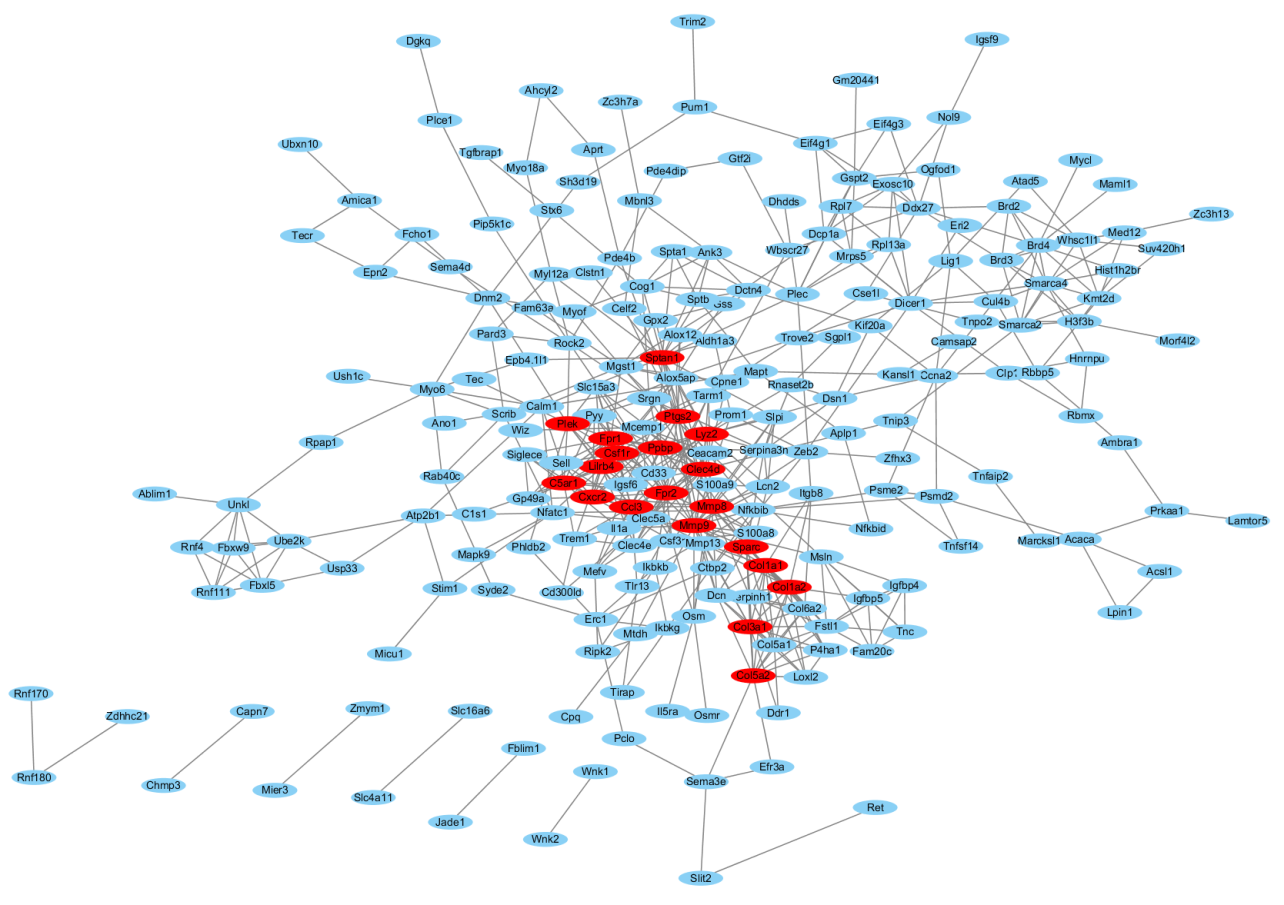


**Figure S2** The PPI network of top 20 high-degree hub nodes. In the network, red represents top 20 high-degree genes, and gray edges indicate protein−protein interactions.
