# Supplementary material for: Intestinal epithelial cells related lncRNA and mRNA expression profiles in dextran sulphate sodium‐induced colitis
Source: J Cell Mol Med. 2020 Dec 9;25(2):1060–73. doi: 10.1111/jcmm.16174 (PMC7812259; doi:10.1111/jcmm.16174)
Supplement: Supplementary file 2 — Table S1‐S4 [file JCMM-25-1060-s002.docx]

**TABLE S1** The primers used for qRT-PCR validation

| Gene name |  | Primer sequence(5'-3') | Product length |
| --- | --- | --- | --- |
| 6430710C18Rik | Forward | GAGGTGTGACAACAGGAGCCTTC | 151 |
|  | Reverse | TGGAGGATGGAGGTCTTGGTGAC |  |
| PNCT_MMU010702 | Forward | GACATCAGGGCGGATGGATTGC | 102 |
|  | Reverse | CATGGTGACTTGTGGGCTGAGC |  |
| Mirt2 | Forward | CACTGGCTCTGTGGTCATCTTCTC | 120 |
|  | Reverse | CCTCTTCCTCAGCTCCACTCTACC |  |
| Gm10825 | Forward | AGGCTCTTACCGCTGGCTCTG | 128 |
|  | Reverse | CCATTCGCAGGAACCTGAACACC |  |
| n274345 | Forward | TTCTCTGCTGCTTGCTGCCTTG | 113 |
|  | Reverse | ACTCCACCTGCCCTGCTCTTC |  |
| n290726 | Forward | GGCTCGCAACACTCAGATGGAG | 143 |
|  | Reverse | TGTCCCAGGATCAGAGGCACAG |  |
| Ppa2 | Forward | ACCGCCTGTTCTTCAAGCATGTAG | 197 |
|  | Reverse | TGGCTCCTCTGTGGCAATCTCC |  |
| Pex3 | Forward | ACAGAGGCTTCAGTCGGCTTCTAG | 110 |
|  | Reverse | AGGCAGGCTGACACTGGACAG |  |
| Dnmbp | Forward | GTTTTGTGGAGATCGTGACAAT | 93 |
|  | Reverse | GACTGTTCAGCTCTCGAGATAC |  |
| Trabd | Forward | ACCGTCCATCTCAGGCAGAGTG | 118 |
|  | Reverse | AGGCAGTGACAGGACCAGGTTC |  |
| Gapdh | Forward | CTCGTCCCGTAGACAAAATGGT | 131 |
|  | Reverse | GAGGTCAATGAAGGGGTCGTT |  |

**TABLE S2** The dysregulated lncRNAs

| lncRNA_id | locus | length | log2FC | Pvalue | Qvalue | updown |
| --- | --- | --- | --- | --- | --- | --- |
| NONMMUT043279.2 | 3:60877817-60878845 | 1029 | 6.93927768 | 1.0074E-22 | 3.6584E-18 | UP |
| NONMMUT011658.2 | 11:95763838-95768186 | 4349 | 5.64210535 | 1.0473E-18 | 1.9016E-14 | UP |
| NONMMUT020382.2 | 14:37102211-37109719 | 592 | 7.12591812 | 3.3917E-18 | 4.1055E-14 | UP |
| NONMMUT019428.2 | 13:115088359-115092939 | 3977 | Inf | 6.2732E-13 | 5.6951E-09 | UP |
| NONMMUT068239.2 | 9:20497646-20498812 | 1167 | -3.834674 | 7.9408E-10 | 5.7673E-06 | DOWN |
| NONMMUT134504.1 | 9:61772700-61826482 | 1382 | #NAME? | 2.5524E-09 | 1.5448E-05 | DOWN |
| NONMMUT032890.2 | 18:69395533-69398226 | 2689 | #NAME? | 1.3371E-08 | 6.9364E-05 | DOWN |
| NONMMUT050920.2 | 4:154918396-154920976 | 2531 | Inf | 1.7383E-07 | 0.00078907 | UP |
| NONMMUT002212.2 | 1:105846126-105847558 | 1433 | 7.54386696 | 2.2117E-07 | 0.00089239 | UP |
| NONMMUT020278.2 | 14:32115643-32117499 | 1857 | 3.58632781 | 3.5815E-07 | 0.00130057 | UP |
| ENSMUST00000200707 | 5:91257571-91283421 | 3201 | #NAME? | 7.6144E-07 | 0.00251373 | DOWN |
| NONMMUT000384.2 | 1:25015304-25018695 | 3242 | Inf | 8.5036E-07 | 0.00257334 | UP |
| NONMMUT026732.2 | 16:44225566-44227463 | 1898 | -5.6715897 | 1.0367E-06 | 0.00268901 | DOWN |
| NONMMUT111069.1 | 3:51559758-51595797 | 3794 | -3.9363297 | 1.025E-06 | 0.00268901 | DOWN |
| NONMMUT064237.2 | 7:141632166-141633001 | 836 | -5.8218027 | 1.3894E-06 | 0.0033066 | DOWN |
| NONMMUT100121.1 | 16:74980258-74988757 | 8500 | 5.65827837 | 1.4569E-06 | 0.0033066 | UP |
| NONMMUT034148.2 | 19:21574694-21596141 | 16663 | Inf | 2.4082E-06 | 0.00460262 | UP |
| NONMMUT092068.1 | 13:95911410-95912584 | 1175 | -3.1439703 | 2.3126E-06 | 0.00460262 | DOWN |
| NONMMUT100214.1 | 16:75241870-75248241 | 6372 | 2.6504249 | 2.2955E-06 | 0.00460262 | UP |
| NONMMUT030905.2 | 17:84134092-84137573 | 1307 | #NAME? | 2.7122E-06 | 0.00469 | DOWN |
| NONMMUT092674.1 | 14:32118000-32118942 | 943 | 2.96820065 | 2.6373E-06 | 0.00469 | UP |
| NONMMUT100176.1 | 16:75162007-75163661 | 1655 | 3.14667905 | 3.2942E-06 | 0.00543754 | UP |
| NONMMUT027553.2 | 16:87376654-87378943 | 2290 | 4.62811509 | 4.3517E-06 | 0.00687071 | UP |
| NONMMUT090868.1 | 13:15902961-15904554 | 1594 | 5.30005806 | 4.5534E-06 | 0.00688962 | UP |
| NONMMUT006148.2 | 10:71225464-71229120 | 3270 | 6.20123438 | 5.1199E-06 | 0.00715445 | UP |
| NONMMUT016470.2 | 13:16028820-16031354 | 2535 | 6.02966239 | 5.1224E-06 | 0.00715445 | UP |
| NONMMUT026106.2 | 16:28667692-28671046 | 3355 | 2.96127396 | 5.4899E-06 | 0.00738365 | UP |
| NONMMUT032404.2 | 18:54984500-54987674 | 3109 | 3.75043671 | 5.7155E-06 | 0.00741256 | UP |
| ENSMUST00000193524 | 3:50400282-50404283 | 4002 | 4.88245049 | 6.1777E-06 | 0.00746657 | UP |
| NONMMUT026869.2 | 16:49913193-49915008 | 1816 | -4.8471578 | 6.2748E-06 | 0.00746657 | DOWN |
| NONMMUT090867.1 | 13:15901560-15902583 | 1024 | 5.59230941 | 6.3739E-06 | 0.00746657 | UP |
| NONMMUT057205.2 | 6:61175604-61180270 | 1766 | 4.56178007 | 7.1648E-06 | 0.00813069 | UP |
| NONMMUT107791.1 | 2:118570137-118576613 | 6382 | #NAME? | 8.4915E-06 | 0.00927177 | DOWN |
| NONMMUT146355.1 | 2:75785576-75792622 | 4930 | Inf | 8.681E-06 | 0.00927177 | UP |
| NONMMUT021254.2 | 14:63665126-63666106 | 873 | #NAME? | 1.1047E-05 | 0.01088022 | DOWN |
| NONMMUT036282.2 | 2:26053545-26057843 | 4299 | -5.4272653 | 1.072E-05 | 0.01088022 | DOWN |
| NONMMUT046816.2 | 4:41185709-41188660 | 2952 | -2.7638278 | 1.1385E-05 | 0.01088022 | DOWN |
| NONMMUT051511.2 | 5:23852120-23854924 | 1237 | #NAME? | 1.1188E-05 | 0.01088022 | DOWN |
| NONMMUT033618.2 | 19:5801389-5802664 | 966 | Inf | 1.1816E-05 | 0.01100219 | UP |
| NONMMUT150285.1 | 6:28209926-28215778 | 5853 | Inf | 1.2504E-05 | 0.01135134 | UP |
| NONMMUT098052.1 | 16:17054008-17058577 | 4564 | #NAME? | 1.2957E-05 | 0.01147569 | DOWN |
| NONMMUT044350.2 | 3:97738854-97739782 | 929 | 3.94827487 | 1.5017E-05 | 0.01298381 | UP |
| NONMMUT007975.2 | 10:122944692-122945759 | 1068 | 4.8143869 | 1.8008E-05 | 0.01486248 | UP |
| NONMMUT047196.2 | 4:48084281-48086445 | 2165 | 5.02224415 | 1.7732E-05 | 0.01486248 | UP |
| NONMMUT089907.1 | 13:55186264-55187796 | 1533 | 3.37756712 | 1.9158E-05 | 0.01527524 | UP |
| NONMMUT100142.1 | 16:75065666-75072238 | 6573 | 3.75635647 | 1.935E-05 | 0.01527524 | UP |
| NONMMUT033401.2 | 19:3083615-3087407 | 3793 | 3.11448738 | 2.0785E-05 | 0.01605901 | UP |
| NONMMUT063026.2 | 7:102119402-102120356 | 955 | 5.71251362 | 2.1497E-05 | 0.01617659 | UP |
| NONMMUT073617.2 | X:105968087-105969483 | 1397 | -3.9718131 | 2.1828E-05 | 0.01617659 | DOWN |
| ENSMUST00000222580 | 13:52729615-52735608 | 2056 | Inf | 2.3824E-05 | 0.01730286 | UP |
| NONMMUT031190.2 | 17:5891774-5902514 | 446 | -3.7566734 | 2.4703E-05 | 0.01758938 | DOWN |
| NONMMUT132885.1 | 9:78520622-78525856 | 5235 | 5.49929317 | 2.6012E-05 | 0.01816561 | UP |
| ENSMUST00000192674 | 5:32130283-32133168 | 2886 | #NAME? | 2.7218E-05 | 0.01864876 | DOWN |
| NONMMUT030906.2 | 17:84134092-84137573 | 1656 | #NAME? | 2.9426E-05 | 0.01978858 | DOWN |
| NONMMUT037123.2 | 2:45057731-45059961 | 2231 | Inf | 3.3795E-05 | 0.02196362 | UP |
| NONMMUT100193.1 | 16:75209105-75210034 | 930 | 3.87422813 | 3.387E-05 | 0.02196362 | UP |
| ENSMUST00000182578 | 7:92737637-92741468 | 3832 | 3.64852422 | 3.633E-05 | 0.02274639 | UP |
| NONMMUT134501.1 | 9:61662723-61826482 | 1465 | Inf | 3.6247E-05 | 0.02274639 | UP |
| NONMMUT100232.1 | 16:75289819-75298774 | 8054 | 3.72524356 | 3.7834E-05 | 0.02328659 | UP |
| NONMMUT033090.2 | 18:75431873-75433795 | 1923 | -4.1399758 | 3.9341E-05 | 0.02381036 | DOWN |
| NONMMUT100180.1 | 16:75174281-75175373 | 1093 | 2.93145024 | 4.2602E-05 | 0.02536135 | UP |
| NONMMUT100186.1 | 16:75190343-75194350 | 4008 | 2.41672693 | 4.5066E-05 | 0.02639572 | UP |
| NONMMUT043292.2 | 3:62460690-62462217 | 1528 | -4.3066059 | 4.757E-05 | 0.02699165 | DOWN |
| NONMMUT059236.2 | 6:136607823-136610855 | 3033 | 3.54331716 | 4.7484E-05 | 0.02699165 | UP |
| NONMMUT027274.2 | 16:75166705-75170168 | 3464 | 2.80663395 | 4.8852E-05 | 0.02728918 | UP |
| NONMMUT147367.1 | 3:52488276-52495199 | 6807 | #NAME? | 4.9598E-05 | 0.02728918 | DOWN |
| NONMMUT062259.2 | 7:75752590-75754606 | 2017 | -7.5283056 | 5.2977E-05 | 0.02850143 | DOWN |
| NONMMUT149575.1 | 5:91251728-91283076 | 2001 | Inf | 5.3371E-05 | 0.02850143 | UP |
| NONMMUT013629.2 | 12:22866888-22871448 | 3227 | #NAME? | 5.5134E-05 | 0.02901647 | DOWN |
| ENSMUST00000155949 | 2:29245107-29252993 | 4301 | Inf | 5.6506E-05 | 0.02931389 | UP |
| NONMMUT153404.1 | 9:90119897-90121118 | 1222 | 4.64322195 | 5.982E-05 | 0.03059589 | UP |
| NONMMUT024356.2 | 15:82141007-82142200 | 1194 | 2.76207206 | 6.2971E-05 | 0.03105911 | UP |
| NONMMUT071806.2 | X:9435498-9437298 | 1801 | 4.15879515 | 6.2027E-05 | 0.03105911 | UP |
| NONMMUT100185.1 | 16:75189011-75190285 | 1275 | 2.71681441 | 6.3292E-05 | 0.03105911 | UP |
| NONMMUT151791.1 | 7:98682336-98702928 | 20593 | -5.0633527 | 6.5414E-05 | 0.03167267 | DOWN |
| NONMMUT100137.1 | 16:75047276-75055567 | 8292 | 2.73917792 | 6.9941E-05 | 0.03341872 | UP |
| NONMMUT032845.2 | 18:68260187-68263497 | 3311 | 3.46546275 | 7.0961E-05 | 0.03346604 | UP |
| ENSMUST00000141787 | 3:94994410-94996996 | 1877 | #NAME? | 7.4799E-05 | 0.03407575 | DOWN |
| NONMMUT024908.2 | 15:98534520-98543049 | 344 | Inf | 7.7884E-05 | 0.03407575 | UP |
| NONMMUT058610.2 | 6:119570798-119571743 | 946 | -4.7829286 | 7.7195E-05 | 0.03407575 | DOWN |
| NONMMUT085259.1 | 11:57555688-57557135 | 1448 | 4.9660276 | 7.6304E-05 | 0.03407575 | UP |
| NONMMUT094829.1 | 14:100276749-100284054 | 7263 | Inf | 7.4832E-05 | 0.03407575 | UP |
| NONMMUT100128.1 | 16:75006403-75012802 | 6400 | 4.11988537 | 7.5186E-05 | 0.03407575 | UP |
| NONMMUT151672.1 | 7:51841684-51858061 | 16378 | #NAME? | 8.0729E-05 | 0.0348999 | DOWN |
| NONMMUT100215.1 | 16:75248314-75253200 | 4887 | 2.14820759 | 8.3791E-05 | 0.03538105 | UP |
| NONMMUT146592.1 | 2:167769032-167783664 | 14521 | -4.6167301 | 8.2985E-05 | 0.03538105 | DOWN |
| NONMMUT040712.2 | 2:156071850-156073896 | 1910 | -3.7295529 | 8.7549E-05 | 0.03612797 | DOWN |
| NONMMUT065156.2 | 8:33697247-33698161 | 915 | 6.38982816 | 8.6709E-05 | 0.03612797 | UP |
| NONMMUT100224.1 | 16:75268491-75270833 | 2343 | 2.22419894 | 8.9068E-05 | 0.03634175 | UP |
| NONMMUT100123.1 | 16:74991099-74998673 | 7575 | 4.29596313 | 9.3541E-05 | 0.03774257 | UP |
| NONMMUT119576.1 | 5:136919147-136937113 | 6327 | #NAME? | 9.8543E-05 | 0.03932418 | DOWN |
| NONMMUT026802.2 | 16:45792570-45794329 | 1760 | Inf | 0.00011042 | 0.04314622 | UP |
| NONMMUT100212.1 | 16:75234501-75241758 | 7258 | 2.10626944 | 0.0001105 | 0.04314622 | UP |
| NONMMUT034843.2 | 19:44394455-44397265 | 2811 | -6.2881718 | 0.00011286 | 0.04353093 | DOWN |
| NONMMUT127959.1 | 7:145217899-145219253 | 1355 | #NAME? | 0.00011508 | 0.04353093 | DOWN |
| NONMMUT147944.1 | 4:10848699-10849011 | 313 | -7.3970148 | 0.0001149 | 0.04353093 | DOWN |
| NONMMUT100226.1 | 16:75273071-75275927 | 2857 | 2.12854996 | 0.00011674 | 0.04370566 | UP |
| NONMMUT020696.2 | 14:49004090-49008563 | 4474 | -2.3029098 | 0.00013398 | 0.04964777 | DOWN |
| ENSMUST00000185789 | 7:143212155-143296549 | 84395 | #NAME? | 0.00013751 | 0.05043971 | DOWN |
| NONMMUT100199.1 | 16:75216574-75217988 | 1415 | 2.2387429 | 0.00013962 | 0.05070213 | UP |
| ENSMUST00000180404 | 3:51560035-51567117 | 3731 | Inf | 0.00014509 | 0.05216607 | UP |
| NONMMUT024380.2 | 15:82642114-82661121 | 869 | #NAME? | 0.00015229 | 0.05346063 | DOWN |
| NONMMUT087550.1 | 12:99645126-99645675 | 550 | -4.3767154 | 0.00015298 | 0.05346063 | DOWN |
| NONMMUT100179.1 | 16:75170984-75172851 | 1868 | 2.91294581 | 0.00015311 | 0.05346063 | UP |
| NONMMUT090866.1 | 13:15899691-15901438 | 1748 | Inf | 0.00018873 | 0.06527314 | UP |
| NONMMUT050350.2 | 4:141625735-141626354 | 620 | -4.4821816 | 0.00020469 | 0.07012313 | DOWN |
| NONMMUT030435.2 | 17:71248007-71249816 | 1810 | -5.3727456 | 0.00021212 | 0.07199151 | DOWN |
| NONMMUT062850.2 | 7:98177194-98184799 | 3181 | -3.4058184 | 0.00022496 | 0.07494764 | DOWN |
| NONMMUT100209.1 | 16:75231649-75233638 | 1990 | 2.15353214 | 0.00022365 | 0.07494764 | UP |
| ENSMUST00000192833 | 17:39843013-39846341 | 3329 | 5.74116338 | 0.0002322 | 0.07622853 | UP |
| NONMMUT048764.2 | 4:114909288-114920831 | 885 | -3.1084178 | 0.00023301 | 0.07622853 | DOWN |
| NONMMUT042946.2 | 3:50442698-50443603 | 906 | 3.57054802 | 0.00024952 | 0.08090362 | UP |
| ENSMUST00000133273 | 15:81415560-81419188 | 580 | Inf | 0.0002558 | 0.08200696 | UP |
| NONMMUT100222.1 | 16:75264551-75265886 | 1336 | 2.47069894 | 0.00025744 | 0.08200696 | UP |
| NONMMUT061190.2 | 7:45569221-45575153 | 2364 | #NAME? | 0.00028095 | 0.08871757 | DOWN |
| NONMMUT052509.2 | 5:65609469-65611884 | 2416 | 2.93499115 | 0.00029273 | 0.09085551 | UP |
| NONMMUT143614.1 | 15:28027438-28030440 | 2183 | Inf | 0.00029064 | 0.09085551 | UP |
| NONMMUT100231.1 | 16:75286715-75289028 | 2314 | 2.0804379 | 0.00031434 | 0.09673624 | UP |
| NONMMUT060075.2 | 7:16915382-16916322 | 941 | -6.241001 | 0.00032082 | 0.0979028 | DOWN |
| NONMMUT100181.1 | 16:75175571-75176596 | 1026 | 2.77242906 | 0.0003375 | 0.10174985 | UP |
| NONMMUT154346.1 | X:105037028-105070124 | 4172 | #NAME? | 0.00033904 | 0.10174985 | DOWN |
| NONMMUT003734.2 | 1:170193433-170196641 | 3209 | 5.26865113 | 0.00034643 | 0.10311755 | UP |
| NONMMUT012873.2 | 11:116874207-116874710 | 504 | 3.47989921 | 0.00034976 | 0.10326252 | UP |
| NONMMUT100223.1 | 16:75266705-75268365 | 1661 | 2.02347279 | 0.00035691 | 0.10452253 | UP |
| ENSMUST00000181605 | 17:23749236-23754065 | 3162 | 4.58302722 | 0.00036948 | 0.10634828 | UP |
| ENSMUST00000222616 | 12:99643093-99647221 | 1328 | Inf | 0.00038364 | 0.10634828 | UP |
| NONMMUT021039.2 | 14:56696275-56701000 | 2103 | 3.96133038 | 0.0003753 | 0.10634828 | UP |
| NONMMUT044724.2 | 3:107733059-107737951 | 4893 | 4.74488955 | 0.0003701 | 0.10634828 | UP |
| NONMMUT047498.2 | 4:59472251-59475576 | 3326 | 4.32906034 | 0.00037756 | 0.10634828 | UP |
| NONMMUT047575.2 | 4:62389055-62389520 | 466 | 5.06056298 | 0.00038127 | 0.10634828 | UP |
| NONMMUT141051.1 | 11:86576084-86583349 | 4561 | #NAME? | 0.00038041 | 0.10634828 | DOWN |
| NONMMUT067828.2 | 8:128732578-128733195 | 618 | 5.7476386 | 0.00039611 | 0.10815321 | UP |
| NONMMUT100183.1 | 16:75184085-75188832 | 4748 | 2.12535364 | 0.00039412 | 0.10815321 | UP |
| NONMMUT049810.2 | 4:131952066-131954119 | 2054 | 3.01761782 | 0.00041449 | 0.11232698 | UP |
| NONMMUT037119.2 | 2:45008428-45010700 | 2273 | 4.77204027 | 0.00042041 | 0.11246796 | UP |
| NONMMUT101955.1 | 17:28387332-28389593 | 2262 | #NAME? | 0.00042222 | 0.11246796 | DOWN |
| NONMMUT145264.1 | 17:84133783-84137410 | 2147 | Inf | 0.0004243 | 0.11246796 | UP |
| ENSMUST00000172812 | 19:5795690-5802672 | 6983 | 4.95324006 | 0.00043418 | 0.1126203 | UP |
| NONMMUT007559.2 | 10:117685499-117687344 | 1846 | -3.3323923 | 0.00043385 | 0.1126203 | DOWN |
| NONMMUT090869.1 | 13:15907540-15908149 | 610 | 4.59564487 | 0.00043163 | 0.1126203 | UP |
| ENSMUST00000139612 | 6:145250529-145251849 | 899 | 3.97472905 | 0.0004838 | 0.12252318 | UP |
| NONMMUT100151.1 | 16:75088739-75093214 | 4476 | 2.49476647 | 0.00048276 | 0.12252318 | UP |
| NONMMUT100198.1 | 16:75215498-75216517 | 1020 | 2.66120203 | 0.00047652 | 0.12252318 | UP |
| NONMMUT100235.1 | 16:75291530-75293890 | 2361 | 1.98674084 | 0.00048585 | 0.12252318 | UP |
| NONMMUT006994.2 | 10:95846246-95853097 | 6737 | -2.9182685 | 0.00049608 | 0.12423816 | DOWN |
| NONMMUT071537.2 | 9:123697012-123699247 | 2236 | 3.51424063 | 0.0005151 | 0.12811875 | UP |
| ENSMUST00000181223 | 10:22306702-22312944 | 4535 | 5.18780678 | 0.00052813 | 0.12822741 | UP |
| ENSMUST00000206358 | 7:75769038-75772046 | 3009 | -5.7220596 | 0.00052966 | 0.12822741 | DOWN |
| NONMMUT026049.2 | 16:24989362-24992572 | 3211 | 2.77973894 | 0.00052444 | 0.12822741 | UP |
| NONMMUT100188.1 | 16:75197864-75202472 | 4603 | 2.91115142 | 0.00052834 | 0.12822741 | UP |
| NONMMUT100201.1 | 16:75219518-75220261 | 744 | 2.26611409 | 0.00053498 | 0.12865623 | UP |
| NONMMUT131348.1 | 8:88661603-88664230 | 1396 | Inf | 0.00054514 | 0.12938684 | UP |
| NONMMUT137219.1 | X:103549501-103623755 | 8696 | -3.4572605 | 0.00054349 | 0.12938684 | DOWN |
| NONMMUT065010.2 | 8:26023360-26026396 | 3037 | 2.91637529 | 0.00055178 | 0.13011178 | UP |
| NONMMUT000488.2 | 1:33792135-33795471 | 3337 | -3.4834509 | 0.00056273 | 0.13183872 | DOWN |
| ENSMUST00000193969 | 1:40088511-40091581 | 3071 | 3.07998869 | 0.00056708 | 0.13200716 | UP |
| ENSMUST00000180685 | 9:122306130-122310928 | 3589 | 5.57480221 | 0.00058654 | 0.13566733 | UP |
| NONMMUT067210.2 | 8:111127444-111145498 | 2548 | Inf | 0.00059142 | 0.13592951 | UP |
| ENSMUST00000194488 | 3:52198248-52200489 | 1934 | -2.5102761 | 0.00059531 | 0.13596386 | DOWN |
| ENSMUST00000163795 | 5:53047687-53049188 | 860 | #NAME? | 0.00060413 | 0.13711492 | DOWN |
| NONMMUT100225.1 | 16:75271796-75276548 | 4747 | 2.64200406 | 0.00061641 | 0.13903322 | UP |
| NONMMUT053467.2 | 5:104636254-104645041 | 7538 | Inf | 0.00062828 | 0.13997118 | UP |
| NONMMUT100189.1 | 16:75202592-75205569 | 2978 | 2.32306393 | 0.00062713 | 0.13997118 | UP |
| NONMMUT100208.1 | 16:75228847-75231578 | 2732 | 1.96390386 | 0.00063341 | 0.14025457 | UP |
| NONMMUT018123.2 | 13:67658735-67664184 | 5065 | #NAME? | 0.00064518 | 0.14199542 | DOWN |
| ENSMUST00000125382 | 2:72746505-72750853 | 511 | Inf | 0.00065478 | 0.14238177 | UP |
| NONMMUT100167.1 | 16:75138782-75140041 | 1260 | 2.64978707 | 0.00065307 | 0.14238177 | UP |
| NONMMUT035148.2 | 19:55931769-55933227 | 1459 | -4.8413741 | 0.00066239 | 0.14317782 | DOWN |
| NONMMUT017649.2 | 13:55184771-55186647 | 1877 | 2.78151427 | 0.00067093 | 0.14416564 | UP |
| ENSMUST00000180442 | 19:53460631-53462723 | 1485 | -2.8293411 | 0.00067813 | 0.14485728 | DOWN |
| NONMMUT035204.2 | 19:57116776-57118935 | 2137 | -2.6019976 | 0.00068969 | 0.146416 | DOWN |
| NONMMUT036942.2 | 2:37519747-37522301 | 2555 | 2.95235571 | 0.00069349 | 0.146416 | UP |
| NONMMUT100194.1 | 16:75209711-75210487 | 777 | 2.7568681 | 0.00069758 | 0.14642818 | UP |
| NONMMUT025568.2 | 16:13702542-13703187 | 646 | 5.19876008 | 0.00070931 | 0.14718815 | UP |
| NONMMUT100690.1 | 17:17190750-17191833 | 1084 | #NAME? | 0.00070861 | 0.14718815 | DOWN |
| ENSMUST00000121317 | 11:83695275-83696185 | 217 | Inf | 0.00072145 | 0.14736913 | UP |
| MSTRG.18275.3 | 19:40508428-40508853 | 316 | -4.2443144 | 0.00071839 | 0.14736913 | DOWN |
| NONMMUT133034.1 | 9:89262151-89263572 | 1422 | 3.61618499 | 0.00072236 | 0.14736913 | UP |
| NONMMUT030534.2 | 17:74055420-74080280 | 1734 | Inf | 0.00073197 | 0.14849487 | UP |
| MSTRG.24566.1 | 4:61868046-61886604 | 516 | 2.2972318 | 0.00074431 | 0.15010023 | UP |
| NONMMUT154345.1 | X:105037028-105070124 | 4343 | Inf | 0.00074815 | 0.15010023 | UP |
| NONMMUT025018.2 | 15:100013568-100015933 | 2366 | 4.66283736 | 0.0007612 | 0.15187947 | UP |
| NONMMUT036579.2 | 2:30447129-30447804 | 676 | 5.21263783 | 0.00077775 | 0.15420639 | UP |
| NONMMUT100140.1 | 16:75060176-75065665 | 5490 | 2.74799094 | 0.0007856 | 0.15420639 | UP |
| NONMMUT100221.1 | 16:75261601-75263060 | 1460 | 1.97103379 | 0.00078312 | 0.15420639 | UP |
| ENSMUST00000197001 | 5:43784046-43786117 | 2072 | 3.66019316 | 0.00080956 | 0.15805613 | UP |
| NONMMUT100210.1 | 16:75233702-75234433 | 732 | 2.59362522 | 0.00081456 | 0.15818068 | UP |
| NONMMUT100216.1 | 16:75254166-75255080 | 915 | 2.10830014 | 0.00083689 | 0.16165353 | UP |
| NONMMUT001028.2 | 1:58390938-58393329 | 1949 | Inf | 0.00085593 | 0.1644567 | UP |
| NONMMUT100230.1 | 16:75284442-75285493 | 1052 | 2.14253556 | 0.00086642 | 0.16559473 | UP |
| NONMMUT044596.2 | 3:104453984-104456749 | 2766 | -1.7851685 | 0.00089035 | 0.16677017 | DOWN |
| NONMMUT046358.2 | 4:19608303-19609983 | 1681 | 2.85727529 | 0.00088299 | 0.16677017 | UP |
| NONMMUT146776.1 | 2:34769388-34771794 | 2407 | #NAME? | 0.00089093 | 0.16677017 | DOWN |
| NONMMUT151773.1 | 7:92728517-92741459 | 11773 | #NAME? | 0.00089072 | 0.16677017 | DOWN |
| NONMMUT144111.1 | 15:83353646-83367312 | 6113 | 4.57196697 | 0.00091214 | 0.16986456 | UP |
| NONMMUT056588.2 | 6:41223514-41226363 | 2850 | 2.51808236 | 0.00095775 | 0.17654708 | UP |
| NONMMUT118751.1 | 5:92971886-92972889 | 1004 | -3.9108341 | 0.00095635 | 0.17654708 | DOWN |
| NONMMUT060889.2 | 7:40190909-40191346 | 438 | #NAME? | 0.00098139 | 0.17999178 | DOWN |
| ENSMUST00000180865 | 9:107558809-107568412 | 5192 | #NAME? | 0.00101085 | 0.18406919 | DOWN |
| NONMMUT084772.1 | 11:5554704-5555574 | 871 | Inf | 0.00101376 | 0.18406919 | UP |
| NONMMUT031642.2 | 18:21001468-21026268 | 1029 | -5.1297554 | 0.00102044 | 0.18436005 | DOWN |
| NONMMUT021336.2 | 14:65919395-65920939 | 1545 | Inf | 0.00103557 | 0.18536394 | UP |
| NONMMUT100163.1 | 16:75120702-75123442 | 2741 | 3.29154021 | 0.00103621 | 0.18536394 | UP |
| ENSMUST00000207697 | 7:98905087-98908055 | 2760 | Inf | 0.00104168 | 0.18542949 | UP |
| NONMMUT132339.1 | 9:40820647-40825972 | 5326 | -2.2826087 | 0.00105222 | 0.18639218 | DOWN |
| NONMMUT153769.1 | 9:61772296-61825862 | 4141 | -2.6688708 | 0.00107601 | 0.18968132 | DOWN |
| ENSMUST00000129876 | 11:29170909-29173558 | 2203 | #NAME? | 0.0010845 | 0.19024873 | DOWN |
| NONMMUT146198.1 | 2:15081948-15082169 | 222 | 5.26301535 | 0.00108971 | 0.19024873 | UP |
| NONMMUT058534.2 | 6:116624922-116626448 | 1527 | -2.7637696 | 0.00109497 | 0.19025279 | DOWN |
| NONMMUT009872.2 | 11:57482999-57483804 | 806 | -3.524014 | 0.00111706 | 0.1931662 | DOWN |
| NONMMUT001133.2 | 1:60336293-60338318 | 2026 | 4.0442183 | 0.00113058 | 0.19457843 | UP |
| ENSMUST00000148598 | 11:62919079-62921592 | 1011 | #NAME? | 0.00114671 | 0.19550039 | DOWN |
| NONMMUT010072.2 | 11:60920812-60931924 | 1449 | 3.02789722 | 0.0011445 | 0.19550039 | UP |
| NONMMUT126810.1 | 7:48752807-48778192 | 3097 | #NAME? | 0.00116665 | 0.19797107 | DOWN |
| NONMMUT125596.1 | 7:92639534-92640352 | 594 | Inf | 0.00118131 | 0.19952649 | UP |
| ENSMUST00000210200 | 2:144168473-144174364 | 1182 | Inf | 0.00122583 | 0.20608665 | UP |
| NONMMUT100160.1 | 16:75111226-75114347 | 3015 | 3.44707669 | 0.00124856 | 0.20894045 | UP |
| NONMMUT095644.1 | 15:36842167-36844354 | 2188 | -5.3681003 | 0.00126897 | 0.2104171 | DOWN |
| NONMMUT100172.1 | 16:75149123-75151214 | 2092 | 2.3919546 | 0.00126655 | 0.2104171 | UP |
| NONMMUT057839.2 | 6:87788866-87791317 | 2452 | -4.8046482 | 0.0013037 | 0.21421942 | DOWN |
| NONMMUT100219.1 | 16:75258828-75260163 | 1336 | 1.95853223 | 0.00130194 | 0.21421942 | UP |
| NONMMUT044449.2 | 3:101040236-101041256 | 1021 | -3.878568 | 0.00131583 | 0.21523863 | DOWN |
| NONMMUT019373.2 | 13:113614213-113614930 | 718 | Inf | 0.00134332 | 0.21875046 | UP |
| NONMMUT027894.2 | 16:96435683-96439465 | 2576 | -3.6629257 | 0.00138763 | 0.22495715 | DOWN |
| NONMMUT109311.1 | 2:84586896-84592838 | 5875 | #NAME? | 0.0014292 | 0.22964642 | DOWN |
| NONMMUT145133.1 | 17:43254625-43258358 | 3734 | 4.11451025 | 0.00142638 | 0.22964642 | UP |
| NONMMUT050369.2 | 4:141760188-141761080 | 893 | 4.76929412 | 0.00144219 | 0.23071198 | UP |
| NONMMUT019731.2 | 14:16432960-16435261 | 2302 | -2.9055624 | 0.00145682 | 0.23203053 | DOWN |
| NONMMUT016546.2 | 13:20605607-20608351 | 2745 | 4.72534814 | 0.00147518 | 0.23366642 | UP |
| NONMMUT026827.2 | 16:46447646-46448689 | 1044 | 4.46763054 | 0.00148639 | 0.23366642 | UP |
| NONMMUT069598.2 | 9:65195902-65196685 | 784 | #NAME? | 0.00148431 | 0.23366642 | DOWN |
| NONMMUT040614.2 | 2:154522813-154526789 | 3511 | -1.9333456 | 0.00150877 | 0.23616164 | DOWN |
| NONMMUT140723.1 | 11:116671660-116675799 | 1094 | #NAME? | 0.00151625 | 0.23631308 | DOWN |
| NONMMUT036553.2 | 2:30151030-30152009 | 980 | -2.096226 | 0.00153126 | 0.23763261 | DOWN |
| NONMMUT039773.2 | 2:128998922-129001325 | 2404 | Inf | 0.00155312 | 0.24000005 | UP |
| NONMMUT072701.2 | X:68821093-68822209 | 1019 | Inf | 0.00156199 | 0.24034755 | UP |
| ENSMUST00000188481 | 17:69417402-69421632 | 4231 | #NAME? | 0.00158301 | 0.24255481 | DOWN |
| NONMMUT081405.1 | 10:9141817-9147580 | 5764 | Inf | 0.00164543 | 0.25105911 | UP |
| NONMMUT005100.2 | 10:26787862-26793323 | 3397 | #NAME? | 0.00170663 | 0.25546542 | DOWN |
| NONMMUT022763.2 | 15:10832091-10835146 | 3056 | -2.1690594 | 0.00170866 | 0.25546542 | DOWN |
| NONMMUT039154.2 | 2:113831549-113832110 | 562 | -4.3853498 | 0.00170948 | 0.25546542 | DOWN |
| NONMMUT050583.2 | 4:148077063-148079507 | 2445 | 3.56173328 | 0.00170331 | 0.25546542 | UP |
| NONMMUT142862.1 | 13:65239445-65242816 | 3134 | #NAME? | 0.00169613 | 0.25546542 | DOWN |
| ENSMUST00000127533 | X:105037197-105070096 | 3164 | -2.7461278 | 0.00173587 | 0.25834648 | DOWN |
| ENSMUST00000128542 | 19:5406874-5422847 | 2106 | #NAME? | 0.00177936 | 0.26062401 | DOWN |
| NONMMUT034702.2 | 19:40741230-40742654 | 1425 | 3.66088839 | 0.00176336 | 0.26062401 | UP |
| NONMMUT048320.2 | 4:100442430-100444494 | 2065 | -3.4279118 | 0.00179029 | 0.26062401 | DOWN |
| NONMMUT100158.1 | 16:75108799-75111114 | 2316 | 3.03110848 | 0.00179337 | 0.26062401 | UP |
| NONMMUT100200.1 | 16:75218538-75219464 | 927 | 2.5111022 | 0.00177802 | 0.26062401 | UP |
| NONMMUT100202.1 | 16:75220331-75221258 | 928 | 1.85659957 | 0.00179424 | 0.26062401 | UP |
| ENSMUST00000224554 | 13:120010390-120018931 | 1509 | 4.6214924 | 0.00183462 | 0.26332995 | UP |
| NONMMUT003162.2 | 1:152540277-152543629 | 3353 | 4.18460585 | 0.0018319 | 0.26332995 | UP |
| NONMMUT093034.1 | 14:59178283-59180680 | 1595 | -2.9038303 | 0.00182326 | 0.26332995 | DOWN |
| NONMMUT146418.1 | 2:119594096-119607721 | 7692 | Inf | 0.00188537 | 0.26954906 | UP |
| NONMMUT051287.2 | 5:14945294-14978541 | 1121 | -2.7079515 | 0.00193001 | 0.27484822 | DOWN |
| NONMMUT100177.1 | 16:75164992-75165724 | 733 | 2.65890577 | 0.00194457 | 0.27583971 | UP |
| ENSMUST00000135987 | 4:55563265-55599296 | 1603 | Inf | 0.00195382 | 0.27607336 | UP |
| NONMMUT068291.2 | 9:21237835-21238817 | 983 | 2.53519804 | 0.00196902 | 0.27714303 | UP |
| NONMMUT100206.1 | 16:75225907-75227155 | 1249 | 2.90992424 | 0.00198865 | 0.27775309 | UP |
| NONMMUT107890.1 | 2:129315634-129423177 | 7625 | 4.78527277 | 0.00198653 | 0.27775309 | UP |
| NONMMUT100192.1 | 16:75206759-75208324 | 1566 | 1.94036364 | 0.00204509 | 0.28454144 | UP |
| NONMMUT039362.2 | 2:119593922-119607721 | 8731 | -1.8512978 | 0.00206823 | 0.28666308 | DOWN |
| NONMMUT026736.2 | 16:44329997-44333050 | 2771 | Inf | 0.00209373 | 0.28799929 | UP |
| NONMMUT100207.1 | 16:75227211-75228569 | 1359 | 2.02102129 | 0.00208744 | 0.28799929 | UP |
| ENSMUST00000179676 | 11:70864660-70872754 | 2138 | Inf | 0.0021023 | 0.28808595 | UP |
| NONMMUT028560.2 | 17:22710467-22721066 | 8472 | -6.4512622 | 0.00216366 | 0.29538019 | DOWN |
| MSTRG.14611.1 | 17:8993800-9031263 | 240 | Inf | 0.00219892 | 0.29907001 | UP |
| NONMMUT065240.2 | 8:35582503-35589020 | 1728 | -2.9214939 | 0.00221584 | 0.30024584 | DOWN |
| NONMMUT132249.1 | 9:32024947-32026328 | 1382 | 3.49854992 | 0.0022304 | 0.30109602 | UP |
| NONMMUT037260.2 | 2:52040405-52041683 | 1279 | Inf | 0.00225416 | 0.30245395 | UP |
| NONMMUT100109.1 | 16:74941072-74944833 | 3762 | Inf | 0.00225712 | 0.30245395 | UP |
| NONMMUT081500.1 | 10:9475733-9476852 | 1120 | Inf | 0.00232033 | 0.30978073 | UP |
| NONMMUT100205.1 | 16:75224596-75225815 | 1220 | 2.01941515 | 0.00235648 | 0.31345444 | UP |
| NONMMUT140950.1 | 11:69057279-69059691 | 2413 | #NAME? | 0.00236737 | 0.31375447 | DOWN |
| NONMMUT026097.2 | 16:28461118-28464374 | 3257 | 2.98856422 | 0.00240883 | 0.31808849 | UP |
| NONMMUT109710.1 | 2:131430771-131434400 | 1056 | #NAME? | 0.00244213 | 0.32131689 | DOWN |
| NONMMUT000353.2 | 1:23366437-23369897 | 3461 | 2.49514247 | 0.00247882 | 0.32496683 | UP |
| NONMMUT139111.1 | 1:91909794-91912223 | 1361 | #NAME? | 0.00251327 | 0.32829781 | DOWN |
| NONMMUT081538.1 | 10:9570303-9575638 | 5336 | 2.9902776 | 0.00252828 | 0.3290754 | UP |
| NONMMUT100162.1 | 16:75115264-75119762 | 4499 | 2.3048325 | 0.00258198 | 0.33364887 | UP |
| NONMMUT100218.1 | 16:75257652-75258743 | 1092 | 2.45331992 | 0.00258512 | 0.33364887 | UP |
| NONMMUT143827.1 | 15:86194453-86195324 | 872 | -1.748005 | 0.00259098 | 0.33364887 | DOWN |
| MSTRG.36585.4 | 9:35078978-35083519 | 763 | Inf | 0.00260477 | 0.3342388 | UP |
| NONMMUT032162.2 | 18:42333299-42341549 | 3368 | 3.15238063 | 0.00265852 | 0.33676857 | UP |
| NONMMUT041312.2 | 2:167769032-167783664 | 9808 | Inf | 0.00267022 | 0.33676857 | UP |
| NONMMUT100195.1 | 16:75210635-75211533 | 899 | 2.01749882 | 0.0026362 | 0.33676857 | UP |
| NONMMUT100228.1 | 16:75280176-75281417 | 1242 | 1.91136163 | 0.00267085 | 0.33676857 | UP |
| NONMMUT130513.1 | 8:35582573-35588973 | 1807 | Inf | 0.00266544 | 0.33676857 | UP |
| NONMMUT081496.1 | 10:9466216-9469378 | 3158 | Inf | 0.00269456 | 0.33858176 | UP |
| NONMMUT001835.2 | 1:86344908-86348036 | 3129 | -3.0853461 | 0.00274623 | 0.34388474 | DOWN |
| NONMMUT026814.2 | 16:45960462-45962858 | 2397 | 2.71646316 | 0.00275665 | 0.34400307 | UP |
| NONMMUT044882.2 | 3:115941363-115942106 | 744 | -3.721753 | 0.00280821 | 0.34865781 | DOWN |
| NONMMUT059031.2 | 6:128409808-128410159 | 352 | -3.4493212 | 0.00281315 | 0.34865781 | DOWN |
| NONMMUT100154.1 | 16:75096835-75102699 | 5859 | 2.06504019 | 0.00284547 | 0.35146416 | UP |
| NONMMUT014399.2 | 12:65052258-65055027 | 2770 | -3.0681666 | 0.00288101 | 0.35464689 | DOWN |
| NONMMUT040716.2 | 2:156091958-156095089 | 3132 | 3.84180655 | 0.00295362 | 0.36235706 | UP |
| ENSMUST00000182943 | X:88759471-88760312 | 842 | #NAME? | 0.00297338 | 0.36236937 | DOWN |
| NONMMUT045556.2 | 3:142546984-142550047 | 3064 | -3.0251365 | 0.00297368 | 0.36236937 | DOWN |
| NONMMUT031804.2 | 18:31635128-31639380 | 3825 | -2.3075742 | 0.00301116 | 0.36327986 | DOWN |
| NONMMUT103472.1 | 18:46787466-46788533 | 523 | 3.54739663 | 0.00300965 | 0.36327986 | UP |
| NONMMUT104237.1 | 18:26004299-26005640 | 1342 | 2.12967542 | 0.00300578 | 0.36327986 | UP |
| ENSMUST00000147174 | 6:52245969-52249795 | 1116 | Inf | 0.00306303 | 0.36442922 | UP |
| NONMMUT011619.2 | 11:94951640-94953040 | 1401 | 3.49735277 | 0.00309093 | 0.36442922 | UP |
| NONMMUT045972.2 | 4:3614876-3616619 | 1744 | -2.3877983 | 0.00304539 | 0.36442922 | DOWN |
| NONMMUT050921.2 | 4:154918396-154920976 | 2564 | -3.511561 | 0.00303599 | 0.36442922 | DOWN |
| NONMMUT054404.2 | 5:123133764-123141203 | 4589 | 4.1824321 | 0.00307403 | 0.36442922 | UP |
| NONMMUT072752.2 | X:71420072-71421793 | 1722 | 3.4142321 | 0.00305163 | 0.36442922 | UP |
| NONMMUT096931.1 | 15:37132683-37135433 | 2751 | Inf | 0.00308813 | 0.36442922 | UP |
| NONMMUT149031.1 | 5:90886231-90889228 | 2998 | Inf | 0.00310245 | 0.3646026 | UP |
| NONMMUT021638.2 | 14:75176865-75182956 | 6068 | #NAME? | 0.00314003 | 0.36782961 | DOWN |
| NONMMUT012812.2 | 11:116285230-116286778 | 1543 | Inf | 0.00322091 | 0.37516265 | UP |
| NONMMUT019730.2 | 14:16430656-16432638 | 1983 | -2.7979614 | 0.0032233 | 0.37516265 | DOWN |
| NONMMUT012511.2 | 11:109011680-109012118 | 439 | -2.2913445 | 0.00324078 | 0.37564631 | DOWN |
| NONMMUT027908.2 | 16:97664674-97666691 | 947 | -2.3121214 | 0.00324814 | 0.37564631 | DOWN |
| NONMMUT033387.2 | 18:89836019-89839510 | 3296 | 2.13424766 | 0.00330701 | 0.38003424 | UP |
| NONMMUT101830.1 | 17:15456723-15458400 | 1678 | -4.2257063 | 0.00330618 | 0.38003424 | DOWN |
| NONMMUT110472.1 | 3:5860338-5860904 | 567 | -1.6548745 | 0.00335704 | 0.38456636 | DOWN |
| NONMMUT100146.1 | 16:75075803-75077293 | 1491 | 4.90972968 | 0.00339873 | 0.38811834 | UP |
| NONMMUT073938.2 | X:135742735-135780786 | 2308 | #NAME? | 0.00344491 | 0.38976696 | DOWN |
| NONMMUT084428.1 | 11:103502222-103515844 | 4269 | 4.33687627 | 0.00344696 | 0.38976696 | UP |
| NONMMUT148706.1 | 4:132269051-132270205 | 1155 | 5.30714179 | 0.00342648 | 0.38976696 | UP |
| NONMMUT148899.1 | 5:28032090-28060425 | 28336 | -1.8397311 | 0.0034561 | 0.38976696 | DOWN |
| NONMMUT100217.1 | 16:75255493-75256620 | 1128 | 1.88254883 | 0.00348765 | 0.39210709 | UP |
| NONMMUT030191.2 | 17:62807573-62810098 | 2526 | Inf | 0.0035128 | 0.39223243 | UP |
| NONMMUT034316.2 | 19:27968127-27971029 | 2903 | Inf | 0.00355318 | 0.39223243 | UP |
| NONMMUT041650.2 | 2:174458492-174459356 | 865 | Inf | 0.00355255 | 0.39223243 | UP |
| NONMMUT057215.2 | 6:62103514-62106233 | 2720 | Inf | 0.00352417 | 0.39223243 | UP |
| NONMMUT100147.1 | 16:75078423-75080207 | 1785 | 3.96401408 | 0.00355357 | 0.39223243 | UP |
| NONMMUT103347.1 | 18:36315038-36324360 | 9211 | -2.6119936 | 0.00350167 | 0.39223243 | DOWN |
| NONMMUT072370.2 | X:50555774-50595613 | 3002 | Inf | 0.00361071 | 0.39733127 | UP |
| ENSMUST00000217076 | 9:42141993-42144024 | 1340 | Inf | 0.00362256 | 0.39743074 | UP |
| NONMMUT080642.1 | 10:89824914-89829151 | 4238 | -2.9946769 | 0.00364732 | 0.3979901 | DOWN |
| NONMMUT090865.1 | 13:15895597-15897194 | 1598 | Inf | 0.00366054 | 0.3979901 | UP |
| NONMMUT099569.1 | 16:28623384-28632154 | 7714 | 1.99269994 | 0.00365342 | 0.3979901 | UP |
| NONMMUT040520.2 | 2:152313189-152313994 | 806 | -3.3279958 | 0.00367766 | 0.39865859 | DOWN |
| NONMMUT010633.2 | 11:73114094-73117242 | 3149 | #NAME? | 0.00372168 | 0.40222962 | DOWN |
| NONMMUT112835.1 | 3:23798107-23804615 | 6509 | 2.03889705 | 0.00373601 | 0.40258039 | UP |
| NONMMUT118172.1 | 5:31559217-31561528 | 2312 | -2.2448526 | 0.00377508 | 0.40558619 | DOWN |
| ENSMUST00000180408 | 8:11477929-11480241 | 1856 | 3.09911493 | 0.00382506 | 0.40974428 | UP |
| NONMMUT004666.2 | 10:9448079-9451438 | 3360 | Inf | 0.00390528 | 0.41710689 | UP |
| NONMMUT011771.2 | 11:96944281-96966805 | 4566 | 2.39214007 | 0.0039523 | 0.42089073 | UP |
| NONMMUT009224.2 | 11:34066248-34068230 | 1983 | 2.23935664 | 0.00400073 | 0.42401286 | UP |
| NONMMUT082862.1 | 10:121274639-121291284 | 991 | 3.30564405 | 0.00400497 | 0.42401286 | UP |
| NONMMUT011660.2 | 11:95769502-95772169 | 2668 | -2.5300586 | 0.00405857 | 0.4257141 | DOWN |
| NONMMUT025031.2 | 15:100260653-100261107 | 455 | 4.50413639 | 0.0040649 | 0.4257141 | UP |
| NONMMUT112855.1 | 3:24323303-24324147 | 845 | 3.44392251 | 0.00406793 | 0.4257141 | UP |
| NONMMUT142509.1 | 13:98317069-98322867 | 3001 | Inf | 0.00404233 | 0.4257141 | UP |
| NONMMUT148168.1 | 4:115813051-115815457 | 294 | Inf | 0.00409127 | 0.42692688 | UP |
| NONMMUT016954.2 | 13:34033063-34037147 | 4085 | -1.7314706 | 0.00418842 | 0.4349395 | DOWN |
| NONMMUT143181.1 | 14:64811959-64825691 | 1672 | 2.75925379 | 0.00419201 | 0.4349395 | UP |
| NONMMUT139409.1 | 10:40258406-40259690 | 1285 | #NAME? | 0.00422959 | 0.43758802 | DOWN |
| NONMMUT069761.2 | 9:67984421-67987014 | 2594 | 2.66734053 | 0.00424224 | 0.43764947 | UP |
| NONMMUT010132.2 | 11:62221203-62223002 | 1800 | -3.1751019 | 0.00425787 | 0.43801764 | DOWN |
| NONMMUT056814.2 | 6:48436637-48437152 | 516 | 4.2580001 | 0.00429367 | 0.44045271 | UP |
| NONMMUT035743.2 | 2:11493903-11501296 | 594 | Inf | 0.00435879 | 0.44587351 | UP |
| ENSMUST00000130892 | 5:110167508-110176504 | 1250 | #NAME? | 0.004391 | 0.44790668 | DOWN |
| NONMMUT028282.2 | 17:13032591-13040021 | 5465 | #NAME? | 0.00446485 | 0.4541643 | DOWN |
| ENSMUST00000192051 | 9:107564357-107566787 | 682 | 2.37451876 | 0.00451973 | 0.45846194 | UP |
| NONMMUT021642.2 | 14:75229436-75230816 | 1381 | 1.87645144 | 0.00459735 | 0.46106553 | UP |
| NONMMUT025080.2 | 15:101221247-101222155 | 583 | #NAME? | 0.00466587 | 0.46106553 | DOWN |
| NONMMUT031008.2 | 17:86656035-86657918 | 1884 | 3.25739903 | 0.00462644 | 0.46106553 | UP |
| NONMMUT048652.2 | 4:109402280-109406254 | 3383 | -3.343652 | 0.00462966 | 0.46106553 | DOWN |
| NONMMUT070331.2 | 9:88447910-88448943 | 1034 | 3.12451996 | 0.0046507 | 0.46106553 | UP |
| NONMMUT088596.1 | 12:75444133-75445440 | 1308 | -3.9606072 | 0.00467236 | 0.46106553 | DOWN |
| NONMMUT100190.1 | 16:75205655-75206289 | 635 | 2.88024154 | 0.00457916 | 0.46106553 | UP |
| NONMMUT130486.1 | 8:34686737-34687906 | 333 | Inf | 0.00466959 | 0.46106553 | UP |
| NONMMUT140454.1 | 11:60792301-60794837 | 773 | #NAME? | 0.00461991 | 0.46106553 | DOWN |
| NONMMUT140520.1 | 11:76494883-76496102 | 825 | 2.7877415 | 0.00466476 | 0.46106553 | UP |
| NONMMUT100101.1 | 16:74895292-74903594 | 8303 | Inf | 0.00468511 | 0.46107084 | UP |
| NONMMUT017742.2 | 13:58125880-58127070 | 1191 | 2.48718864 | 0.00473031 | 0.4632389 | UP |
| NONMMUT140534.1 | 11:78962981-78984866 | 1433 | 1.93447773 | 0.00473265 | 0.4632389 | UP |
| ENSMUST00000123403 | 2:122708043-122721456 | 1233 | 5.89254896 | 0.00475881 | 0.46374445 | UP |
| NONMMUT053993.2 | 5:114754252-114758282 | 4031 | 1.94393184 | 0.00476336 | 0.46374445 | UP |
| ENSMUST00000186147 | 6:82877864-82881853 | 1833 | -3.0484736 | 0.00482763 | 0.46749443 | DOWN |
| NONMMUT027272.2 | 16:75031907-75034931 | 3025 | Inf | 0.00482709 | 0.46749443 | UP |
| NONMMUT047572.2 | 4:62360701-62363550 | 2850 | 3.32806798 | 0.00487597 | 0.47092018 | UP |
| NONMMUT152519.1 | 8:11508558-11510867 | 1641 | #NAME? | 0.0048992 | 0.471909 | DOWN |
| NONMMUT081509.1 | 10:9497269-9499904 | 2636 | Inf | 0.00493121 | 0.47373546 | UP |
| NONMMUT096084.1 | 15:76558630-76559865 | 1236 | -2.703813 | 0.00495632 | 0.47489123 | DOWN |
| NONMMUT009722.2 | 11:52477312-52479087 | 643 | #NAME? | 0.00504302 | 0.48192676 | DOWN |
| NONMMUT148079.1 | 4:69691954-69692304 | 351 | #NAME? | 0.00508101 | 0.48428276 | DOWN |
| NONMMUT017121.2 | 13:41236092-41243561 | 7286 | -3.1347174 | 0.0051488 | 0.48907012 | DOWN |
| NONMMUT058644.2 | 6:120033522-120036471 | 2233 | Inf | 0.00518431 | 0.48907012 | UP |
| NONMMUT062849.2 | 7:98177194-98184799 | 3157 | Inf | 0.00518511 | 0.48907012 | UP |
| NONMMUT079290.1 | 1:171320542-171326829 | 6288 | 2.97797931 | 0.00517574 | 0.48907012 | UP |
| NONMMUT064397.2 | 8:3455524-3456604 | 1081 | 4.33219779 | 0.00521293 | 0.49042085 | UP |
| NONMMUT011814.2 | 11:97662192-97663122 | 827 | 3.83247325 | 0.00523404 | 0.49113393 | UP |
| NONMMUT071047.2 | 9:109978974-110005780 | 3222 | -4.0384702 | 0.00536209 | 0.4992795 | DOWN |
| NONMMUT100120.1 | 16:74977712-74980174 | 2463 | Inf | 0.00535981 | 0.4992795 | UP |
| NONMMUT100156.1 | 16:75105429-75106960 | 1532 | 3.27659991 | 0.00535304 | 0.4992795 | UP |
| NONMMUT090870.1 | 13:15909980-15911344 | 336 | Inf | 0.00539565 | 0.50111944 | UP |
| NONMMUT025316.2 | 16:5246014-5251977 | 5287 | 3.86557416 | 0.00541214 | 0.50136877 | UP |
| NONMMUT033609.2 | 19:5796056-5802661 | 6142 | Inf | 0.00543809 | 0.50226001 | UP |
| NONMMUT154369.1 | X:145602115-145602322 | 208 | 3.5149625 | 0.00544943 | 0.50226001 | UP |
| NONMMUT091401.1 | 13:51635821-51638546 | 2726 | 1.64819627 | 0.00548722 | 0.50446264 | UP |
| MSTRG.18275.2 | 19:40508404-40508851 | 318 | Inf | 0.00553982 | 0.50457399 | UP |
| NONMMUT009594.2 | 11:50222452-50225236 | 2634 | Inf | 0.00553915 | 0.50457399 | UP |
| NONMMUT034985.2 | 19:47487501-47495921 | 8327 | Inf | 0.00554401 | 0.50457399 | UP |
| NONMMUT143556.1 | 14:100273586-100284964 | 11275 | -3.8675582 | 0.00553335 | 0.50457399 | DOWN |
| NONMMUT050780.2 | 4:152015955-152017677 | 1723 | 3.22944626 | 0.00556022 | 0.50478417 | UP |
| NONMMUT134170.1 | 9:41939568-41966887 | 547 | 3.86231487 | 0.00567927 | 0.51430646 | UP |
| NONMMUT048656.2 | 4:109402313-109406316 | 2557 | 1.7941215 | 0.00574046 | 0.51843349 | UP |
| NONMMUT094571.1 | 14:69582162-69591373 | 2848 | -4.0573578 | 0.00575339 | 0.51843349 | DOWN |
| NONMMUT038951.2 | 2:105153846-105157238 | 3387 | Inf | 0.00577089 | 0.5187231 | UP |
| NONMMUT115002.1 | 4:55534711-55599297 | 1742 | -2.7027017 | 0.00581355 | 0.52126716 | DOWN |
| NONMMUT011749.2 | 11:96807380-96808147 | 768 | 3.93050564 | 0.00585142 | 0.523371 | UP |
| NONMMUT152446.1 | 8:122033538-122034940 | 1403 | -3.8763603 | 0.00592691 | 0.52881997 | DOWN |
| NONMMUT136825.1 | X:23607111-23608105 | 995 | 2.02829177 | 0.00595046 | 0.52962002 | UP |
| ENSMUST00000182756 | 14:61632299-61648622 | 3530 | -1.9595589 | 0.00599192 | 0.53200595 | DOWN |
| NONMMUT113641.1 | 3:94994411-94997351 | 2234 | 2.21554821 | 0.00609339 | 0.53969609 | UP |
| NONMMUT089908.1 | 13:55186886-55188831 | 1946 | 2.39684023 | 0.0061529 | 0.5436413 | UP |
| NONMMUT081536.1 | 10:9567967-9570245 | 2279 | Inf | 0.00617546 | 0.54430962 | UP |
| ENSMUST00000225163 | 14:67169588-67209261 | 1138 | 1.93385696 | 0.00628694 | 0.55145844 | UP |
| NONMMUT018160.2 | 13:69633903-69634334 | 432 | 3.58303992 | 0.00627728 | 0.55145844 | UP |
| MSTRG.33001.1 | 7:104348219-104351647 | 1210 | 1.77704978 | 0.00638479 | 0.55601251 | UP |
| NONMMUT000544.2 | 1:36142212-36143655 | 1444 | 2.86731996 | 0.00637718 | 0.55601251 | UP |
| NONMMUT054315.2 | 5:121806139-121809056 | 2918 | -2.5367463 | 0.00637958 | 0.55601251 | DOWN |
| NONMMUT008438.2 | 11:5755007-5757381 | 2375 | -3.0757051 | 0.0064264 | 0.5576523 | DOWN |
| NONMMUT110167.1 | 2:168060871-168064049 | 1975 | 2.25121651 | 0.00645711 | 0.5576523 | UP |
| NONMMUT138593.1 | 1:55027423-55028340 | 918 | Inf | 0.00646504 | 0.5576523 | UP |
| NONMMUT152692.1 | 8:46725827-46739562 | 6424 | Inf | 0.00646448 | 0.5576523 | UP |
| NONMMUT095724.1 | 15:39178123-39178746 | 624 | -3.1702778 | 0.00657766 | 0.56602175 | DOWN |
| NONMMUT046091.2 | 4:8393516-8396644 | 2906 | 2.18112538 | 0.00662863 | 0.56678667 | UP |
| NONMMUT081592.1 | 10:17190024-17202188 | 1156 | #NAME? | 0.00663337 | 0.56678667 | DOWN |
| NONMMUT117392.1 | 4:132614614-132618142 | 3529 | 3.30509438 | 0.00662742 | 0.56678667 | UP |
| NONMMUT035137.2 | 19:55519042-55521237 | 2196 | -1.3943119 | 0.00670268 | 0.57032439 | DOWN |
| NONMMUT068127.2 | 9:14331441-14333101 | 1661 | -2.4801016 | 0.00672189 | 0.57032439 | DOWN |
| NONMMUT146749.1 | 2:29188815-29194202 | 2744 | 4.45852642 | 0.00671609 | 0.57032439 | UP |
| NONMMUT035224.2 | 19:57511350-57512668 | 955 | Inf | 0.00678522 | 0.57246123 | UP |
| NONMMUT043008.2 | 3:51685913-51687789 | 1877 | 2.40347057 | 0.00679437 | 0.57246123 | UP |
| NONMMUT073221.2 | X:94977217-94986923 | 4367 | #NAME? | 0.0067775 | 0.57246123 | DOWN |
| ENSMUST00000138764 | 2:118069847-118111091 | 2562 | Inf | 0.00681077 | 0.57251484 | UP |
| NONMMUT119574.1 | 5:136915929-136937113 | 5389 | Inf | 0.0068592 | 0.57525365 | UP |
| ENSMUST00000180537 | 10:22402883-22407470 | 4478 | Inf | 0.00705241 | 0.57723443 | UP |
| ENSMUST00000223906 | 13:41476526-41477900 | 1375 | Inf | 0.0070677 | 0.57723443 | UP |
| NONMMUT008778.2 | 11:20453070-20456035 | 2804 | #NAME? | 0.00690143 | 0.57723443 | DOWN |
| NONMMUT020270.2 | 14:31943090-31946345 | 2639 | Inf | 0.00694951 | 0.57723443 | UP |
| NONMMUT027572.2 | 16:87731517-87733340 | 1824 | 2.38611543 | 0.00697558 | 0.57723443 | UP |
| NONMMUT032401.2 | 18:54981435-54987689 | 3340 | -1.5125534 | 0.00699693 | 0.57723443 | DOWN |
| NONMMUT063981.2 | 7:133116534-133119545 | 3012 | -2.6353246 | 0.00707356 | 0.57723443 | DOWN |
| NONMMUT081507.1 | 10:9491709-9492582 | 874 | Inf | 0.00703135 | 0.57723443 | UP |
| NONMMUT089310.1 | 13:14667709-14671442 | 3734 | Inf | 0.00703245 | 0.57723443 | UP |
| NONMMUT107753.1 | 2:117192415-117193366 | 952 | Inf | 0.0070521 | 0.57723443 | UP |
| NONMMUT119683.1 | 5:142832167-142833016 | 850 | Inf | 0.00704486 | 0.57723443 | UP |
| NONMMUT145212.1 | 17:69380729-69382760 | 2032 | Inf | 0.00704431 | 0.57723443 | UP |
| NONMMUT094975.1 | 14:105232049-105259272 | 1305 | #NAME? | 0.00709703 | 0.57785063 | DOWN |
| NONMMUT147378.1 | 3:62159004-62159374 | 371 | -2.1922913 | 0.00718079 | 0.58336264 | DOWN |
| NONMMUT033507.2 | 19:4760112-4761342 | 1231 | 2.43145972 | 0.00720568 | 0.58407815 | UP |
| NONMMUT028444.2 | 17:17887851-17890807 | 2957 | 3.0065666 | 0.0072538 | 0.58645226 | UP |
| NONMMUT060510.2 | 7:29761755-29767612 | 5205 | 3.68374106 | 0.00726727 | 0.58645226 | UP |
| NONMMUT041303.2 | 2:167664574-167668408 | 1498 | Inf | 0.00736534 | 0.59253061 | UP |
| NONMMUT057055.2 | 6:54501080-54503762 | 2683 | 2.628287 | 0.00737522 | 0.59253061 | UP |
| ENSMUST00000183083 | 18:61639542-61647292 | 483 | 3.95786268 | 0.007498 | 0.59989184 | UP |
| NONMMUT039975.2 | 2:131733454-131749179 | 1961 | 3.95786268 | 0.00749989 | 0.59989184 | UP |
| NONMMUT100129.1 | 16:75012899-75017878 | 4980 | 2.78348031 | 0.0075223 | 0.60036196 | UP |
| NONMMUT024873.2 | 15:97790924-97792688 | 1765 | 2.91148965 | 0.0075661 | 0.60177384 | UP |
| NONMMUT057781.2 | 6:86527336-86551096 | 934 | Inf | 0.00757313 | 0.60177384 | UP |
| NONMMUT039378.2 | 2:119733977-119735402 | 1426 | -2.8807342 | 0.00767817 | 0.60426871 | DOWN |
| NONMMUT068395.2 | 9:25521491-25524561 | 3071 | 2.51533115 | 0.00769317 | 0.60426871 | UP |
| NONMMUT071231.2 | 9:115281092-115282070 | 979 | -2.2543138 | 0.007638 | 0.60426871 | DOWN |
| NONMMUT071995.2 | X:20961676-20987349 | 1778 | #NAME? | 0.00770437 | 0.60426871 | DOWN |
| NONMMUT118336.1 | 5:43861755-43863170 | 1416 | 1.75278733 | 0.00768383 | 0.60426871 | UP |
| NONMMUT140524.1 | 11:77884698-77886792 | 782 | #NAME? | 0.00769183 | 0.60426871 | DOWN |
| NONMMUT053709.2 | 5:109866744-109885858 | 4292 | -4.3900134 | 0.00776795 | 0.60794218 | DOWN |
| NONMMUT097788.1 | 16:3777011-3785739 | 8384 | Inf | 0.00779883 | 0.60801218 | UP |
| NONMMUT147218.1 | 2:167827153-167834876 | 7724 | Inf | 0.00780233 | 0.60801218 | UP |
| NONMMUT002589.2 | 1:132064851-132067809 | 2959 | -3.4421265 | 0.00785522 | 0.60934736 | DOWN |
| NONMMUT081525.1 | 10:9543849-9545726 | 1878 | Inf | 0.00786964 | 0.60934736 | UP |
| NONMMUT100166.1 | 16:75127907-75130819 | 2913 | 2.11176461 | 0.0078698 | 0.60934736 | UP |
| NONMMUT029704.2 | 17:43145000-43157258 | 10241 | -1.6309703 | 0.00794137 | 0.61358079 | DOWN |
| NONMMUT010582.2 | 11:72047530-72048427 | 898 | -2.0253002 | 0.00803596 | 0.61957095 | DOWN |
| NONMMUT095726.1 | 15:39179435-39181616 | 2182 | -2.4969479 | 0.00806019 | 0.6201222 | DOWN |
| NONMMUT007729.2 | 10:121510337-121512079 | 1743 | -2.4194984 | 0.00813571 | 0.62219285 | DOWN |
| NONMMUT050476.2 | 4:144963270-144970742 | 2014 | #NAME? | 0.0081385 | 0.62219285 | DOWN |
| NONMMUT145710.1 | 18:75358508-75366137 | 3860 | #NAME? | 0.00812557 | 0.62219285 | DOWN |
| NONMMUT026369.2 | 16:33767090-33767821 | 732 | 3.38564501 | 0.00829441 | 0.63277991 | UP |
| NONMMUT021993.2 | 14:99870650-99873629 | 2980 | -1.4323612 | 0.00837372 | 0.63571654 | DOWN |
| NONMMUT027930.2 | 16:98063877-98082001 | 3150 | Inf | 0.00835521 | 0.63571654 | UP |
| NONMMUT081521.1 | 10:9521482-9523841 | 2360 | 3.9310996 | 0.00838542 | 0.63571654 | UP |
| NONMMUT112840.1 | 3:23938905-23940211 | 1307 | Inf | 0.00848538 | 0.63963031 | UP |
| NONMMUT146328.1 | 2:68796335-68803050 | 6716 | Inf | 0.00848845 | 0.63963031 | UP |
| NONMMUT147949.1 | 4:11123553-11127044 | 3410 | Inf | 0.00848989 | 0.63963031 | UP |
| NONMMUT031138.2 | 17:91088893-91092736 | 851 | Inf | 0.0085087 | 0.63972013 | UP |
| ENSMUST00000180598 | 1:192136896-192151026 | 2615 | Inf | 0.00856282 | 0.64134539 | UP |
| NONMMUT006922.2 | 10:94673493-94688613 | 4135 | 2.74461965 | 0.00856564 | 0.64134539 | UP |
| NONMMUT092045.1 | 13:93420319-93422386 | 2068 | #NAME? | 0.00868384 | 0.6467796 | DOWN |
| NONMMUT141486.1 | 12:73868274-73873428 | 5155 | -1.9138718 | 0.00869165 | 0.6467796 | DOWN |
| NONMMUT145462.1 | 18:68691712-68692315 | 561 | -2.0827474 | 0.00866598 | 0.6467796 | DOWN |
| NONMMUT088276.1 | 12:34817756-34819606 | 1851 | 2.01832482 | 0.00872889 | 0.64689954 | UP |
| NONMMUT145134.1 | 17:43263609-43267933 | 4325 | 2.07178569 | 0.00872108 | 0.64689954 | UP |
| NONMMUT005529.2 | 10:47452985-47455800 | 2778 | 4.23759448 | 0.00879076 | 0.64752042 | UP |
| NONMMUT018802.2 | 13:97898595-97899591 | 997 | -2.4598082 | 0.00878229 | 0.64752042 | DOWN |
| NONMMUT143162.1 | 14:60176841-60181537 | 3032 | Inf | 0.00877309 | 0.64752042 | UP |
| NONMMUT072388.2 | X:50564794-50595616 | 2804 | Inf | 0.0088279 | 0.6488722 | UP |
| NONMMUT140866.1 | 11:43378806-43380061 | 1256 | 2.39490247 | 0.00884485 | 0.6488722 | UP |
| NONMMUT088242.1 | 12:34708278-34708928 | 651 | 2.32137746 | 0.00894178 | 0.65466079 | UP |
| NONMMUT056918.2 | 6:51571430-51575709 | 4280 | 1.79824732 | 0.00899577 | 0.6572885 | UP |
| NONMMUT111509.1 | 3:86148937-86150174 | 1238 | #NAME? | 0.00904786 | 0.6597673 | DOWN |
| NONMMUT037124.2 | 2:45075438-45077893 | 2456 | 2.06835162 | 0.00916578 | 0.66702628 | UP |
| NONMMUT104872.1 | 18:74972779-74974555 | 1550 | #NAME? | 0.00920749 | 0.66872138 | DOWN |
| NONMMUT083940.1 | 11:75172423-75177634 | 947 | Inf | 0.00922752 | 0.66883836 | UP |
| NONMMUT025897.2 | 16:21794341-21809047 | 602 | 3.15084217 | 0.00932503 | 0.67455978 | UP |
| NONMMUT035729.2 | 2:11312557-11320612 | 4738 | 2.4450188 | 0.00939081 | 0.67712903 | UP |
| NONMMUT140922.1 | 11:59196880-59202431 | 3781 | #NAME? | 0.00939784 | 0.67712903 | DOWN |
| ENSMUST00000132973 | 11:100278706-100291286 | 747 | -2.5721808 | 0.00943085 | 0.67795503 | DOWN |
| NONMMUT059632.2 | 6:149172862-149178700 | 5839 | 1.65308032 | 0.0094468 | 0.67795503 | UP |
| NONMMUT080008.1 | 10:37337487-37398587 | 3428 | Inf | 0.00946531 | 0.67795503 | UP |
| NONMMUT100174.1 | 16:75153623-75154744 | 1122 | 2.29530177 | 0.00949449 | 0.67870647 | UP |
| NONMMUT092818.1 | 14:47745679-47746831 | 1153 | #NAME? | 0.0095624 | 0.68221795 | DOWN |
| NONMMUT035384.2 | 2:3460667-3464044 | 3378 | -2.0931274 | 0.00971068 | 0.68652555 | DOWN |
| NONMMUT043346.2 | 3:65379664-65381765 | 2102 | -2.3411581 | 0.00973148 | 0.68652555 | DOWN |
| NONMMUT044728.2 | 3:107888605-107896477 | 5916 | -3.2272255 | 0.00965631 | 0.68652555 | DOWN |
| NONMMUT048718.2 | 4:111753790-111755595 | 1806 | 2.67172076 | 0.00971923 | 0.68652555 | UP |
| NONMMUT100196.1 | 16:75212817-75213534 | 718 | 1.70758737 | 0.00971069 | 0.68652555 | UP |
| NONMMUT122872.1 | 6:115336644-115361188 | 2266 | -3.5627056 | 0.00973621 | 0.68652555 | DOWN |
| NONMMUT084910.1 | 11:20474597-20477602 | 955 | Inf | 0.00975647 | 0.68662097 | UP |
| NONMMUT003224.2 | 1:155098844-155104285 | 1202 | Inf | 0.00979695 | 0.68680753 | UP |
| NONMMUT018664.2 | 13:93433131-93435062 | 1932 | 2.87358994 | 0.00979522 | 0.68680753 | UP |
| NONMMUT046183.2 | 4:11182125-11191274 | 1248 | Inf | 0.00982589 | 0.68750938 | UP |
| NONMMUT100107.1 | 16:74934712-74940102 | 5391 | 3.81506844 | 0.00991236 | 0.69222553 | UP |
| NONMMUT150895.1 | 7:34390532-34394337 | 3806 | 2.59823154 | 0.01009397 | 0.70355583 | UP |
| NONMMUT072876.2 | X:74451096-74453778 | 2683 | -2.0430932 | 0.01011449 | 0.70363495 | DOWN |
| NONMMUT100203.1 | 16:75221316-75223400 | 2085 | 1.82982517 | 0.01018798 | 0.70703249 | UP |
| NONMMUT143362.1 | 14:24438518-24448154 | 9267 | #NAME? | 0.01022173 | 0.70703249 | DOWN |
| NONMMUT145261.1 | 17:84133783-84137364 | 1732 | #NAME? | 0.01022154 | 0.70703249 | DOWN |
| ENSMUST00000204539 | 6:54902422-54905728 | 3307 | #NAME? | 0.01041173 | 0.70827047 | DOWN |
| ENSMUST00000214276 | 9:85332352-85348065 | 3309 | #NAME? | 0.0104141 | 0.70827047 | DOWN |
| NONMMUT004555.2 | 10:5003620-5005615 | 1996 | 2.04729826 | 0.01035597 | 0.70827047 | UP |
| NONMMUT027615.2 | 16:90007932-90011112 | 3181 | -2.7958879 | 0.01042744 | 0.70827047 | DOWN |
| NONMMUT028125.2 | 17:8284460-8288738 | 364 | #NAME? | 0.01045266 | 0.70827047 | DOWN |
| NONMMUT032025.2 | 18:36801763-36802107 | 345 | -5.4507613 | 0.01035895 | 0.70827047 | DOWN |
| NONMMUT040857.2 | 2:158353700-158361509 | 3159 | #NAME? | 0.01030504 | 0.70827047 | DOWN |
| NONMMUT073404.2 | X:102315277-102317458 | 2182 | Inf | 0.01045231 | 0.70827047 | UP |
| NONMMUT103964.1 | 18:3248370-3249480 | 1111 | -1.6044994 | 0.01039014 | 0.70827047 | DOWN |
| NONMMUT145424.1 | 18:46629578-46642939 | 3442 | #NAME? | 0.01045326 | 0.70827047 | DOWN |
| NONMMUT149212.1 | 5:135622606-135628962 | 6357 | 1.38536429 | 0.01045418 | 0.70827047 | UP |
| ENSMUST00000218957 | 10:9482929-9487202 | 4274 | 2.54091375 | 0.01051716 | 0.7098887 | UP |
| NONMMUT028187.2 | 17:10276220-10278894 | 2675 | 2.52506498 | 0.01050798 | 0.7098887 | UP |
| NONMMUT021320.2 | 14:65196653-65200026 | 3374 | 2.10475179 | 0.01057482 | 0.71032089 | UP |
| NONMMUT037122.2 | 2:45053788-45057542 | 3755 | 2.3904634 | 0.01058224 | 0.71032089 | UP |
| NONMMUT044520.2 | 3:102769441-102772208 | 2768 | 3.4810575 | 0.01055139 | 0.71032089 | UP |
| ENSMUST00000101007 | 11:69057718-69060483 | 2766 | 1.79712615 | 0.01062589 | 0.71193467 | UP |
| NONMMUT148528.1 | 4:86836560-86838936 | 1559 | #NAME? | 0.01065074 | 0.71228526 | DOWN |
| NONMMUT151159.1 | 7:90029159-90049072 | 11004 | 3.24638091 | 0.01075323 | 0.71781749 | UP |
| ENSMUST00000141797 | 17:29114145-29133693 | 475 | Inf | 0.01104635 | 0.72291723 | UP |
| ENSMUST00000190911 | 4:141875707-141877033 | 1327 | 1.45835413 | 0.01098537 | 0.72291723 | UP |
| ENSMUST00000200437 | 3:90292301-90293983 | 1683 | Inf | 0.01104861 | 0.72291723 | UP |
| ENSMUST00000209416 | 7:45568101-45575147 | 856 | Inf | 0.0110441 | 0.72291723 | UP |
| ENSMUST00000227651 | 14:105590057-105593904 | 3388 | -2.516946 | 0.01101824 | 0.72291723 | DOWN |
| NONMMUT003017.2 | 1:143598965-143601039 | 2075 | 2.43527298 | 0.01097898 | 0.72291723 | UP |
| NONMMUT012434.2 | 11:107208534-107211796 | 3263 | 2.10723059 | 0.01103224 | 0.72291723 | UP |
| NONMMUT060991.2 | 7:43511120-43518551 | 1109 | Inf | 0.01104217 | 0.72291723 | UP |
| NONMMUT072383.2 | X:50563069-50595617 | 3837 | Inf | 0.01100239 | 0.72291723 | UP |
| NONMMUT100227.1 | 16:75277510-75278565 | 1056 | 1.62972114 | 0.01096715 | 0.72291723 | UP |
| NONMMUT136920.1 | X:53050550-53057191 | 3958 | Inf | 0.01102989 | 0.72291723 | UP |
| NONMMUT081391.1 | 10:9108994-9110924 | 1931 | Inf | 0.01107032 | 0.72303546 | UP |
| NONMMUT045865.2 | 3:154576526-154579157 | 2632 | -2.3619249 | 0.01123294 | 0.72753 | DOWN |
| NONMMUT056926.2 | 6:51909747-51911201 | 1455 | 1.7200273 | 0.01133357 | 0.72753 | UP |
| NONMMUT057566.2 | 6:82879311-82883827 | 257 | 3.91534888 | 0.01123997 | 0.72753 | UP |
| NONMMUT062847.2 | 7:98177194-98184799 | 3154 | #NAME? | 0.01133165 | 0.72753 | DOWN |
| NONMMUT071996.2 | X:20962114-20987349 | 2024 | 2.58123535 | 0.01126177 | 0.72753 | UP |
| NONMMUT077977.1 | 1:69448480-69449215 | 736 | -2.42026 | 0.01125948 | 0.72753 | DOWN |
| NONMMUT094819.1 | 14:100191989-100193960 | 1972 | -3.9071107 | 0.01122193 | 0.72753 | DOWN |
| NONMMUT098315.1 | 16:36099021-36100357 | 1337 | -2.7521922 | 0.01116782 | 0.72753 | DOWN |
| NONMMUT107953.1 | 2:131408677-131409743 | 1067 | -2.0554725 | 0.01130092 | 0.72753 | DOWN |
| NONMMUT145279.1 | 17:84877538-84879345 | 1808 | -1.90903 | 0.01133948 | 0.72753 | DOWN |
| NONMMUT025725.2 | 16:17937399-17941230 | 3355 | Inf | 0.01142077 | 0.72893164 | UP |
| NONMMUT039730.2 | 2:128311684-128315999 | 4296 | Inf | 0.01141928 | 0.72893164 | UP |
| NONMMUT059702.2 | 7:3640438-3644486 | 2686 | Inf | 0.01142155 | 0.72893164 | UP |
| NONMMUT069643.2 | 9:65904679-65908381 | 3703 | -3.0131845 | 0.0114494 | 0.7294274 | DOWN |
| NONMMUT043457.2 | 3:69558433-69560799 | 2367 | 2.60688086 | 0.01150784 | 0.73186633 | UP |
| NONMMUT025212.2 | 16:3861734-3864712 | 2979 | 2.61334993 | 0.01154381 | 0.73287038 | UP |
| NONMMUT100149.1 | 16:75085701-75087010 | 1310 | 2.61056024 | 0.01177747 | 0.74551621 | UP |
| NONMMUT104322.1 | 18:34555810-34556524 | 715 | 2.9249596 | 0.01180503 | 0.74551621 | UP |
| NONMMUT134571.1 | 9:65537795-65538896 | 1102 | 4.00698575 | 0.01182512 | 0.74551621 | UP |
| NONMMUT146236.1 | 2:26905640-26910640 | 1683 | 3.11091527 | 0.01179362 | 0.74551621 | UP |
| ENSMUST00000101522 | 5:23431810-23433919 | 2110 | 1.31856933 | 0.01219372 | 0.75621289 | UP |
| ENSMUST00000144002 | 4:114909257-114921967 | 1015 | 1.91916687 | 0.01222094 | 0.75621289 | UP |
| NONMMUT003664.2 | 1:166395164-166397963 | 2800 | 1.86462142 | 0.01211586 | 0.75621289 | UP |
| NONMMUT005859.2 | 10:62505861-62506910 | 1050 | 1.98070354 | 0.01220312 | 0.75621289 | UP |
| NONMMUT033779.2 | 19:8019559-8020611 | 1053 | 2.08268537 | 0.01217052 | 0.75621289 | UP |
| NONMMUT037121.2 | 2:45029872-45031401 | 1530 | 4.10773555 | 0.01230715 | 0.75621289 | UP |
| NONMMUT055370.2 | 5:148953740-148967306 | 13274 | #NAME? | 0.0120872 | 0.75621289 | DOWN |
| NONMMUT056952.2 | 6:52208062-52214575 | 1871 | Inf | 0.01227754 | 0.75621289 | UP |
| NONMMUT075374.1 | 1:43580891-43581832 | 942 | -2.2509539 | 0.01224785 | 0.75621289 | DOWN |
| NONMMUT077171.1 | 1:193281256-193283722 | 2467 | Inf | 0.01216645 | 0.75621289 | UP |
| NONMMUT089909.1 | 13:55190441-55191906 | 1466 | Inf | 0.01207736 | 0.75621289 | UP |
| NONMMUT100170.1 | 16:75142309-75143233 | 925 | 2.25008834 | 0.01230404 | 0.75621289 | UP |
| NONMMUT100229.1 | 16:75281647-75282819 | 1173 | 1.87483669 | 0.01223928 | 0.75621289 | UP |
| NONMMUT132267.1 | 9:32086128-32087419 | 1292 | 1.68619554 | 0.01203 | 0.75621289 | UP |
| NONMMUT138555.1 | 1:31015278-31024280 | 7086 | Inf | 0.01227764 | 0.75621289 | UP |
| NONMMUT026103.2 | 16:28612409-28615094 | 2091 | 1.66590399 | 0.01250925 | 0.76346388 | UP |
| NONMMUT057779.2 | 6:86527154-86577995 | 1230 | -4.5516398 | 0.01245358 | 0.76346388 | DOWN |
| NONMMUT071558.2 | 9:124423251-124424856 | 1210 | #NAME? | 0.0124946 | 0.76346388 | DOWN |
| NONMMUT109199.1 | 2:72605937-72620311 | 1052 | #NAME? | 0.01247248 | 0.76346388 | DOWN |
| NONMMUT101999.1 | 17:31360492-31362089 | 1598 | -3.5264201 | 0.0127604 | 0.77618306 | DOWN |
| NONMMUT145007.1 | 17:3062029-3075869 | 1883 | #NAME? | 0.01275429 | 0.77618306 | DOWN |
| NONMMUT040436.2 | 2:151039517-151040970 | 1454 | #NAME? | 0.01293512 | 0.78549493 | DOWN |
| NONMMUT100118.1 | 16:74965838-74967979 | 1604 | 3.70000442 | 0.0129929 | 0.78768624 | UP |
| NONMMUT000986.2 | 1:56971469-56975196 | 3728 | 2.57821871 | 0.01307002 | 0.79104107 | UP |
| NONMMUT039830.2 | 2:129596327-129597219 | 893 | Inf | 0.01312464 | 0.79302528 | UP |
| NONMMUT140604.1 | 11:88718442-88728572 | 2645 | #NAME? | 0.01324545 | 0.79899542 | DOWN |
| ENSMUST00000130240 | 7:124625670-124708935 | 664 | -1.5350184 | 0.01331091 | 0.80161244 | DOWN |
| NONMMUT103951.1 | 18:90552022-90572167 | 773 | #NAME? | 0.01334301 | 0.80221539 | DOWN |
| NONMMUT029746.2 | 17:44106019-44121620 | 2354 | 2.48378458 | 0.01336698 | 0.80232784 | UP |
| NONMMUT001262.2 | 1:64032868-64035586 | 2719 | -1.8695331 | 0.01345252 | 0.8025283 | DOWN |
| NONMMUT003984.2 | 1:178963898-178965282 | 1385 | -3.4500508 | 0.01350291 | 0.8025283 | DOWN |
| NONMMUT018229.2 | 13:73515748-73516412 | 665 | 2.24111612 | 0.01345654 | 0.8025283 | UP |
| NONMMUT088271.1 | 12:34805175-34806240 | 1066 | 2.06855834 | 0.01349314 | 0.8025283 | UP |
| NONMMUT096548.1 | 15:102004197-102012608 | 1376 | 1.83667641 | 0.01346834 | 0.8025283 | UP |
| NONMMUT119633.1 | 5:138687761-138688802 | 1042 | -2.4945777 | 0.0134585 | 0.8025283 | DOWN |
| NONMMUT030533.2 | 17:74055420-74080280 | 1723 | Inf | 0.01362376 | 0.80838771 | UP |
| NONMMUT099601.1 | 16:30946183-30947790 | 1608 | 3.65657245 | 0.01368587 | 0.80856134 | UP |
| NONMMUT104525.1 | 18:46422703-46425664 | 2962 | 3.65657245 | 0.01369349 | 0.80856134 | UP |
| NONMMUT119779.1 | 5:147901787-147903674 | 1888 | 3.65657245 | 0.01367813 | 0.80856134 | UP |
| NONMMUT000922.2 | 1:53698045-53706811 | 847 | Inf | 0.01374546 | 0.80895896 | UP |
| NONMMUT035073.2 | 19:53450464-53451507 | 824 | Inf | 0.01377518 | 0.80895896 | UP |
| NONMMUT139779.1 | 10:118254009-118259758 | 321 | -3.2512973 | 0.01378933 | 0.80895896 | DOWN |
| NONMMUT144976.1 | 17:79609874-79614762 | 4716 | Inf | 0.01378767 | 0.80895896 | UP |
| NONMMUT030151.2 | 17:57078590-57079546 | 957 | -2.2816204 | 0.01396136 | 0.81159869 | DOWN |
| NONMMUT039393.2 | 2:119965003-119966807 | 1805 | 2.42009185 | 0.0139424 | 0.81159869 | UP |
| NONMMUT081533.1 | 10:9564505-9565387 | 883 | Inf | 0.01396467 | 0.81159869 | UP |
| NONMMUT101896.1 | 17:22768027-22772249 | 4223 | Inf | 0.01396842 | 0.81159869 | UP |
| NONMMUT117071.1 | 4:103168815-103172565 | 476 | Inf | 0.01396534 | 0.81159869 | UP |
| NONMMUT153410.1 | 9:90349010-90354120 | 5111 | Inf | 0.01396234 | 0.81159869 | UP |
| NONMMUT008595.2 | 11:11770712-11772103 | 1392 | 2.93281528 | 0.01402459 | 0.81356039 | UP |
| NONMMUT117292.1 | 4:126015718-126016711 | 994 | Inf | 0.01406081 | 0.81436101 | UP |
| NONMMUT028268.2 | 17:12960512-12961559 | 1048 | #NAME? | 0.01430063 | 0.82507583 | DOWN |
| NONMMUT049421.2 | 4:125110281-125111512 | 1232 | -2.3568676 | 0.01433899 | 0.82507583 | DOWN |
| NONMMUT060450.2 | 7:28378641-28392363 | 234 | Inf | 0.01434858 | 0.82507583 | UP |
| NONMMUT064943.2 | 8:24412360-24416891 | 1061 | #NAME? | 0.01435942 | 0.82507583 | DOWN |
| NONMMUT065148.2 | 8:33428796-33432014 | 3219 | #NAME? | 0.01430385 | 0.82507583 | DOWN |
| ENSMUST00000186819 | 13:5855508-5857712 | 544 | #NAME? | 0.01438764 | 0.82539162 | DOWN |
| NONMMUT052319.2 | 5:53268392-53269265 | 874 | 2.47401011 | 0.01446252 | 0.82837823 | UP |
| NONMMUT100168.1 | 16:75140479-75141495 | 1017 | 2.12148312 | 0.01454745 | 0.83193073 | UP |
| NONMMUT076431.1 | 1:136642336-136642978 | 643 | Inf | 0.01469917 | 0.83928565 | UP |
| NONMMUT015954.2 | 12:111265706-111267155 | 1450 | 3.61676472 | 0.01476899 | 0.84194828 | UP |
| NONMMUT008659.2 | 11:16915768-16918152 | 2385 | 2.88516928 | 0.0148417 | 0.84476729 | UP |
| NONMMUT040379.2 | 2:148437549-148440679 | 3131 | 2.14620215 | 0.01486809 | 0.84494506 | UP |
| NONMMUT056347.2 | 6:35121522-35122462 | 941 | 3.07474492 | 0.01489149 | 0.84495219 | UP |
| MSTRG.24484.1 | 4:55711692-55729494 | 670 | -1.3675546 | 0.01499744 | 0.8496364 | DOWN |
| ENSMUST00000181020 | X:103493558-103506425 | 3802 | -2.6303532 | 0.01504011 | 0.85043295 | DOWN |
| NONMMUT105384.1 | 19:29067327-29069474 | 812 | 2.71112746 | 0.01505834 | 0.85043295 | UP |
| NONMMUT049753.2 | 4:130823253-130826309 | 3057 | 2.56355301 | 0.01517436 | 0.85565469 | UP |
| NONMMUT041801.2 | 2:180034821-180035459 | 639 | -1.8171595 | 0.01531769 | 0.86239762 | DOWN |
| NONMMUT003019.2 | 1:143604101-143606608 | 2508 | -2.3816241 | 0.0154735 | 0.86446858 | DOWN |
| NONMMUT012087.2 | 11:101641602-101641976 | 375 | Inf | 0.01544036 | 0.86446858 | UP |
| NONMMUT029447.2 | 17:35480139-35480728 | 543 | 2.36083803 | 0.01544403 | 0.86446858 | UP |
| NONMMUT094524.1 | 14:66949354-66951251 | 1898 | -2.1650021 | 0.01545802 | 0.86446858 | DOWN |
| NONMMUT153943.1 | 9:96328370-96329777 | 1408 | -2.3612304 | 0.01545946 | 0.86446858 | DOWN |
| NONMMUT065118.2 | 8:31845100-31849034 | 3935 | Inf | 0.01568047 | 0.87334443 | UP |
| NONMMUT070131.2 | 9:78175914-78178882 | 1982 | Inf | 0.01566182 | 0.87334443 | UP |
| NONMMUT040036.2 | 2:132866211-132868583 | 2373 | 2.0173157 | 0.01586515 | 0.87504711 | UP |
| NONMMUT056880.2 | 6:50293327-50295371 | 2045 | 3.25275525 | 0.01587641 | 0.87504711 | UP |
| NONMMUT067813.2 | 8:128367916-128369989 | 2074 | Inf | 0.01581736 | 0.87504711 | UP |
| NONMMUT072805.2 | X:73670402-73672599 | 1608 | Inf | 0.01586121 | 0.87504711 | UP |
| NONMMUT087168.1 | 12:69197212-69204418 | 3067 | -1.8816163 | 0.01584089 | 0.87504711 | DOWN |
| NONMMUT108511.1 | 2:173707718-173709697 | 1980 | Inf | 0.01587972 | 0.87504711 | UP |
| NONMMUT144330.1 | 16:31459924-31463956 | 2871 | -2.9661629 | 0.01586715 | 0.87504711 | DOWN |
| NONMMUT028383.2 | 17:15771779-15772606 | 828 | -1.5161712 | 0.01593561 | 0.87578224 | DOWN |
| NONMMUT096487.1 | 15:98917717-98940527 | 2586 | 3.52291631 | 0.01594129 | 0.87578224 | UP |
| NONMMUT060994.2 | 7:43524216-43526379 | 2164 | 3.50099619 | 0.01597721 | 0.87642973 | UP |
| NONMMUT100175.1 | 16:75160620-75161845 | 1226 | 2.81036291 | 0.01611402 | 0.88260106 | UP |
| NONMMUT149386.1 | 5:25526619-25528373 | 1755 | 1.87628274 | 0.01615357 | 0.88343468 | UP |
| NONMMUT001399.2 | 1:69531214-69533830 | 2617 | 1.49755396 | 0.01634784 | 0.89151597 | UP |
| NONMMUT060278.2 | 7:25378051-25379277 | 947 | 2.81557132 | 0.01636771 | 0.89151597 | UP |
| NONMMUT117919.1 | 5:9173250-9174335 | 1086 | #NAME? | 0.01637498 | 0.89151597 | DOWN |
| ENSMUST00000218674 | 10:115394750-115397884 | 3135 | 1.73561183 | 0.01644086 | 0.89254818 | UP |
| NONMMUT119341.1 | 5:121295398-121297229 | 229 | -2.1693594 | 0.0164431 | 0.89254818 | DOWN |
| ENSMUST00000175179 | 15:76267135-76267419 | 285 | 3.70848141 | 0.01653462 | 0.89484109 | UP |
| NONMMUT074146.2 | X:142853465-142854906 | 1442 | 1.93470474 | 0.01653406 | 0.89484109 | UP |
| NONMMUT088678.1 | 12:80321924-80324214 | 2291 | -2.0507099 | 0.01656741 | 0.89528095 | DOWN |
| ENSMUST00000173993 | 8:121806415-121807369 | 423 | 3.29112027 | 0.01660224 | 0.89582999 | UP |
| ENSMUST00000128111 | 11:97020508-97026248 | 5272 | #NAME? | 0.01669205 | 0.89933986 | DOWN |
| ENSMUST00000193572 | 9:86684050-86685208 | 1159 | -1.9863689 | 0.01680656 | 0.9006688 | DOWN |
| NONMMUT006580.2 | 10:83722898-83762761 | 1033 | Inf | 0.01681516 | 0.9006688 | UP |
| NONMMUT098692.1 | 16:45945607-45971371 | 4906 | Inf | 0.01679137 | 0.9006688 | UP |
| NONMMUT100765.1 | 17:21859440-21866216 | 2299 | Inf | 0.01681592 | 0.9006688 | UP |
| ENSMUST00000193540 | 1:172388234-172390989 | 2756 | -1.8130302 | 0.01694302 | 0.90613974 | DOWN |
| ENSMUST00000192052 | 1:193259616-193261837 | 2222 | Inf | 0.01700679 | 0.90655105 | UP |
| NONMMUT007081.2 | 10:99220427-99223892 | 1773 | Inf | 0.01705057 | 0.90655105 | UP |
| NONMMUT088439.1 | 12:56091750-56098055 | 1079 | Inf | 0.01704956 | 0.90655105 | UP |
| NONMMUT149236.1 | 5:137890741-137894949 | 1148 | Inf | 0.01705028 | 0.90655105 | UP |
| NONMMUT047234.2 | 4:49510415-49511913 | 1409 | -1.4885634 | 0.01710476 | 0.90677688 | DOWN |
| NONMMUT147725.1 | 3:89289716-89292731 | 2043 | #NAME? | 0.01708756 | 0.90677688 | DOWN |
| NONMMUT133741.1 | 9:121877995-121880075 | 2081 | -1.9332985 | 0.01716029 | 0.90839451 | DOWN |
| NONMMUT044607.2 | 3:104667422-104669627 | 2023 | -2.1912666 | 0.01754335 | 0.92732036 | DOWN |
| ENSMUST00000226101 | 13:35395383-35455832 | 1304 | 2.24946999 | 0.01765186 | 0.92845739 | UP |
| NONMMUT011311.2 | 11:86812765-86816129 | 3365 | 1.61999404 | 0.01769927 | 0.92845739 | UP |
| NONMMUT021992.2 | 14:99866183-99866860 | 678 | -3.1500658 | 0.01768388 | 0.92845739 | DOWN |
| NONMMUT029365.2 | 17:34950005-34952459 | 1172 | #NAME? | 0.0177694 | 0.92845739 | DOWN |
| NONMMUT071894.2 | X:13461090-13467719 | 2004 | #NAME? | 0.01776577 | 0.92845739 | DOWN |
| NONMMUT114623.1 | 4:8393516-8396725 | 1906 | #NAME? | 0.01771564 | 0.92845739 | DOWN |
| NONMMUT117995.1 | 5:20962642-20964029 | 1388 | -2.6332173 | 0.01759996 | 0.92845739 | DOWN |
| NONMMUT148703.1 | 4:131868943-131870117 | 1175 | -2.3935886 | 0.01775674 | 0.92845739 | DOWN |
| NONMMUT040646.2 | 2:155044339-155049081 | 3099 | #NAME? | 0.01781715 | 0.92961475 | DOWN |
| NONMMUT064678.2 | 8:13684646-13687361 | 1973 | 3.69745089 | 0.01788685 | 0.93191277 | UP |
| NONMMUT122919.1 | 6:117982077-117992295 | 4558 | Inf | 0.01803565 | 0.93831869 | UP |
| NONMMUT063126.2 | 7:106638537-106640676 | 2140 | Inf | 0.0180699 | 0.93875606 | UP |
| NONMMUT024877.2 | 15:97907657-97908869 | 1213 | -1.5784173 | 0.01821769 | 0.94376749 | DOWN |
| NONMMUT029397.2 | 17:35047125-35047772 | 474 | 3.78549217 | 0.01821835 | 0.94376749 | UP |
| ENSMUST00000209951 | 13:65241753-65250121 | 1597 | 3.15608913 | 0.01828398 | 0.94581813 | UP |
| ENSMUST00000051089 | 3:96269721-96279001 | 1511 | 2.51994739 | 0.0185392 | 0.95124619 | UP |
| ENSMUST00000202544 | 6:3342467-3346128 | 484 | 1.68872474 | 0.018455 | 0.95124619 | UP |
| MSTRG.5826.2 | 11:84925768-84935127 | 2655 | -1.5455258 | 0.01850867 | 0.95124619 | DOWN |
| NONMMUT052280.2 | 5:52190681-52197984 | 1500 | Inf | 0.01850144 | 0.95124619 | UP |
| NONMMUT100527.1 | 17:3250265-3251143 | 879 | -1.6331157 | 0.01854608 | 0.95124619 | DOWN |
| NONMMUT144311.1 | 16:30005272-30021430 | 5352 | Inf | 0.01841793 | 0.95124619 | UP |
| NONMMUT123657.1 | 6:39299667-39304060 | 4283 | -2.1195592 | 0.01876202 | 0.96096471 | DOWN |
| NONMMUT034385.2 | 19:29606196-29606674 | 479 | -1.518091 | 0.01888428 | 0.9648712 | DOWN |
| NONMMUT047160.2 | 4:46627089-46628865 | 1646 | Inf | 0.01889143 | 0.9648712 | UP |
| NONMMUT012371.2 | 11:106384034-106388088 | 4055 | -1.9703801 | 0.01900282 | 0.96708257 | DOWN |
| NONMMUT026105.2 | 16:28666677-28668152 | 1476 | 2.08587986 | 0.01900555 | 0.96708257 | UP |
| NONMMUT030108.2 | 17:56261237-56263448 | 2212 | 2.58037483 | 0.01901462 | 0.96708257 | UP |
| NONMMUT008425.2 | 11:5555056-5557753 | 2698 | 1.51326796 | 0.01910822 | 0.97048382 | UP |
| ENSMUST00000180987 | 14:20687236-20703012 | 1554 | -2.5789435 | 0.01929445 | 0.97672757 | DOWN |
| NONMMUT005410.2 | 10:42057056-42068603 | 895 | -3.0378126 | 0.01930117 | 0.97672757 | DOWN |
| NONMMUT018386.2 | 13:76579597-76581924 | 2328 | Inf | 0.01931185 | 0.97672757 | UP |
| NONMMUT115051.1 | 4:62737993-62739170 | 1178 | #NAME? | 0.01942008 | 0.98047688 | DOWN |
| NONMMUT115543.1 | 4:123221515-123222126 | 612 | 1.27153276 | 0.01943998 | 0.98047688 | UP |
| ENSMUST00000181206 | 16:11178176-11192292 | 2481 | 3.67716523 | 0.01969452 | 0.99164608 | UP |
| ENSMUST00000201762 | 6:3325742-3327855 | 2114 | 2.34894041 | 0.01971605 | 0.99164608 | UP |
| ENSMUST00000139163 | X:12762278-12821492 | 1662 | Inf | 0.02002577 | 0.9919564 | UP |
| ENSMUST00000160588 | 1:98031116-98035675 | 3469 | Inf | 0.01996825 | 0.9919564 | UP |
| ENSMUST00000186838 | 5:151367720-151428210 | 1543 | 2.21671419 | 0.02003588 | 0.9919564 | UP |
| ENSMUST00000210446 | 8:11874159-11894113 | 429 | -1.7289914 | 0.02005001 | 0.9919564 | DOWN |
| NONMMUT026095.2 | 16:28398137-28753225 | 475 | 3.52157636 | 0.0199176 | 0.9919564 | UP |
| NONMMUT034747.2 | 19:41969929-41971976 | 2048 | -3.4789004 | 0.01989654 | 0.9919564 | DOWN |
| NONMMUT051734.2 | 5:30876969-30877462 | 494 | 2.40217504 | 0.01990936 | 0.9919564 | UP |
| NONMMUT100135.1 | 16:75034041-75038075 | 4035 | 2.5409959 | 0.01982249 | 0.9919564 | UP |
| NONMMUT100155.1 | 16:75103104-75104277 | 1174 | 1.92456647 | 0.01983202 | 0.9919564 | UP |
| NONMMUT141194.1 | 11:119932773-119942734 | 6177 | Inf | 0.01994512 | 0.9919564 | UP |
| NONMMUT145272.1 | 17:84133783-84152748 | 1518 | Inf | 0.01996546 | 0.9919564 | UP |
| NONMMUT146235.1 | 2:26905639-26909315 | 1578 | #NAME? | 0.01988536 | 0.9919564 | DOWN |
| ENSMUST00000183087 | 18:61639653-61647472 | 588 | -2.5538259 | 0.02020054 | 0.99668829 | DOWN |
| NONMMUT068561.2 | 9:35079772-35087806 | 1632 | -1.1853425 | 0.02018387 | 0.99668829 | DOWN |
| ENSMUST00000066604 | 9:34918233-34929509 | 3799 | -3.0795307 | 0.03329576 | 1 | DOWN |
| ENSMUST00000114588 | 6:47943195-47948146 | 2733 | #NAME? | 0.03549141 | 1 | DOWN |
| ENSMUST00000118658 | 7:105690259-105690567 | 309 | -1.3401137 | 0.04157118 | 1 | DOWN |
| ENSMUST00000121358 | 2:177734300-177738757 | 3051 | Inf | 0.0429076 | 1 | UP |
| ENSMUST00000128026 | 2:153341157-153345810 | 1898 | #NAME? | 0.04445827 | 1 | DOWN |
| ENSMUST00000129932 | 12:91050369-91079907 | 219 | -1.8433645 | 0.03055347 | 1 | DOWN |
| ENSMUST00000130256 | 14:121248706-121253971 | 4715 | 3.31497373 | 0.02371624 | 1 | UP |
| ENSMUST00000131501 | 11:96916270-96953466 | 3542 | 2.50761103 | 0.04695482 | 1 | UP |
| ENSMUST00000136051 | 11:82779831-82781042 | 655 | -2.4034886 | 0.03800294 | 1 | DOWN |
| ENSMUST00000140273 | 11:120150562-120152341 | 1657 | #NAME? | 0.03886786 | 1 | DOWN |
| ENSMUST00000141810 | 7:120865509-120875298 | 2176 | -1.9806043 | 0.0333445 | 1 | DOWN |
| ENSMUST00000144178 | 2:129297370-129307826 | 1781 | #NAME? | 0.02985056 | 1 | DOWN |
| ENSMUST00000145206 | 11:113043895-113201822 | 1802 | #NAME? | 0.02712543 | 1 | DOWN |
| ENSMUST00000147848 | 16:91044656-91056677 | 1252 | Inf | 0.03897225 | 1 | UP |
| ENSMUST00000148587 | 2:117857291-118111202 | 3469 | -2.2230541 | 0.03052129 | 1 | DOWN |
| ENSMUST00000149619 | 2:25143817-25145323 | 659 | -2.8732061 | 0.04539572 | 1 | DOWN |
| ENSMUST00000154354 | 11:59199836-59202385 | 1360 | 2.02609836 | 0.02783082 | 1 | UP |
| ENSMUST00000154810 | X:169975043-169978917 | 2380 | 3.54884752 | 0.0429446 | 1 | UP |
| ENSMUST00000157018 | 8:110805638-110807477 | 731 | 1.550041 | 0.02182525 | 1 | UP |
| ENSMUST00000158164 | 12:105031075-105031349 | 275 | 4.38038169 | 0.02987485 | 1 | UP |
| ENSMUST00000163836 | 2:26138383-26138802 | 420 | -1.96509 | 0.02544503 | 1 | DOWN |
| ENSMUST00000167221 | 14:51825720-51826293 | 574 | -1.7897651 | 0.03571857 | 1 | DOWN |
| ENSMUST00000178227 | 5:15516489-15656679 | 1068 | -3.1438494 | 0.02102117 | 1 | DOWN |
| ENSMUST00000180563 | 9:88521052-88522890 | 1004 | 2.50794869 | 0.04319404 | 1 | UP |
| ENSMUST00000181291 | 17:23743234-23750915 | 1798 | 1.31254226 | 0.04148 | 1 | UP |
| ENSMUST00000181296 | 9:41588494-41592737 | 4244 | 3.45313387 | 0.04667187 | 1 | UP |
| ENSMUST00000181303 | 3:51562471-51567105 | 1694 | 1.89326689 | 0.04165269 | 1 | UP |
| ENSMUST00000181453 | 18:34751809-34756662 | 2400 | #NAME? | 0.03886576 | 1 | DOWN |
| ENSMUST00000181713 | 18:32153476-32160427 | 1418 | #NAME? | 0.04446588 | 1 | DOWN |
| ENSMUST00000181960 | 16:21794362-21809040 | 574 | -2.2660982 | 0.02062513 | 1 | DOWN |
| ENSMUST00000182286 | 14:61635227-61682314 | 666 | 1.47116632 | 0.0419045 | 1 | UP |
| ENSMUST00000183365 | 8:106587143-106594820 | 1341 | 2.3769039 | 0.04522604 | 1 | UP |
| ENSMUST00000184855 | 7:73544306-73558248 | 409 | #NAME? | 0.02515713 | 1 | DOWN |
| ENSMUST00000186535 | 16:50725625-50732728 | 2896 | 1.36881874 | 0.04950811 | 1 | UP |
| ENSMUST00000187034 | 2:24442849-24445264 | 2416 | -1.8295924 | 0.02046346 | 1 | DOWN |
| ENSMUST00000187757 | 3:106484070-106485848 | 1779 | 1.21036501 | 0.02134437 | 1 | UP |
| ENSMUST00000191170 | 6:82879421-82881832 | 324 | 2.02560605 | 0.03322012 | 1 | UP |
| ENSMUST00000192163 | 14:69502600-69504096 | 1497 | 2.46761828 | 0.02828587 | 1 | UP |
| ENSMUST00000193280 | 17:31532291-31535460 | 3170 | Inf | 0.0317893 | 1 | UP |
| ENSMUST00000194758 | 9:76067449-76070946 | 3498 | Inf | 0.03173205 | 1 | UP |
| ENSMUST00000195134 | 1:40771385-40785343 | 3261 | -1.1000805 | 0.04165135 | 1 | DOWN |
| ENSMUST00000195772 | 5:32130283-32133171 | 2889 | 1.91518923 | 0.03033016 | 1 | UP |
| ENSMUST00000196372 | 5:5170394-5175152 | 4759 | 2.40958387 | 0.04894439 | 1 | UP |
| ENSMUST00000197239 | 5:14027894-14029765 | 1872 | 1.98715227 | 0.03664103 | 1 | UP |
| ENSMUST00000197277 | 5:15538002-15605066 | 512 | #NAME? | 0.03056743 | 1 | DOWN |
| ENSMUST00000198817 | 5:5309002-5311623 | 2622 | Inf | 0.0487421 | 1 | UP |
| ENSMUST00000199664 | 5:144223725-144225520 | 1796 | #NAME? | 0.03260471 | 1 | DOWN |
| ENSMUST00000200956 | 5:31560812-31569072 | 6548 | 2.19096502 | 0.04820241 | 1 | UP |
| ENSMUST00000203383 | 6:66894652-66895456 | 805 | Inf | 0.04826592 | 1 | UP |
| ENSMUST00000205400 | 7:55973519-55974842 | 1324 | 2.72341096 | 0.02582904 | 1 | UP |
| ENSMUST00000205547 | 6:91522409-91532379 | 600 | -3.9605619 | 0.0283542 | 1 | DOWN |
| ENSMUST00000205735 | 7:80404269-80406577 | 2309 | 3.27847958 | 0.02442928 | 1 | UP |
| ENSMUST00000206867 | 6:72013896-72042528 | 2631 | 1.26766182 | 0.03825685 | 1 | UP |
| ENSMUST00000207452 | 6:82896867-82898100 | 918 | 2.3874897 | 0.03988966 | 1 | UP |
| ENSMUST00000207806 | 7:121062852-121063959 | 1108 | 2.77188064 | 0.03659338 | 1 | UP |
| ENSMUST00000207838 | 7:131401236-131402796 | 1561 | 1.45893302 | 0.0344469 | 1 | UP |
| ENSMUST00000208872 | 6:82896867-82898100 | 958 | #NAME? | 0.04948883 | 1 | DOWN |
| ENSMUST00000209090 | 7:98904268-98907902 | 2531 | #NAME? | 0.02606666 | 1 | DOWN |
| ENSMUST00000209449 | 16:21794342-21809026 | 580 | -2.345669 | 0.0443341 | 1 | DOWN |
| ENSMUST00000209918 | 8:88560033-88562949 | 2917 | 3.70752488 | 0.02103308 | 1 | UP |
| ENSMUST00000210161 | 8:77282861-77286207 | 1637 | #NAME? | 0.04016303 | 1 | DOWN |
| ENSMUST00000210802 | 9:43243599-43267360 | 2761 | -1.1813434 | 0.03161897 | 1 | DOWN |
| ENSMUST00000211025 | 2:144173685-144191246 | 1262 | Inf | 0.02120294 | 1 | UP |
| ENSMUST00000213810 | 9:122019528-122051463 | 9813 | -2.0428762 | 0.02725454 | 1 | DOWN |
| ENSMUST00000213976 | 9:20385234-20387728 | 2495 | #NAME? | 0.02175949 | 1 | DOWN |
| ENSMUST00000214235 | 9:60334142-60334968 | 364 | 1.19262445 | 0.03109494 | 1 | UP |
| ENSMUST00000215481 | 9:120009805-120011448 | 566 | -1.9710654 | 0.02181544 | 1 | DOWN |
| ENSMUST00000216590 | 9:13431361-13432751 | 1391 | 2.64945931 | 0.03846677 | 1 | UP |
| ENSMUST00000216597 | 9:42142087-42146506 | 2075 | 2.84900767 | 0.0459348 | 1 | UP |
| ENSMUST00000220364 | 10:121506623-121526101 | 778 | #NAME? | 0.02769736 | 1 | DOWN |
| ENSMUST00000222184 | 13:53028918-53035090 | 2973 | -1.3168436 | 0.02410374 | 1 | DOWN |
| ENSMUST00000227042 | 14:32301924-32308895 | 1870 | Inf | 0.02608151 | 1 | UP |
| ENSMUST00000227369 | 14:61492939-61527389 | 3076 | #NAME? | 0.03136257 | 1 | DOWN |
| MSTRG.36585.3 | 9:35078975-35083528 | 1371 | -1.1714629 | 0.02221804 | 1 | DOWN |
| MSTRG.36586.1 | 9:35076432-35085702 | 689 | -1.230742 | 0.03458192 | 1 | DOWN |
| MSTRG.38645.2 | CHR_MG132_PATCH:124461563-124462928 | 970 | 1.76909736 | 0.03424902 | 1 | UP |
| MSTRG.39373.1 | X:94911206-94913983 | 708 | 2.10181328 | 0.04972129 | 1 | UP |
| NONMMUT000698.2 | 1:39956758-39960277 | 3520 | 1.6226727 | 0.02352991 | 1 | UP |
| NONMMUT000701.2 | 1:40025391-40026297 | 907 | 1.62411152 | 0.03278415 | 1 | UP |
| NONMMUT000800.2 | 1:44572905-44575357 | 2453 | 1.7632226 | 0.04359794 | 1 | UP |
| NONMMUT000936.2 | 1:54475358-54477598 | 2241 | 1.23901218 | 0.03202741 | 1 | UP |
| NONMMUT001008.2 | 1:57803219-57806637 | 3419 | 2.20810042 | 0.03351055 | 1 | UP |
| NONMMUT001438.2 | 1:72196394-72198326 | 791 | Inf | 0.02117823 | 1 | UP |
| NONMMUT001511.2 | 1:73911887-73913701 | 1815 | 2.54936285 | 0.02261175 | 1 | UP |
| NONMMUT002444.2 | 1:126202622-126204235 | 1614 | 1.25357529 | 0.03940629 | 1 | UP |
| NONMMUT002743.2 | 1:135438848-135440967 | 2120 | 1.39816245 | 0.04556994 | 1 | UP |
| NONMMUT002978.2 | 1:139174123-139176037 | 1915 | -2.0397886 | 0.03300784 | 1 | DOWN |
| NONMMUT003561.2 | 1:164491148-164495297 | 3233 | Inf | 0.03840357 | 1 | UP |
| NONMMUT003842.2 | 1:172388366-172391018 | 1296 | Inf | 0.02780841 | 1 | UP |
| NONMMUT003884.2 | 1:174225164-174228491 | 3328 | #NAME? | 0.04191837 | 1 | DOWN |
| NONMMUT003914.2 | 1:176834624-176836133 | 1510 | Inf | 0.02284032 | 1 | UP |
| NONMMUT003946.2 | 1:178143597-178147220 | 847 | #NAME? | 0.03528703 | 1 | DOWN |
| NONMMUT004118.2 | 1:182124781-182125628 | 848 | 2.30584295 | 0.04070743 | 1 | UP |
| NONMMUT004252.2 | 1:187035466-187038508 | 2956 | -1.5493148 | 0.03357422 | 1 | DOWN |
| NONMMUT004449.2 | 1:193279412-193281077 | 1365 | 3.67045252 | 0.02067536 | 1 | UP |
| NONMMUT004592.2 | 10:3554159-3557637 | 3479 | -1.7743998 | 0.03000273 | 1 | DOWN |
| NONMMUT004849.2 | 10:18585490-18587216 | 1727 | -2.8226476 | 0.02680477 | 1 | DOWN |
| NONMMUT004932.2 | 10:20680143-20682859 | 2717 | Inf | 0.04879962 | 1 | UP |
| NONMMUT004971.2 | 10:22258546-22302835 | 2223 | 1.48616506 | 0.04121229 | 1 | UP |
| NONMMUT005322.2 | 10:39653215-39731993 | 1897 | 3.58301244 | 0.02056372 | 1 | UP |
| NONMMUT005406.2 | 10:41907655-41908421 | 767 | -2.4735107 | 0.03297296 | 1 | DOWN |
| NONMMUT006381.2 | 10:80053228-80053943 | 710 | 2.46660129 | 0.02966402 | 1 | UP |
| NONMMUT006435.2 | 10:80826656-80829290 | 1047 | -1.5028527 | 0.0474742 | 1 | DOWN |
| NONMMUT006539.2 | 10:82613619-82616814 | 2368 | #NAME? | 0.02492501 | 1 | DOWN |
| NONMMUT006741.2 | 10:88469522-88470948 | 1427 | -2.8756554 | 0.0287586 | 1 | DOWN |
| NONMMUT007086.2 | 10:99233067-99239176 | 6016 | #NAME? | 0.02120138 | 1 | DOWN |
| NONMMUT007558.2 | 10:117669344-117671243 | 1900 | -1.2609126 | 0.02614667 | 1 | DOWN |
| NONMMUT008151.2 | 10:128441123-128441437 | 315 | 2.66182686 | 0.02237204 | 1 | UP |
| NONMMUT008216.2 | 11:3124050-3132427 | 2597 | 1.72983659 | 0.02702795 | 1 | UP |
| NONMMUT009157.2 | 11:31832458-31838129 | 1006 | -2.8097285 | 0.04318001 | 1 | DOWN |
| NONMMUT009232.2 | 11:34095108-34097111 | 2004 | 2.22014531 | 0.02211091 | 1 | UP |
| NONMMUT009654.2 | 11:51404487-51407441 | 2955 | #NAME? | 0.046145 | 1 | DOWN |
| NONMMUT009671.2 | 11:51756489-51756874 | 386 | -3.5482927 | 0.03916668 | 1 | DOWN |
| NONMMUT009972.2 | 11:59505225-59512258 | 3528 | -2.6137 | 0.03496315 | 1 | DOWN |
| NONMMUT011032.2 | 11:80508500-80508885 | 386 | -2.0345094 | 0.02077429 | 1 | DOWN |
| NONMMUT011090.2 | 11:82951881-82955330 | 3450 | 1.51104999 | 0.04069707 | 1 | UP |
| NONMMUT011110.2 | 11:83291688-83293344 | 1517 | -2.0550072 | 0.03202276 | 1 | DOWN |
| NONMMUT011659.2 | 11:95768887-95770156 | 1270 | -1.7527665 | 0.04214989 | 1 | DOWN |
| NONMMUT011690.2 | 11:96132740-96133498 | 602 | #NAME? | 0.04588564 | 1 | DOWN |
| NONMMUT011946.2 | 11:99051575-99052641 | 1067 | 2.22200622 | 0.02927014 | 1 | UP |
| NONMMUT012438.2 | 11:107218136-107220553 | 2418 | 1.91192073 | 0.02847955 | 1 | UP |
| NONMMUT012700.2 | 11:115364860-115367706 | 2847 | -1.2525259 | 0.04947107 | 1 | DOWN |
| NONMMUT012733.2 | 11:115580232-115581590 | 1359 | -2.0189126 | 0.02558512 | 1 | DOWN |
| NONMMUT012763.2 | 11:115899949-115906256 | 1005 | 2.23386894 | 0.02977121 | 1 | UP |
| NONMMUT013400.2 | 12:9580596-9581499 | 904 | Inf | 0.04818043 | 1 | UP |
| NONMMUT013700.2 | 12:24888379-24890611 | 2233 | 1.52761924 | 0.02991369 | 1 | UP |
| NONMMUT013830.2 | 12:31472584-31473917 | 1334 | -1.2536219 | 0.02594796 | 1 | DOWN |
| NONMMUT013914.2 | 12:34702812-34704911 | 2100 | 1.75956711 | 0.02979658 | 1 | UP |
| NONMMUT014141.2 | 12:51377580-51378588 | 1009 | -2.882697 | 0.04271452 | 1 | DOWN |
| NONMMUT014342.2 | 12:58214532-58216036 | 1451 | -3.9285542 | 0.03378943 | 1 | DOWN |
| NONMMUT014681.2 | 12:75450015-75453260 | 3246 | -1.6578773 | 0.02249486 | 1 | DOWN |
| NONMMUT014807.2 | 12:80114342-80132844 | 11049 | -2.562583 | 0.0393501 | 1 | DOWN |
| NONMMUT014810.2 | 12:80114342-80132172 | 11144 | -1.8586002 | 0.02394714 | 1 | DOWN |
| NONMMUT014831.2 | 12:80501678-80503185 | 1508 | 3.43072241 | 0.03943276 | 1 | UP |
| NONMMUT015126.2 | 12:87194513-87196849 | 2337 | #NAME? | 0.03792871 | 1 | DOWN |
| NONMMUT015256.2 | 12:95782009-95783607 | 1599 | 3.32273447 | 0.03491214 | 1 | UP |
| NONMMUT016138.2 | 12:116770940-116773281 | 2342 | -1.8726245 | 0.04485806 | 1 | DOWN |
| NONMMUT016256.2 | 13:5855510-5857657 | 1249 | Inf | 0.03727948 | 1 | UP |
| NONMMUT016478.2 | 13:17022937-17025987 | 3051 | Inf | 0.0432421 | 1 | UP |
| NONMMUT017599.2 | 13:54106596-54108065 | 1470 | -1.4245982 | 0.03219725 | 1 | DOWN |
| NONMMUT017741.2 | 13:58114897-58116158 | 1065 | 2.47722918 | 0.04213189 | 1 | UP |
| NONMMUT018132.2 | 13:67817820-67831264 | 3388 | 3.01321731 | 0.03786645 | 1 | UP |
| NONMMUT019941.2 | 14:22688029-22689423 | 1395 | Inf | 0.02281301 | 1 | UP |
| NONMMUT020640.2 | 14:47293612-47296098 | 2487 | -2.0787142 | 0.04513324 | 1 | DOWN |
| NONMMUT020697.2 | 14:49006256-49009827 | 3572 | 1.48707996 | 0.03824688 | 1 | UP |
| NONMMUT020793.2 | 14:52198151-52198600 | 450 | -1.6690375 | 0.03962858 | 1 | DOWN |
| NONMMUT021209.2 | 14:62607177-62608485 | 1309 | 2.55774447 | 0.0213227 | 1 | UP |
| NONMMUT021639.2 | 14:75192138-75193203 | 1066 | 2.06824682 | 0.04704826 | 1 | UP |
| NONMMUT021690.2 | 14:76514513-76515755 | 1066 | #NAME? | 0.03818634 | 1 | DOWN |
| NONMMUT021994.2 | 14:99916758-99920143 | 3386 | #NAME? | 0.04588879 | 1 | DOWN |
| NONMMUT022055.2 | 14:103012908-103024444 | 2276 | Inf | 0.03175276 | 1 | UP |
| NONMMUT023005.2 | 15:31290864-31292351 | 1488 | Inf | 0.04880198 | 1 | UP |
| NONMMUT023065.2 | 15:34127980-34130596 | 2617 | -2.0707064 | 0.02684713 | 1 | DOWN |
| NONMMUT023439.2 | 15:50793846-50796368 | 2523 | 3.49124603 | 0.02823029 | 1 | UP |
| NONMMUT023441.2 | 15:50874604-50877896 | 2963 | Inf | 0.04326482 | 1 | UP |
| NONMMUT023442.2 | 15:50880138-50883076 | 2939 | Inf | 0.02120067 | 1 | UP |
| NONMMUT024129.2 | 15:78417318-78418317 | 579 | #NAME? | 0.02175895 | 1 | DOWN |
| NONMMUT024339.2 | 15:81835238-81839865 | 2286 | -2.4036726 | 0.02983416 | 1 | DOWN |
| NONMMUT024526.2 | 15:86184671-86186341 | 1671 | 1.85257902 | 0.02826814 | 1 | UP |
| NONMMUT025163.2 | 15:103158076-103159918 | 1843 | -2.137373 | 0.02908893 | 1 | DOWN |
| NONMMUT025494.2 | 16:11143906-11144315 | 410 | -1.1544631 | 0.02671285 | 1 | DOWN |
| NONMMUT026107.2 | 16:28681074-28683618 | 2545 | 1.86422902 | 0.02198639 | 1 | UP |
| NONMMUT026108.2 | 16:28752633-28753339 | 538 | 2.05696895 | 0.03331924 | 1 | UP |
| NONMMUT026120.2 | 16:29809389-29812856 | 3468 | Inf | 0.03727754 | 1 | UP |
| NONMMUT026141.2 | 16:30068461-30069007 | 308 | Inf | 0.02982573 | 1 | UP |
| NONMMUT026285.2 | 16:32392586-32393746 | 1161 | 2.79969079 | 0.03802259 | 1 | UP |
| NONMMUT026451.2 | 16:36184212-36188108 | 1859 | Inf | 0.02546683 | 1 | UP |
| NONMMUT026799.2 | 16:45746244-45747457 | 1214 | 1.98498394 | 0.03079179 | 1 | UP |
| NONMMUT026800.2 | 16:45763671-45765821 | 2151 | 2.20133544 | 0.04486987 | 1 | UP |
| NONMMUT027000.2 | 16:57168868-57170364 | 1497 | 1.76196161 | 0.03438063 | 1 | UP |
| NONMMUT027268.2 | 16:74997909-75002289 | 4381 | 3.27413268 | 0.03150087 | 1 | UP |
| NONMMUT027271.2 | 16:75030852-75032184 | 1333 | 3.67783346 | 0.04609257 | 1 | UP |
| NONMMUT027273.2 | 16:75082198-75085753 | 3556 | 2.3866326 | 0.04600928 | 1 | UP |
| NONMMUT027426.2 | 16:78541246-78543560 | 2315 | 2.46236267 | 0.0245477 | 1 | UP |
| NONMMUT027946.2 | 17:3081406-3084531 | 1871 | 4.3801861 | 0.02770577 | 1 | UP |
| NONMMUT028088.2 | 17:6798390-6806631 | 1360 | -2.5033564 | 0.04291703 | 1 | DOWN |
| NONMMUT028117.2 | 17:8043852-8046868 | 2916 | 2.65336753 | 0.02642681 | 1 | UP |
| NONMMUT028182.2 | 17:10206758-10209206 | 2449 | 2.17426316 | 0.02343253 | 1 | UP |
| NONMMUT028468.2 | 17:20988851-20989722 | 872 | Inf | 0.03896504 | 1 | UP |
| NONMMUT028798.2 | 17:26444856-26446313 | 1458 | 2.20221263 | 0.04325829 | 1 | UP |
| NONMMUT029134.2 | 17:32374296-32375947 | 1652 | 1.83783241 | 0.04500559 | 1 | UP |
| NONMMUT029718.2 | 17:43455325-43456591 | 1267 | 1.21381805 | 0.04589309 | 1 | UP |
| NONMMUT029749.2 | 17:44117792-44121012 | 1607 | Inf | 0.03631635 | 1 | UP |
| NONMMUT029885.2 | 17:47527968-47532454 | 4487 | 1.58774204 | 0.02605547 | 1 | UP |
| NONMMUT030322.2 | 17:66519790-66524521 | 925 | #NAME? | 0.03033372 | 1 | DOWN |
| NONMMUT030324.2 | 17:66523167-66524521 | 1013 | #NAME? | 0.02120192 | 1 | DOWN |
| NONMMUT030518.2 | 17:74055420-74080280 | 1718 | Inf | 0.03632539 | 1 | UP |
| NONMMUT030525.2 | 17:74055420-74080280 | 1707 | Inf | 0.0363216 | 1 | UP |
| NONMMUT030904.2 | 17:84134081-84136736 | 1107 | 1.53707184 | 0.03514523 | 1 | UP |
| NONMMUT031155.2 | 17:94752465-94775167 | 1380 | -3.0112817 | 0.02515886 | 1 | DOWN |
| NONMMUT031441.2 | 18:10150976-10152891 | 1916 | -2.3652467 | 0.04071353 | 1 | DOWN |
| NONMMUT032394.2 | 18:54917670-54919343 | 1674 | 1.33422131 | 0.0401019 | 1 | UP |
| NONMMUT032576.2 | 18:61458256-61459511 | 965 | -2.5489321 | 0.02188085 | 1 | DOWN |
| NONMMUT033031.2 | 18:74644116-74659060 | 1979 | -1.8674597 | 0.02232696 | 1 | DOWN |
| NONMMUT033083.2 | 18:75363727-75364932 | 1107 | 2.80962736 | 0.02910149 | 1 | UP |
| NONMMUT033955.2 | 19:12448539-12454575 | 3435 | 2.41255978 | 0.03536789 | 1 | UP |
| NONMMUT034344.2 | 19:29012844-29015495 | 2652 | 2.36169644 | 0.02316585 | 1 | UP |
| NONMMUT034384.2 | 19:29604096-29605029 | 934 | -2.415801 | 0.02321626 | 1 | DOWN |
| NONMMUT034752.2 | 19:42056535-42058543 | 2009 | 2.04306339 | 0.02872337 | 1 | UP |
| NONMMUT035080.2 | 19:53461646-53464796 | 1958 | 2.0219914 | 0.03291468 | 1 | UP |
| NONMMUT035138.2 | 19:55526590-55529975 | 3386 | -1.0163123 | 0.03606574 | 1 | DOWN |
| NONMMUT035139.2 | 19:55554873-55556174 | 1302 | -1.0940885 | 0.04261528 | 1 | DOWN |
| NONMMUT035490.2 | 2:5989693-5992420 | 2728 | -2.4265638 | 0.02452729 | 1 | DOWN |
| NONMMUT035523.2 | 2:6882588-6884928 | 2341 | Inf | 0.03838198 | 1 | UP |
| NONMMUT036285.2 | 2:26059010-26060064 | 1055 | -2.1198053 | 0.0254585 | 1 | DOWN |
| NONMMUT036479.2 | 2:28841111-28841986 | 876 | Inf | 0.03117564 | 1 | UP |
| NONMMUT036531.2 | 2:29911363-29912070 | 708 | -1.534218 | 0.04242998 | 1 | DOWN |
| NONMMUT036555.2 | 2:30193353-30194921 | 1569 | -1.5476778 | 0.04674902 | 1 | DOWN |
| NONMMUT036760.2 | 2:33133461-33135615 | 2155 | 2.21843841 | 0.03894385 | 1 | UP |
| NONMMUT037133.2 | 2:45277039-45282909 | 3054 | 2.75826151 | 0.03546687 | 1 | UP |
| NONMMUT037179.2 | 2:48949510-49245737 | 900 | 2.92049872 | 0.03413093 | 1 | UP |
| NONMMUT037510.2 | 2:60851978-60854440 | 2463 | 1.50970122 | 0.04329414 | 1 | UP |
| NONMMUT037544.2 | 2:61711720-61712350 | 631 | -1.9671787 | 0.03432316 | 1 | DOWN |
| NONMMUT037891.2 | 2:72979929-72981600 | 1672 | 1.53634696 | 0.04121422 | 1 | UP |
| NONMMUT038062.2 | 2:76587266-76590610 | 3345 | -1.6131745 | 0.04981902 | 1 | DOWN |
| NONMMUT038293.2 | 2:84838852-84840038 | 1187 | -3.0551068 | 0.0337031 | 1 | DOWN |
| NONMMUT038834.2 | 2:103093052-103097926 | 1708 | -3.614658 | 0.04604168 | 1 | DOWN |
| NONMMUT038953.2 | 2:105172658-105173612 | 955 | 3.62442789 | 0.02991482 | 1 | UP |
| NONMMUT039605.2 | 2:125124033-125126211 | 2179 | Inf | 0.02276918 | 1 | UP |
| NONMMUT039652.2 | 2:126670990-126672197 | 1208 | -1.5845963 | 0.03767681 | 1 | DOWN |
| NONMMUT040118.2 | 2:136904602-136909170 | 4569 | -1.3453815 | 0.02270831 | 1 | DOWN |
| NONMMUT040545.2 | 2:152727708-152730799 | 3092 | #NAME? | 0.03723147 | 1 | DOWN |
| NONMMUT040967.2 | 2:162634501-162636506 | 2006 | -2.1965181 | 0.04862232 | 1 | DOWN |
| NONMMUT041155.2 | 2:165874877-165884629 | 982 | -1.640911 | 0.03528423 | 1 | DOWN |
| NONMMUT041406.2 | 2:169624070-169626353 | 2284 | Inf | 0.04131256 | 1 | UP |
| NONMMUT041559.2 | 2:173244839-173247215 | 1031 | 1.38890719 | 0.02386298 | 1 | UP |
| NONMMUT041560.2 | 2:173259154-173262972 | 3819 | 1.19340668 | 0.02055945 | 1 | UP |
| NONMMUT041764.2 | 2:178411231-178412891 | 1661 | 1.25054995 | 0.03069928 | 1 | UP |
| NONMMUT043649.2 | 3:84160441-84164031 | 3591 | -1.8776301 | 0.02571715 | 1 | DOWN |
| NONMMUT046181.2 | 4:11175964-11191274 | 4019 | 2.23963763 | 0.0284646 | 1 | UP |
| NONMMUT046616.2 | 4:33026966-33031323 | 3966 | -2.1373264 | 0.03239843 | 1 | DOWN |
| NONMMUT046784.2 | 4:40720154-40722413 | 2260 | 1.65198392 | 0.03464293 | 1 | UP |
| NONMMUT047054.2 | 4:44526388-44529159 | 2772 | -2.2658438 | 0.02942236 | 1 | DOWN |
| NONMMUT047279.2 | 4:53133624-53137088 | 3465 | 2.59156552 | 0.02384934 | 1 | UP |
| NONMMUT047443.2 | 4:57868858-57870279 | 1422 | -1.6145713 | 0.02953705 | 1 | DOWN |
| NONMMUT048006.2 | 4:87973906-87974525 | 537 | Inf | 0.0223134 | 1 | UP |
| NONMMUT048194.2 | 4:96022180-96025388 | 3209 | 1.96607072 | 0.04544443 | 1 | UP |
| NONMMUT048239.2 | 4:98115994-98118843 | 2850 | 1.91867895 | 0.03710754 | 1 | UP |
| NONMMUT048683.2 | 4:109664193-109671594 | 2485 | #NAME? | 0.0478722 | 1 | DOWN |
| NONMMUT049311.2 | 4:123410089-123411911 | 1823 | 2.5558998 | 0.02757325 | 1 | UP |
| NONMMUT050057.2 | 4:135494747-135497987 | 3241 | -1.5492615 | 0.02869102 | 1 | DOWN |
| NONMMUT050358.2 | 4:141677555-141678460 | 851 | #NAME? | 0.03117985 | 1 | DOWN |
| NONMMUT050504.2 | 4:145834946-145845540 | 4411 | 3.2369207 | 0.02930063 | 1 | UP |
| NONMMUT052433.2 | 5:64481731-64490166 | 5838 | #NAME? | 0.02667688 | 1 | DOWN |
| NONMMUT052722.2 | 5:73377933-73380146 | 808 | #NAME? | 0.02799365 | 1 | DOWN |
| NONMMUT053008.2 | 5:88650497-88651414 | 918 | -1.9057227 | 0.04364041 | 1 | DOWN |
| NONMMUT053028.2 | 5:89238134-89239647 | 1514 | -1.8445601 | 0.04531162 | 1 | DOWN |
| NONMMUT053046.2 | 5:90262434-90266684 | 4251 | -2.1078934 | 0.02671008 | 1 | DOWN |
| NONMMUT053460.2 | 5:104571656-104645041 | 7807 | -1.5021647 | 0.03255252 | 1 | DOWN |
| NONMMUT053574.2 | 5:107390018-107395141 | 3909 | #NAME? | 0.03680386 | 1 | DOWN |
| NONMMUT053625.2 | 5:108107750-108109217 | 1468 | -1.6520241 | 0.03886833 | 1 | DOWN |
| NONMMUT053653.2 | 5:108540397-108544379 | 3983 | -2.2623739 | 0.03901685 | 1 | DOWN |
| NONMMUT054394.2 | 5:123133728-123139395 | 5565 | -4.0550428 | 0.02198774 | 1 | DOWN |
| NONMMUT054397.2 | 5:123133733-123142055 | 2410 | -2.6761944 | 0.04755498 | 1 | DOWN |
| NONMMUT054401.2 | 5:123133739-123136147 | 2213 | Inf | 0.04400587 | 1 | UP |
| NONMMUT054835.2 | 5:135642435-135656045 | 1917 | #NAME? | 0.03491052 | 1 | DOWN |
| NONMMUT055527.2 | 5:151085402-151087271 | 1870 | -1.4560779 | 0.0402462 | 1 | DOWN |
| NONMMUT055600.2 | 6:5164485-5165659 | 1175 | 1.97477374 | 0.04576359 | 1 | UP |
| NONMMUT055750.2 | 6:11924986-11926439 | 1454 | 3.64976064 | 0.04837025 | 1 | UP |
| NONMMUT056239.2 | 6:32144561-32147748 | 3188 | 3.13800194 | 0.04723763 | 1 | UP |
| NONMMUT056408.2 | 6:37442178-37448574 | 3161 | -2.5377353 | 0.03439035 | 1 | DOWN |
| NONMMUT056519.2 | 6:39265381-39269872 | 1044 | -2.8727789 | 0.04139109 | 1 | DOWN |
| NONMMUT057017.2 | 6:53639486-53644842 | 5357 | 3.14582161 | 0.02356756 | 1 | UP |
| NONMMUT057444.2 | 6:72629288-72637717 | 1073 | 2.00716322 | 0.04606097 | 1 | UP |
| NONMMUT058016.2 | 6:92370919-92374635 | 3717 | Inf | 0.02780216 | 1 | UP |
| NONMMUT058167.2 | 6:98928862-98930077 | 1216 | 1.78446849 | 0.03230904 | 1 | UP |
| NONMMUT058613.2 | 6:119571858-119574335 | 2478 | -1.2914633 | 0.03805538 | 1 | DOWN |
| NONMMUT059076.2 | 6:129203248-129205769 | 1555 | 1.69038116 | 0.0465748 | 1 | UP |
| NONMMUT059085.2 | 6:129227732-129238444 | 749 | Inf | 0.02547153 | 1 | UP |
| NONMMUT059308.2 | 6:140654256-140655301 | 1046 | -1.9750512 | 0.0357525 | 1 | DOWN |
| NONMMUT059314.2 | 6:140777207-140779662 | 2456 | Inf | 0.0389703 | 1 | UP |
| NONMMUT059573.2 | 6:148232462-148236189 | 3728 | 2.44673861 | 0.02823429 | 1 | UP |
| NONMMUT060284.2 | 7:25418218-25420706 | 2489 | #NAME? | 0.03032358 | 1 | DOWN |
| NONMMUT060869.2 | 7:39551952-39580814 | 16799 | -3.3690997 | 0.02546025 | 1 | DOWN |
| NONMMUT061280.2 | 7:48755541-48778187 | 577 | #NAME? | 0.0271206 | 1 | DOWN |
| NONMMUT061325.2 | 7:51856967-51860049 | 3083 | 1.8133195 | 0.03319628 | 1 | UP |
| NONMMUT061330.2 | 7:51997218-52005691 | 3125 | Inf | 0.02528341 | 1 | UP |
| NONMMUT061371.2 | 7:55976576-55980620 | 1836 | -2.9974706 | 0.03204727 | 1 | DOWN |
| NONMMUT061867.2 | 7:64301415-64303172 | 1758 | -1.8225665 | 0.03042889 | 1 | DOWN |
| NONMMUT062245.2 | 7:75376672-75377743 | 955 | 1.91305919 | 0.02347955 | 1 | UP |
| NONMMUT062431.2 | 7:80978902-80981477 | 1984 | -1.9705521 | 0.02794037 | 1 | DOWN |
| NONMMUT062457.2 | 7:81523550-81531606 | 7841 | 2.29826708 | 0.02737961 | 1 | UP |
| NONMMUT062538.2 | 7:83866777-83868776 | 2000 | #NAME? | 0.03056727 | 1 | DOWN |
| NONMMUT063065.2 | 7:104218813-104219865 | 1053 | 1.31098969 | 0.02453112 | 1 | UP |
| NONMMUT063301.2 | 7:113568806-113570879 | 2074 | -1.3298396 | 0.03825451 | 1 | DOWN |
| NONMMUT063606.2 | 7:125707917-125709369 | 1453 | 1.66335748 | 0.02712159 | 1 | UP |
| NONMMUT063782.2 | 7:127978141-127979654 | 1514 | -1.0455182 | 0.04433615 | 1 | DOWN |
| NONMMUT063879.2 | 7:129789419-129793025 | 1552 | Inf | 0.025284 | 1 | UP |
| NONMMUT066322.2 | 8:83085241-83087756 | 2516 | -2.3762089 | 0.04031788 | 1 | DOWN |
| NONMMUT066351.2 | 8:83734903-83736129 | 1227 | 2.740121 | 0.03166918 | 1 | UP |
| NONMMUT067011.2 | 8:106210144-106212308 | 2165 | 2.10110253 | 0.04233076 | 1 | UP |
| NONMMUT067238.2 | 8:111662265-111665377 | 3113 | 1.50860516 | 0.03674642 | 1 | UP |
| NONMMUT067520.2 | 8:122429347-122432074 | 2728 | 1.29398926 | 0.04637725 | 1 | UP |
| NONMMUT067713.2 | 8:125027371-125030421 | 3051 | 1.81849412 | 0.03241959 | 1 | UP |
| NONMMUT068214.2 | 9:19648334-19649730 | 1397 | -1.6104444 | 0.04291288 | 1 | DOWN |
| NONMMUT068356.2 | 9:23319959-23321500 | 1542 | 2.19724935 | 0.0331439 | 1 | UP |
| NONMMUT068781.2 | 9:43264982-43280062 | 714 | -2.3963739 | 0.03694553 | 1 | DOWN |
| NONMMUT068904.2 | 9:45054823-45055344 | 522 | 2.13915236 | 0.03420293 | 1 | UP |
| NONMMUT068909.2 | 9:45128678-45130067 | 1390 | -3.6570927 | 0.02935108 | 1 | DOWN |
| NONMMUT068994.2 | 9:47800530-47802439 | 1910 | 3.01600318 | 0.04512019 | 1 | UP |
| NONMMUT069252.2 | 9:56473527-56476121 | 2590 | -2.1225806 | 0.02288117 | 1 | DOWN |
| NONMMUT069579.2 | 9:64839376-64841073 | 1698 | 2.27799041 | 0.044282 | 1 | UP |
| NONMMUT069692.2 | 9:67014619-67019126 | 430 | 1.87188939 | 0.04053562 | 1 | UP |
| NONMMUT069796.2 | 9:68784401-68786652 | 2252 | Inf | 0.04880251 | 1 | UP |
| NONMMUT070169.2 | 9:79748803-79750051 | 1249 | -2.6096227 | 0.02736106 | 1 | DOWN |
| NONMMUT070464.2 | 9:94723500-94725762 | 2263 | #NAME? | 0.02987388 | 1 | DOWN |
| NONMMUT070869.2 | 9:106892049-106896269 | 3608 | Inf | 0.02118437 | 1 | UP |
| NONMMUT070915.2 | 9:107748521-107749911 | 1391 | -2.1334933 | 0.04298093 | 1 | DOWN |
| NONMMUT070994.2 | 9:108781091-108783472 | 2382 | Inf | 0.03894993 | 1 | UP |
| NONMMUT071374.2 | 9:119971167-119972453 | 1287 | 2.3367175 | 0.03828171 | 1 | UP |
| NONMMUT072313.2 | X:48170211-48171878 | 1668 | 2.49053147 | 0.02970005 | 1 | UP |
| NONMMUT073271.2 | X:98320176-98323212 | 3037 | 3.38234753 | 0.03377871 | 1 | UP |
| NONMMUT073412.2 | X:102493393-102495758 | 2366 | -1.2211762 | 0.04449433 | 1 | DOWN |
| NONMMUT074034.2 | X:137077280-137078690 | 1411 | 2.64525677 | 0.02500253 | 1 | UP |
| NONMMUT074985.1 | 1:6284204-6287193 | 2990 | -4.3946252 | 0.0249758 | 1 | DOWN |
| NONMMUT075142.1 | 1:23317115-23318520 | 1406 | -2.4504312 | 0.02621978 | 1 | DOWN |
| NONMMUT075163.1 | 1:24772360-24773193 | 834 | -2.1335558 | 0.02211633 | 1 | DOWN |
| NONMMUT075382.1 | 1:43597329-43598102 | 774 | #NAME? | 0.04015052 | 1 | DOWN |
| NONMMUT075843.1 | 1:82911568-82913997 | 2430 | -1.8244543 | 0.02600078 | 1 | DOWN |
| NONMMUT075846.1 | 1:82917460-82918670 | 1211 | -2.3560032 | 0.04268239 | 1 | DOWN |
| NONMMUT077049.1 | 1:187373268-187376147 | 2880 | -2.3659097 | 0.02220178 | 1 | DOWN |
| NONMMUT077121.1 | 1:191246961-191254358 | 7398 | Inf | 0.04324964 | 1 | UP |
| NONMMUT077131.1 | 1:191285148-191287493 | 2346 | -1.8392839 | 0.03744809 | 1 | DOWN |
| NONMMUT077235.1 | 1:4697932-4699088 | 1157 | #NAME? | 0.03032042 | 1 | DOWN |
| NONMMUT078104.1 | 1:79687424-79688891 | 1468 | -3.5263703 | 0.04300207 | 1 | DOWN |
| NONMMUT078174.1 | 1:86499917-86501803 | 1887 | #NAME? | 0.03791735 | 1 | DOWN |
| NONMMUT078284.1 | 1:97497834-97498768 | 935 | 2.75707405 | 0.02448135 | 1 | UP |
| NONMMUT079439.1 | 1:181082460-181084299 | 1840 | Inf | 0.03836725 | 1 | UP |
| NONMMUT079531.1 | 1:187025330-187026448 | 1119 | #NAME? | 0.021004 | 1 | DOWN |
| NONMMUT080287.1 | 10:63339183-63349055 | 1930 | #NAME? | 0.03939546 | 1 | DOWN |
| NONMMUT080774.1 | 10:96746199-96748478 | 2280 | -3.3059527 | 0.04293932 | 1 | DOWN |
| NONMMUT081348.1 | 10:9000882-9003400 | 2519 | Inf | 0.04330692 | 1 | UP |
| NONMMUT081381.1 | 10:9076466-9080829 | 4364 | Inf | 0.03440939 | 1 | UP |
| NONMMUT081382.1 | 10:9081101-9082151 | 1051 | Inf | 0.04326764 | 1 | UP |
| NONMMUT081432.1 | 10:9207382-9209271 | 1890 | Inf | 0.02149094 | 1 | UP |
| NONMMUT081438.1 | 10:9221263-9222204 | 942 | Inf | 0.02273112 | 1 | UP |
| NONMMUT081485.1 | 10:9401206-9403144 | 1939 | Inf | 0.02568265 | 1 | UP |
| NONMMUT081487.1 | 10:9409773-9418468 | 8696 | 2.73405549 | 0.0410554 | 1 | UP |
| NONMMUT081528.1 | 10:9550605-9552237 | 1633 | Inf | 0.02149057 | 1 | UP |
| NONMMUT081540.1 | 10:9580566-9586016 | 5451 | 2.56956286 | 0.02341632 | 1 | UP |
| NONMMUT081543.1 | 10:9590074-9591575 | 1502 | Inf | 0.02562614 | 1 | UP |
| NONMMUT081547.1 | 10:9607767-9613902 | 6136 | 2.56490618 | 0.02113448 | 1 | UP |
| NONMMUT082105.1 | 10:66898161-66900484 | 2324 | Inf | 0.0388995 | 1 | UP |
| NONMMUT083316.1 | 11:23075819-23077403 | 1585 | Inf | 0.03174622 | 1 | UP |
| NONMMUT083867.1 | 11:69384669-69385639 | 971 | Inf | 0.04330783 | 1 | UP |
| NONMMUT083989.1 | 11:78957761-78974391 | 546 | 1.96313188 | 0.02719626 | 1 | UP |
| NONMMUT084032.1 | 11:81967237-82006946 | 6194 | Inf | 0.02565511 | 1 | UP |
| NONMMUT084300.1 | 11:97193518-97195887 | 2370 | -3.6728149 | 0.02555188 | 1 | DOWN |
| NONMMUT085260.1 | 11:57557405-57558463 | 1059 | 2.10490523 | 0.04762374 | 1 | UP |
| NONMMUT085423.1 | 11:70654742-70656494 | 392 | #NAME? | 0.03793443 | 1 | DOWN |
| NONMMUT086077.1 | 11:120299085-120300993 | 1909 | -3.543439 | 0.04227098 | 1 | DOWN |
| NONMMUT086698.1 | 12:28989846-28994961 | 5116 | -2.5263197 | 0.02592684 | 1 | DOWN |
| NONMMUT086711.1 | 12:29017915-29019101 | 1187 | #NAME? | 0.03793898 | 1 | DOWN |
| NONMMUT086829.1 | 12:31351266-31383705 | 1005 | -2.8097285 | 0.043178 | 1 | DOWN |
| NONMMUT088020.1 | 12:22883396-22885185 | 1790 | #NAME? | 0.04264745 | 1 | DOWN |
| NONMMUT088199.1 | 12:32919385-32953790 | 2569 | 2.07473881 | 0.03572735 | 1 | UP |
| NONMMUT088222.1 | 12:34637811-34638508 | 698 | 2.99248745 | 0.02531828 | 1 | UP |
| NONMMUT088234.1 | 12:34673763-34674367 | 605 | 2.09552488 | 0.02239818 | 1 | UP |
| NONMMUT088239.1 | 12:34699646-34700446 | 801 | 2.22023398 | 0.03462718 | 1 | UP |
| NONMMUT088241.1 | 12:34706328-34707539 | 1212 | 1.48688827 | 0.03801402 | 1 | UP |
| NONMMUT088251.1 | 12:34755216-34756020 | 805 | 1.66137884 | 0.03057797 | 1 | UP |
| NONMMUT088258.1 | 12:34774807-34776069 | 1263 | 1.47699656 | 0.04761014 | 1 | UP |
| NONMMUT088263.1 | 12:34788731-34790231 | 1501 | 1.70052203 | 0.02998949 | 1 | UP |
| NONMMUT088269.1 | 12:34801175-34803541 | 2367 | 1.50183937 | 0.03270618 | 1 | UP |
| NONMMUT088934.1 | 12:101830808-101832029 | 1222 | -2.0803892 | 0.04576183 | 1 | DOWN |
| NONMMUT089384.1 | 13:22050935-22051747 | 813 | #NAME? | 0.03792337 | 1 | DOWN |
| NONMMUT089623.1 | 13:38986103-38997915 | 294 | Inf | 0.04330944 | 1 | UP |
| NONMMUT089640.1 | 13:40851788-40854544 | 2757 | 2.81735466 | 0.03558068 | 1 | UP |
| NONMMUT090273.1 | 13:75778114-75779157 | 1044 | -1.4949843 | 0.02167511 | 1 | DOWN |
| NONMMUT090864.1 | 13:15891364-15891992 | 629 | Inf | 0.03596307 | 1 | UP |
| NONMMUT091009.1 | 13:24799438-24801499 | 1843 | #NAME? | 0.04785998 | 1 | DOWN |
| NONMMUT091215.1 | 13:41596184-41596935 | 752 | #NAME? | 0.03878567 | 1 | DOWN |
| NONMMUT091400.1 | 13:51634327-51635714 | 1388 | 1.26014263 | 0.04059473 | 1 | UP |
| NONMMUT092116.1 | 13:98712263-98715078 | 2816 | 2.06650817 | 0.02588396 | 1 | UP |
| NONMMUT092196.1 | 13:103967764-103969039 | 1276 | -1.99372 | 0.03257859 | 1 | DOWN |
| NONMMUT092412.1 | 14:12326432-12327250 | 819 | 2.11771196 | 0.03997747 | 1 | UP |
| NONMMUT092654.1 | 14:30641369-30642567 | 1199 | -1.5226579 | 0.04898011 | 1 | DOWN |
| NONMMUT092738.1 | 14:40980901-40983894 | 2994 | #NAME? | 0.04614477 | 1 | DOWN |
| NONMMUT092879.1 | 14:49202004-49204859 | 2856 | -2.2990531 | 0.03533455 | 1 | DOWN |
| NONMMUT092891.1 | 14:49256804-49261988 | 5185 | 2.05762248 | 0.0389947 | 1 | UP |
| NONMMUT093617.1 | 14:105608340-105611637 | 3298 | -2.1892632 | 0.03387154 | 1 | DOWN |
| NONMMUT094619.1 | 14:73346171-73346777 | 607 | -2.3666114 | 0.02972143 | 1 | DOWN |
| NONMMUT095181.1 | 15:6023339-6024135 | 797 | -3.001413 | 0.0234094 | 1 | DOWN |
| NONMMUT095910.1 | 15:59619198-59624487 | 5290 | -1.7511611 | 0.04832354 | 1 | DOWN |
| NONMMUT096178.1 | 15:80221922-80223986 | 2065 | 3.40017148 | 0.03503075 | 1 | UP |
| NONMMUT096957.1 | 15:38468983-38470067 | 1085 | -1.5085653 | 0.02596413 | 1 | DOWN |
| NONMMUT097115.1 | 15:51773981-51775375 | 1395 | #NAME? | 0.02100134 | 1 | DOWN |
| NONMMUT097916.1 | 16:8902288-8907608 | 5321 | #NAME? | 0.03466675 | 1 | DOWN |
| NONMMUT098319.1 | 16:36101407-36103986 | 1321 | Inf | 0.02151303 | 1 | UP |
| NONMMUT099101.1 | 16:85626835-85627584 | 216 | -2.0885513 | 0.02330351 | 1 | DOWN |
| NONMMUT099360.1 | 16:97660142-97660774 | 633 | -2.5856985 | 0.02669147 | 1 | DOWN |
| NONMMUT099451.1 | 16:13715057-13730983 | 1524 | 1.96284955 | 0.03389637 | 1 | UP |
| NONMMUT100115.1 | 16:74959941-75123990 | 3029 | 2.8197238 | 0.04092046 | 1 | UP |
| NONMMUT100139.1 | 16:75056230-75060175 | 3946 | 2.62775107 | 0.04186732 | 1 | UP |
| NONMMUT100143.1 | 16:75070256-75073144 | 2889 | 3.1177949 | 0.02353693 | 1 | UP |
| NONMMUT100148.1 | 16:75080447-75082649 | 2203 | 3.0259759 | 0.0397422 | 1 | UP |
| NONMMUT100164.1 | 16:75124406-75125403 | 998 | 3.19707617 | 0.04694991 | 1 | UP |
| NONMMUT100171.1 | 16:75146967-75148216 | 1250 | 2.18006153 | 0.02848019 | 1 | UP |
| NONMMUT100191.1 | 16:75206425-75207164 | 740 | 3.22978329 | 0.03476641 | 1 | UP |
| NONMMUT100197.1 | 16:75214210-75214911 | 702 | 1.74701045 | 0.03412765 | 1 | UP |
| NONMMUT100233.1 | 16:75289869-75290952 | 1084 | 1.27260046 | 0.03818459 | 1 | UP |
| NONMMUT100367.1 | 16:88592092-88594118 | 405 | #NAME? | 0.03894969 | 1 | DOWN |
| NONMMUT100706.1 | 17:17249469-17250156 | 688 | #NAME? | 0.03793643 | 1 | DOWN |
| NONMMUT101956.1 | 17:28388233-28389998 | 1766 | 1.94571566 | 0.04907644 | 1 | UP |
| NONMMUT102111.1 | 17:43130444-43134820 | 4377 | #NAME? | 0.04613989 | 1 | DOWN |
| NONMMUT102156.1 | 17:46159560-46160937 | 724 | 3.47497782 | 0.04836521 | 1 | UP |
| NONMMUT102264.1 | 17:55930970-55932179 | 1210 | 3.44378419 | 0.04460397 | 1 | UP |
| NONMMUT103348.1 | 18:36317525-36324360 | 6831 | Inf | 0.03840225 | 1 | UP |
| NONMMUT103360.1 | 18:36336835-36338543 | 1709 | -3.5164743 | 0.02542704 | 1 | DOWN |
| NONMMUT103526.1 | 18:53976711-53978203 | 1493 | #NAME? | 0.02569461 | 1 | DOWN |
| NONMMUT103962.1 | 18:3241793-3242402 | 610 | -1.9035434 | 0.03247342 | 1 | DOWN |
| NONMMUT104041.1 | 18:6188544-6190467 | 1924 | 2.08716284 | 0.02509241 | 1 | UP |
| NONMMUT104049.1 | 18:6426485-6429063 | 2579 | 2.54968158 | 0.02056173 | 1 | UP |
| NONMMUT104107.1 | 18:12242157-12243728 | 1572 | #NAME? | 0.03791971 | 1 | DOWN |
| NONMMUT104320.1 | 18:34554447-34555239 | 793 | 3.79973422 | 0.03117776 | 1 | UP |
| NONMMUT104719.1 | 18:62150208-62152195 | 1988 | Inf | 0.04871777 | 1 | UP |
| NONMMUT104866.1 | 18:74728984-74733453 | 831 | #NAME? | 0.03894724 | 1 | DOWN |
| NONMMUT105559.1 | 19:44321641-44322362 | 722 | #NAME? | 0.03939814 | 1 | DOWN |
| NONMMUT105569.1 | 19:44588017-44589156 | 1140 | Inf | 0.03257974 | 1 | UP |
| NONMMUT105796.1 | 19:4411341-4412680 | 1340 | -1.1973033 | 0.0402494 | 1 | DOWN |
| NONMMUT105799.1 | 19:4416241-4417633 | 1393 | -1.1798764 | 0.03846992 | 1 | DOWN |
| NONMMUT105832.1 | 19:6820079-6823246 | 3168 | 3.34959094 | 0.022289 | 1 | UP |
| NONMMUT105891.1 | 19:10491756-10493470 | 1715 | -1.7999912 | 0.04378217 | 1 | DOWN |
| NONMMUT106121.1 | 19:32115867-32116877 | 1011 | -2.03599 | 0.04044235 | 1 | DOWN |
| NONMMUT106591.1 | 2:6466085-6478620 | 1166 | -2.1521795 | 0.04086257 | 1 | DOWN |
| NONMMUT107487.1 | 2:90479099-90488426 | 5870 | -2.0489387 | 0.03492286 | 1 | DOWN |
| NONMMUT107507.1 | 2:92762328-92763687 | 1360 | 3.27564922 | 0.04696006 | 1 | UP |
| NONMMUT107862.1 | 2:127304163-127308290 | 4128 | -1.7099464 | 0.04863184 | 1 | DOWN |
| NONMMUT107945.1 | 2:131358869-131359570 | 702 | #NAME? | 0.04422868 | 1 | DOWN |
| NONMMUT108081.1 | 2:146976275-146978280 | 2006 | #NAME? | 0.03153922 | 1 | DOWN |
| NONMMUT108133.1 | 2:151542483-151561691 | 1544 | 3.28986214 | 0.03070228 | 1 | UP |
| NONMMUT108677.1 | 2:11330348-11331851 | 1413 | Inf | 0.03838398 | 1 | UP |
| NONMMUT109198.1 | 2:72605311-72615220 | 917 | 1.88669986 | 0.0289492 | 1 | UP |
| NONMMUT109934.1 | 2:151531341-151532267 | 927 | #NAME? | 0.03924884 | 1 | DOWN |
| NONMMUT110248.1 | 2:170338126-170340328 | 2203 | #NAME? | 0.03152711 | 1 | DOWN |
| NONMMUT111151.1 | 3:57853579-57858172 | 4594 | #NAME? | 0.02515714 | 1 | DOWN |
| NONMMUT111882.1 | 3:106057945-106059295 | 1351 | 3.51856138 | 0.04874845 | 1 | UP |
| NONMMUT112078.1 | 3:122293776-122305264 | 2357 | 2.6689676 | 0.02424051 | 1 | UP |
| NONMMUT112600.1 | 3:10409293-10409937 | 645 | 2.6304776 | 0.04695953 | 1 | UP |
| NONMMUT112926.1 | 3:24741923-24746093 | 4171 | 2.35312789 | 0.02580882 | 1 | UP |
| NONMMUT114718.1 | 4:20071164-20073883 | 2720 | -2.0085761 | 0.030714 | 1 | DOWN |
| NONMMUT115004.1 | 4:55540824-55541751 | 928 | -1.2555659 | 0.04751352 | 1 | DOWN |
| NONMMUT115050.1 | 4:62736858-62737751 | 894 | #NAME? | 0.0239978 | 1 | DOWN |
| NONMMUT115096.1 | 4:72059560-72065516 | 836 | #NAME? | 0.03793517 | 1 | DOWN |
| NONMMUT115985.1 | 4:148960298-148984672 | 5876 | #NAME? | 0.02515713 | 1 | DOWN |
| NONMMUT116668.1 | 4:48027634-48038003 | 10370 | 1.63539965 | 0.03517001 | 1 | UP |
| NONMMUT116962.1 | 4:94453533-94456562 | 3030 | -1.9007434 | 0.04738192 | 1 | DOWN |
| NONMMUT117757.1 | 4:149435549-149437175 | 1627 | 1.26626492 | 0.02719393 | 1 | UP |
| NONMMUT118154.1 | 5:30546121-30552593 | 658 | #NAME? | 0.03153818 | 1 | DOWN |
| NONMMUT118159.1 | 5:30680128-30681637 | 1510 | -1.8535085 | 0.02664656 | 1 | DOWN |
| NONMMUT118184.1 | 5:32090539-32094052 | 1815 | #NAME? | 0.02121396 | 1 | DOWN |
| NONMMUT118274.1 | 5:37156389-37157385 | 997 | Inf | 0.03898199 | 1 | UP |
| NONMMUT118506.1 | 5:64494111-64495175 | 1065 | #NAME? | 0.04191873 | 1 | DOWN |
| NONMMUT120188.1 | 5:33234905-33235917 | 1013 | -2.3894692 | 0.04313392 | 1 | DOWN |
| NONMMUT122136.1 | 6:42420126-42420994 | 869 | #NAME? | 0.02209204 | 1 | DOWN |
| NONMMUT122239.1 | 6:50681305-50700250 | 514 | Inf | 0.03893924 | 1 | UP |
| NONMMUT122255.1 | 6:51597165-51601084 | 3920 | 1.69225107 | 0.04469896 | 1 | UP |
| NONMMUT122257.1 | 6:51602105-51603928 | 1824 | 3.74381202 | 0.04283474 | 1 | UP |
| NONMMUT122315.1 | 6:54667626-54668545 | 920 | #NAME? | 0.03105277 | 1 | DOWN |
| NONMMUT122364.1 | 6:59246315-59247121 | 807 | Inf | 0.04330619 | 1 | UP |
| NONMMUT122629.1 | 6:88927863-88928639 | 777 | #NAME? | 0.0465881 | 1 | DOWN |
| NONMMUT122634.1 | 6:88942300-88944390 | 2091 | Inf | 0.04872838 | 1 | UP |
| NONMMUT122905.1 | 6:117933586-117934568 | 891 | #NAME? | 0.0311799 | 1 | DOWN |
| NONMMUT123141.1 | 6:135266221-135269231 | 3011 | -1.8909203 | 0.0351983 | 1 | DOWN |
| NONMMUT123372.1 | 6:3333411-3336037 | 2627 | 1.13176778 | 0.03924986 | 1 | UP |
| NONMMUT123552.1 | 6:31366886-31398774 | 3193 | -2.3301371 | 0.04590622 | 1 | DOWN |
| NONMMUT123568.1 | 6:32767765-32769521 | 1757 | -2.0914256 | 0.02768973 | 1 | DOWN |
| NONMMUT123628.1 | 6:39245072-39247088 | 2017 | 1.96580668 | 0.04447394 | 1 | UP |
| NONMMUT124181.1 | 6:98909519-98910179 | 661 | -2.8595942 | 0.03007445 | 1 | DOWN |
| NONMMUT124357.1 | 6:118334416-118335991 | 1576 | 2.46081064 | 0.02716696 | 1 | UP |
| NONMMUT125485.1 | 7:79920756-79924378 | 1856 | #NAME? | 0.03825973 | 1 | DOWN |
| NONMMUT125904.1 | 7:100673944-100674940 | 997 | #NAME? | 0.02175989 | 1 | DOWN |
| NONMMUT126041.1 | 7:117677080-117678539 | 1460 | #NAME? | 0.02905191 | 1 | DOWN |
| NONMMUT126215.1 | 7:127855050-127856970 | 1724 | #NAME? | 0.03135563 | 1 | DOWN |
| NONMMUT126779.1 | 7:44579969-44590625 | 2243 | -3.6122159 | 0.0349515 | 1 | DOWN |
| NONMMUT126808.1 | 7:48723625-48734655 | 1259 | Inf | 0.0240367 | 1 | UP |
| NONMMUT127160.1 | 7:65432569-65434345 | 1777 | Inf | 0.0214914 | 1 | UP |
| NONMMUT127744.1 | 7:124625671-124708936 | 791 | -2.6794415 | 0.03879235 | 1 | DOWN |
| NONMMUT127966.1 | 7:145233127-145238432 | 5205 | -1.2354845 | 0.0487317 | 1 | DOWN |
| NONMMUT128184.1 | 8:12275348-12276327 | 980 | -2.169126 | 0.03956181 | 1 | DOWN |
| NONMMUT129316.1 | 8:105297338-105299517 | 816 | -2.1429136 | 0.04332868 | 1 | DOWN |
| NONMMUT129816.1 | 8:126537241-126538539 | 1299 | #NAME? | 0.02985092 | 1 | DOWN |
| NONMMUT129828.1 | 8:127626658-127628137 | 1480 | #NAME? | 0.03219084 | 1 | DOWN |
| NONMMUT130135.1 | 8:27244367-27250887 | 5241 | -3.0213189 | 0.02905853 | 1 | DOWN |
| NONMMUT130353.1 | 8:32534857-32544112 | 9256 | 3.36882688 | 0.02263176 | 1 | UP |
| NONMMUT130355.1 | 8:32546502-32554499 | 7998 | 3.30319948 | 0.04438883 | 1 | UP |
| NONMMUT130376.1 | 8:32602796-32605709 | 2914 | Inf | 0.04289785 | 1 | UP |
| NONMMUT130448.1 | 8:32838117-32843093 | 4977 | Inf | 0.03125184 | 1 | UP |
| NONMMUT131730.1 | 8:115622633-115630775 | 8143 | #NAME? | 0.02985455 | 1 | DOWN |
| NONMMUT132021.1 | 9:13555646-13558183 | 2538 | Inf | 0.0432626 | 1 | UP |
| NONMMUT132033.1 | 9:13716550-13717924 | 1375 | Inf | 0.04875582 | 1 | UP |
| NONMMUT132247.1 | 9:32018537-32019625 | 1089 | 2.44313679 | 0.03833054 | 1 | UP |
| NONMMUT132248.1 | 9:32023520-32024557 | 1038 | 2.2587629 | 0.02418335 | 1 | UP |
| NONMMUT132258.1 | 9:32055352-32058839 | 3488 | 1.27123003 | 0.03241751 | 1 | UP |
| NONMMUT132260.1 | 9:32061276-32062607 | 1332 | 1.52190147 | 0.02884764 | 1 | UP |
| NONMMUT132268.1 | 9:32087923-32088923 | 1001 | 1.75726051 | 0.0342046 | 1 | UP |
| NONMMUT132269.1 | 9:32089009-32090836 | 1828 | 1.31473687 | 0.04515215 | 1 | UP |
| NONMMUT132272.1 | 9:32101486-32104465 | 2980 | 1.31427525 | 0.02344604 | 1 | UP |
| NONMMUT132473.1 | 9:45300672-45301424 | 753 | 3.40017148 | 0.03503586 | 1 | UP |
| NONMMUT133294.1 | 9:94554028-94564540 | 10513 | -1.4893131 | 0.04892491 | 1 | DOWN |
| NONMMUT133554.1 | 9:111183658-111205779 | 388 | 2.54633558 | 0.02907985 | 1 | UP |
| NONMMUT133982.1 | 9:25098713-25099707 | 995 | -2.0244984 | 0.03324843 | 1 | DOWN |
| NONMMUT134031.1 | 9:30968539-30970191 | 1653 | #NAME? | 0.02818498 | 1 | DOWN |
| NONMMUT134379.1 | 9:56504935-56507590 | 2656 | #NAME? | 0.03793494 | 1 | DOWN |
| NONMMUT135616.1 | 9:122294719-122310929 | 4294 | -1.3217833 | 0.02527084 | 1 | DOWN |
| NONMMUT136742.1 | X:13346229-13347525 | 1297 | Inf | 0.02345095 | 1 | UP |
| NONMMUT136762.1 | X:17481555-17483081 | 1527 | #NAME? | 0.03794279 | 1 | DOWN |
| NONMMUT136859.1 | X:38524315-38525813 | 1499 | #NAME? | 0.03219959 | 1 | DOWN |
| NONMMUT138533.1 | 1:23256286-23257398 | 1113 | 2.8934125 | 0.02076137 | 1 | UP |
| NONMMUT138837.1 | 1:171066639-171066978 | 340 | #NAME? | 0.02175067 | 1 | DOWN |
| NONMMUT138883.1 | 1:180260621-180265552 | 4932 | 2.64660122 | 0.03175061 | 1 | UP |
| NONMMUT139039.1 | 1:60067986-60074059 | 3639 | #NAME? | 0.02638599 | 1 | DOWN |
| NONMMUT139448.1 | 10:44429183-44432599 | 2261 | Inf | 0.04670887 | 1 | UP |
| NONMMUT140348.1 | 11:20226961-20240109 | 3826 | Inf | 0.03005797 | 1 | UP |
| NONMMUT140408.1 | 11:48823480-48824160 | 681 | #NAME? | 0.04649236 | 1 | DOWN |
| NONMMUT140452.1 | 11:60791025-60795271 | 3226 | #NAME? | 0.02772745 | 1 | DOWN |
| NONMMUT140868.1 | 11:43723248-43726185 | 2938 | -1.0872163 | 0.04319554 | 1 | DOWN |
| NONMMUT141190.1 | 11:119932773-119939854 | 5092 | #NAME? | 0.02263594 | 1 | DOWN |
| NONMMUT141204.1 | 11:120182623-120189891 | 5765 | Inf | 0.02345098 | 1 | UP |
| NONMMUT141419.1 | 12:52603801-52604246 | 446 | 3.07074435 | 0.04717761 | 1 | UP |
| NONMMUT141484.1 | 12:73822583-73837394 | 331 | #NAME? | 0.04786672 | 1 | DOWN |
| NONMMUT141996.1 | 12:85473028-85473917 | 890 | -2.25938 | 0.0466684 | 1 | DOWN |
| NONMMUT142531.1 | 13:107022550-107033611 | 2326 | Inf | 0.04287441 | 1 | UP |
| NONMMUT142659.1 | 13:34161022-34162306 | 1285 | Inf | 0.0214829 | 1 | UP |
| NONMMUT143034.1 | 14:25701384-25703666 | 598 | #NAME? | 0.04423085 | 1 | DOWN |
| NONMMUT143179.1 | 14:63544113-63544652 | 540 | 3.48185204 | 0.03669914 | 1 | UP |
| NONMMUT143316.1 | 14:7817408-7817951 | 544 | 3.44404542 | 0.02977945 | 1 | UP |
| NONMMUT143463.1 | 14:57082069-57090230 | 8162 | -3.6073211 | 0.02316407 | 1 | DOWN |
| NONMMUT144959.1 | 17:69416217-69419277 | 2918 | #NAME? | 0.03580602 | 1 | DOWN |
| NONMMUT145254.1 | 17:84133783-84137354 | 1505 | 3.55378208 | 0.02384585 | 1 | UP |
| NONMMUT145277.1 | 17:84145853-84156440 | 728 | #NAME? | 0.0343585 | 1 | DOWN |
| NONMMUT145584.1 | 18:35255854-35258577 | 2724 | #NAME? | 0.02176571 | 1 | DOWN |
| NONMMUT145721.1 | 18:75358508-75367098 | 3754 | #NAME? | 0.02376891 | 1 | DOWN |
| NONMMUT146124.1 | 19:43948658-43951814 | 3157 | -1.3455879 | 0.02042891 | 1 | DOWN |
| NONMMUT146275.1 | 2:37430992-37431744 | 753 | Inf | 0.04326512 | 1 | UP |
| NONMMUT146420.1 | 2:119594296-119607721 | 2672 | 2.3686079 | 0.0300139 | 1 | UP |
| NONMMUT147058.1 | 2:147944002-147987869 | 4382 | #NAME? | 0.02492066 | 1 | DOWN |
| NONMMUT147064.1 | 2:147944002-148040244 | 1696 | #NAME? | 0.02712712 | 1 | DOWN |
| NONMMUT147205.1 | 2:167768775-167783511 | 14339 | Inf | 0.02909288 | 1 | UP |
| NONMMUT147507.1 | 3:107908011-107920125 | 1267 | -1.7291602 | 0.03069116 | 1 | DOWN |
| NONMMUT147726.1 | 3:89289716-89292947 | 2950 | #NAME? | 0.0358002 | 1 | DOWN |
| NONMMUT148047.1 | 4:55533709-55542044 | 3320 | 1.8160314 | 0.02297049 | 1 | UP |
| NONMMUT148186.1 | 4:118427305-118427722 | 418 | #NAME? | 0.03793136 | 1 | DOWN |
| NONMMUT148340.1 | 4:150672361-150677237 | 4877 | -2.9560886 | 0.03555093 | 1 | DOWN |
| NONMMUT148397.1 | 4:3874675-3875245 | 571 | Inf | 0.02345383 | 1 | UP |
| NONMMUT148409.1 | 4:10957938-10965577 | 7185 | #NAME? | 0.03117905 | 1 | DOWN |
| NONMMUT148598.1 | 4:108627485-108630095 | 2353 | #NAME? | 0.0373232 | 1 | DOWN |
| NONMMUT148614.1 | 4:109402287-109406957 | 3111 | Inf | 0.02150657 | 1 | UP |
| NONMMUT148631.1 | 4:111719107-111719739 | 633 | Inf | 0.03178141 | 1 | UP |
| NONMMUT148880.1 | 5:20895076-20905782 | 1997 | #NAME? | 0.04785484 | 1 | DOWN |
| NONMMUT148915.1 | 5:31179258-31180420 | 760 | Inf | 0.02527249 | 1 | UP |
| NONMMUT149054.1 | 5:105826940-105829906 | 2821 | Inf | 0.03141524 | 1 | UP |
| NONMMUT149776.1 | 5:140416311-140419451 | 3141 | #NAME? | 0.0248572 | 1 | DOWN |
| NONMMUT150231.1 | 6:143100681-143101372 | 692 | #NAME? | 0.04421631 | 1 | DOWN |
| NONMMUT150347.1 | 6:31178471-31179169 | 699 | 1.74096408 | 0.04087716 | 1 | UP |
| NONMMUT150711.1 | 6:129207844-129238061 | 2773 | 3.24668325 | 0.02837477 | 1 | UP |
| NONMMUT150864.1 | 7:28338856-28339480 | 625 | #NAME? | 0.03152126 | 1 | DOWN |
| NONMMUT150956.1 | 7:44307211-44308258 | 1048 | Inf | 0.03893255 | 1 | UP |
| NONMMUT151116.1 | 7:81523550-81531606 | 7651 | 2.49938328 | 0.03096781 | 1 | UP |
| NONMMUT151179.1 | 7:97788517-97788932 | 416 | -3.0643227 | 0.02207123 | 1 | DOWN |
| NONMMUT151470.1 | 7:19417466-19421417 | 2560 | #NAME? | 0.03793667 | 1 | DOWN |
| NONMMUT152010.1 | 8:15039027-15039762 | 595 | -1.6573277 | 0.0366811 | 1 | DOWN |
| NONMMUT152014.1 | 8:15519546-15538690 | 494 | -2.5557408 | 0.04418864 | 1 | DOWN |
| NONMMUT152049.1 | 8:24412473-24416934 | 3140 | -1.26165 | 0.0371117 | 1 | DOWN |
| NONMMUT152447.1 | 8:122035316-122036079 | 764 | -3.4795558 | 0.02935468 | 1 | DOWN |
| NONMMUT152512.1 | 8:10897257-10910621 | 7175 | Inf | 0.04822318 | 1 | UP |
| NONMMUT152596.1 | 8:26969920-26971612 | 1693 | 2.15933347 | 0.04678585 | 1 | UP |
| NONMMUT153270.1 | 9:57766442-57767977 | 825 | 4.21471051 | 0.04341433 | 1 | UP |
| NONMMUT153437.1 | 9:103092143-103092494 | 352 | -2.2196785 | 0.03795834 | 1 | DOWN |
| NONMMUT153768.1 | 9:61368447-61370428 | 1553 | Inf | 0.03894118 | 1 | UP |
| NONMMUT153770.1 | 9:61803905-61805011 | 1107 | -1.3286029 | 0.03543824 | 1 | DOWN |
| NONMMUT154339.1 | X:105037028-105070124 | 4767 | 1.87155272 | 0.0355966 | 1 | UP |

**TABLE S3** 20 mRNAs selected for ceRNA construction

| Gene name | Locus | Log2 FC | P value | Q value | Regulation |
| --- | --- | --- | --- | --- | --- |
| Slc8a1 | chr17:81388691-81649607 | - | 7.56E-08 | 0.000107 | Up |
| Sema4a | chr3:88435962-88458884 | - | 1.76E-07 | 0.000202 | Up |
| Eif2s2 | chr2:154871418-154892782 | - | 1.61E-07 | 0.000197 | Up |
| Gtf3c1 | chr7:125640954-125707677 | - | 3.57E-08 | 5.77E-05 | Up |
| St6galnac6 | chr2:32606961-32620804 | 12.4384547 | 4.26E-07 | 0.000409 | Up |
| Rnf4 | chr5:34336932-34353424 | 9.488913 | 1.57E-08 | 2.94E-05 | Up |
| Clec4e | chr6:123282668-123289870 | 8.99799658 | 9.64E-11 | 3.81E-07 | Up |
| Tnip3 | chr6:65525313-65631786 | 8.72884415 | 0.000113 | 0.02355 | Up |
| Prkd3 | chr17:78949405-79013306 | 8.51644117 | 6.99E-11 | 3.11E-07 | Up |
| Hnrnpu | chr1:178323074-178337728 | 8.50367286 | 0.0002376 | 0.040112 | Up |
| Nfkbiz | chr16:55811378-55838854 | - | 1.07E-08 | 2.17E-05 | Down |
| Prpf8 | chr11:75486820-75509449 | - | 2.98E-29 | 2.12E-24 | Down |
| Arhgap21 | chr2:20848594-20968526 | - | 2.75E-08 | 4.66E-05 | Down |
| Elmo3 | chr8:105305613-105309977 | - | 8.41E-08 | 0.000115 | Down |
| Coro2a | chr4:46536943-46566458 | - | 4.99E-07 | 0.00046 | Down |
| Dnmbp | chr19:43847967-43912392 | - | 1.42E-16 | 2.02E-12 | Down |
| Specc1 | chr11:62018848-62141726 | -9.4933281 | 0.0001421 | 0.02768 | Down |
| Pip5k1c | chr10:81293004-81319972 | -9.2757546 | 1.45E-06 | 0.001033 | Down |
| Sipa1l1 | chr12:82256585-82450271 | -8.8148861 | 2.82E-07 | 0.000295 | Down |
| Ahcyl2 | chr6:29859379-29912304 | -8.5374742 | 2.46E-05 | 0.008581 | Down |

**TABLE S4** Top 20 core genes and their corresponding degree

| Gene | Degree |  | Gene | Degree |
| --- | --- | --- | --- | --- |
| Mmp9 | 27 |  | Sparc | 14 |
| Fpr2 | 17 |  | Fpr1 | 14 |
| Ccl3 | 17 |  | Mmp8 | 14 |
| Col1a2 | 15 |  | Clec4d | 14 |
| Col1a1 | 15 |  | Lyz2 | 14 |
| Cxcr2 | 15 |  | Lilrb4 | 14 |
| Sptan1 | 15 |  | Csf1r | 14 |
| Ptgs2 | 15 |  | Col5a2 | 13 |
| C5ar1 | 15 |  | Ppbp | 13 |
| Col3a1 | 14 |  | Plek | 13 |
